# Supplementary material for: Prehospital acute life-threatening cardiovascular disease in elderly: an observational, prospective, multicentre, ambulance-based cohort study
Source: BMJ Open. 2023 Nov 23;13(11):e078815. doi: 10.1136/bmjopen-2023-078815 (PMC10668192; doi:10.1136/bmjopen-2023-078815)
Supplement: Supplementary data [file bmjopen-2023-078815supp001.pdf]

BMJ Publishing Group Limited (BMJ) disclaims all liability and responsibility arising from any reliance placed on this supplemental material which has been supplied by the author(s)

Supplementary data

del Pozo Vegas C, *et al. BMJ Open* 2023; 13:e078815. doi: 10.1136/bmjopen-2023-078815

Table of contents

Supplementary methods…..……………………………………………………………..3
Strengthening the Reporting of Observational Studies in Epidemiology (STROBE) Statement…..………..…………………………………………..…3
Supplementary Results………………………………………………………….……5
- Supplementary Figure 1. Kaplan Meier survival curves according to cardiovascular diagnosis and age……………………………………………...5
- Supplementary Table S1. Cumulative mortality by prehospital syndromic suspect……………………………………………………………………………6
- Supplementary Table S2: Summary descriptives table by groups of acute heart failure..…..………………………………………………………...........7
- Supplementary Table S3: Summary descriptives table by groups of Arrythmmia...…………………………………………………………………8
- Supplementary Table S4: Summary descriptives table by groups of Hypertensive emergency……………………………...................................9
- Supplementary Table S5 Summary descriptives table by groups of ischemic heart disease…….………..……………………………………………………..10
- Supplementary Table S6: Summary descriptives table by groups of syncope..11
- Supplementary Table S7: Log-regression table by mortality groups…………12
References...14

Supplementary Methods

STROBE[1] Statement—checklist of items that should be included in reports of observational studies

	Item No	Recommendation	Page No
**Title and abstract**	1	(*a*) Indicate the study's design with a commonly used term in the title or the abstract	1
		(*b*) Provide in the abstract an informative and balanced summary of what was done and what was found	3
**Introduction**			
Background/rationale	2	Explain the scientific background and rationale for the investigation being reported	5
Objectives	3	State specific objectives, including any prespecified hypotheses	5
**Methods**			
Study design	4	Present key elements of study design early in the paper	6
Setting	5	Describe the setting, locations, and relevant dates, including periods of recruitment, exposure, follow-up, and data collection	6
Participants	6	(*a*) *Cohort study*—Give the eligibility criteria, and the sources and methods of selection of participants. Describe methods of follow-up<br>*Case-control study*—Give the eligibility criteria, and the sources and methods of case ascertainment and control selection. Give the rationale for the choice of cases and controls<br>*Cross-sectional study*—Give the eligibility criteria, and the sources and methods of selection of participants	6
		(*b*) *Cohort study*—For matched studies, give matching criteria and number of exposed and unexposed<br>*Case-control study*—For matched studies, give matching criteria and the number of controls per case	6
Variables	7	Clearly define all outcomes, exposures, predictors, potential confounders, and effect modifiers. Give diagnostic criteria, if applicable	7
Data sources/ measurement	8*	For each variable of interest, give sources of data and details of methods of assessment (measurement). Describe comparability of assessment methods if there is more than one group	*7*
Bias	9	Describe any efforts to address potential sources of bias	7
Study size	10	Explain how the study size was arrived at	7
Quantitative variables	11	Explain how quantitative variables were handled in the analyses. If applicable, describe which groupings were chosen and why	7
Statistical methods	12	(*a*) Describe all statistical methods, including those used to control for confounding	7
		(*b*) Describe any methods used to examine subgroups and interactions	7
		(*c*) Explain how missing data were addressed	7
		(*d*) *Cohort study*—If applicable, explain how loss to follow-up was addressed<br>*Case-control study*—If applicable, explain how matching of cases and controls was addressed<br>*Cross-sectional study*—If applicable, describe analytical methods taking account of sampling strategy	7
		(*e*) Describe any sensitivity analyses	7

Continued on next page

**Results**			
Participants	13*	(a) Report numbers of individuals at each stage of study—eg numbers potentially eligible, examined for eligibility, confirmed eligible, included in the study, completing follow-up, and analysed	8
		(b) Give reasons for non-participation at each stage	8
		(c) Consider use of a flow diagram	8
Descriptive data	14*	(a) Give characteristics of study participants (eg demographic, clinical, social) and information on exposures and potential confounders	8
		(b) Indicate number of participants with missing data for each variable of interest	8
		(c) *Cohort study*—Summarise follow-up time (eg, average and total amount)	8
Outcome data	15*	*Cohort study*—Report numbers of outcome events or summary measures over time	*8*
		Case-control study—Report numbers in each exposure category, or summary measures of exposure	*8*
		Cross-sectional study—Report numbers of outcome events or summary measures	*8*
Main results	16	(*a*) Give unadjusted estimates and, if applicable, confounder-adjusted estimates and their precision (eg, 95% confidence interval). Make clear which confounders were adjusted for and why they were included	9
		(*b*) Report category boundaries when continuous variables were categorized	9
		(*c*) If relevant, consider translating estimates of relative risk into absolute risk for a meaningful time period	9
Other analyses	17	Report other analyses done—eg analyses of subgroups and interactions, and sensitivity analyses	9
**Discussion**			
Key results	18	Summarise key results with reference to study objectives	10-11
Limitations	19	Discuss limitations of the study, taking into account sources of potential bias or imprecision. Discuss both direction and magnitude of any potential bias	11-12
Interpretation	20	Give a cautious overall interpretation of results considering objectives, limitations, multiplicity of analyses, results from similar studies, and other relevant evidence	10,11,12
Generalisability	21	Discuss the generalisability (external validity) of the study results	11,12,13
**Other information**			
Funding	22	Give the source of funding and the role of the funders for the present study and, if applicable, for the original study on which the present article is based	2

*Give information separately for cases and controls in case-control studies and, if applicable, for exposed and unexposed groups in cohort and cross-sectional studies.

Note: An Explanation and Elaboration article discusses each checklist item and gives methodological background and published examples of transparent reporting. The STROBE checklist is best used in conjunction with this article (freely available on the Web sites of PLoS Medicine at http://www.plosmedicine.org/, Annals of Internal Medicine at http://www.annals.org/, and Epidemiology at http://www.epidem.com/). Information on the STROBE Initiative is available at www.strobe-statement.org.

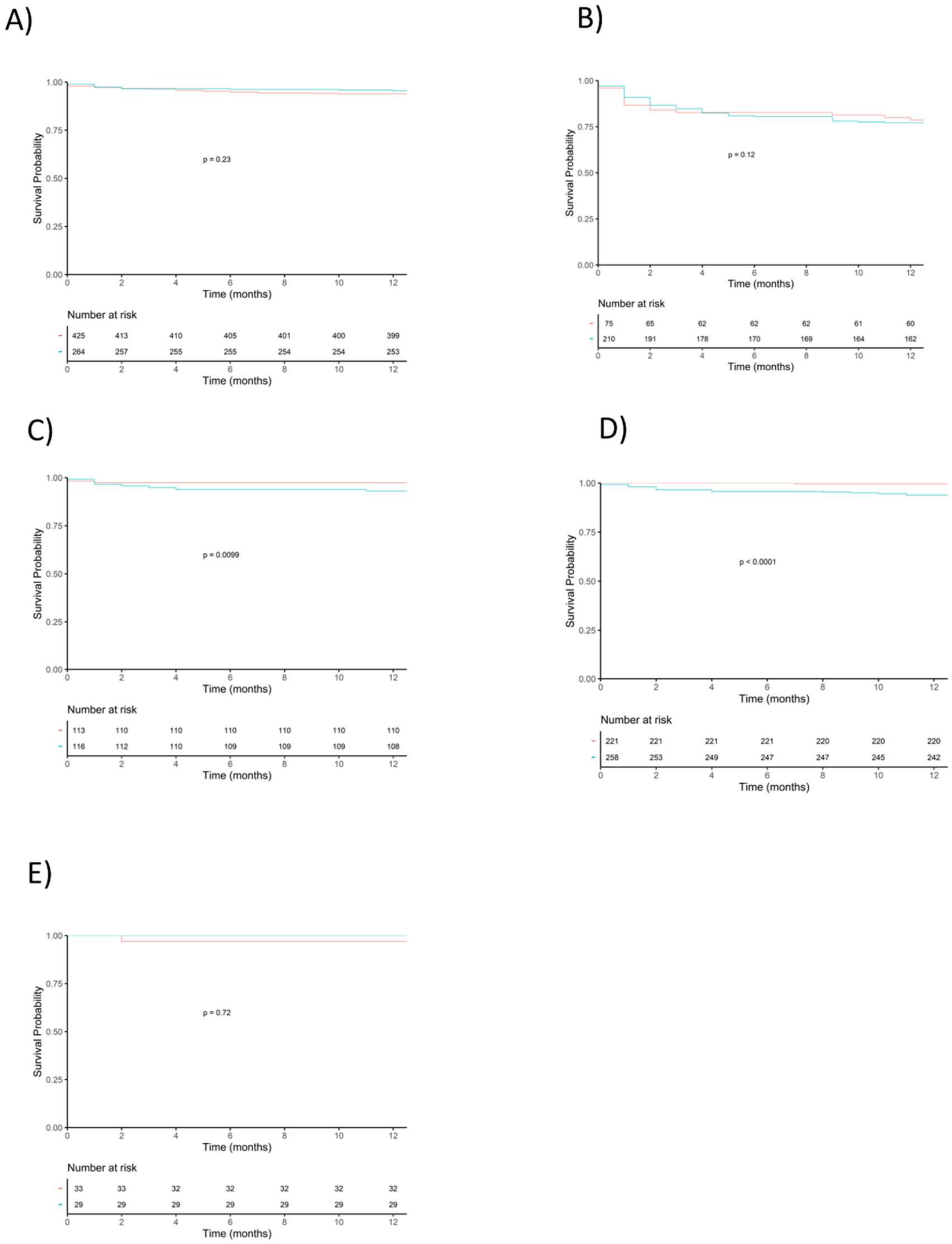

Supplementary Figure S1. Kaplan Meier survival curves for Age <75 years vs ≥ 75 years according to cardiovascular diagnosis. Ischemic heart disease (A), acute heart failure (B), arrhythmia (C), syncope (D), hypertensive emergency (E). Age ≤ 74 years (red line), and age ≥ 75 years (blue line).

Supplementary Table S1. Cumulative mortality by prehospital syndromic suspect

Variable[a]	Ischemic heart disease	Acute heart failure	Arrhythmia	Syncope	Hypertensive emergency	*p* value[b]
≤ 74 years	425 (49)	76 (8.8)	112 (12.9)	221 (25.2)	33 (3.8)	
1-day	12 (2.8)	10 (13.2)	3 (2.7)	0	0	0.022
2-day	14 (3.3)	12 (15.8)	3 (2.7)	0	1 (3)	0.033
7-day	24 (5.6)	13 (17.1)	3 (2.7)	1 (0.5)	1 (3)	0.002
30-day	26 (6.1)	21 (27.6)	3 (2.7)	2 (0.9)	1 (3)	0.002
90-day	33 (7.8)	26 (23.2)	7 (6.3)	7 (3.2)	1 (3)	0.012
180-day	38 (8.9)	28 (36.8)	9 (8)	10 (4.5)	3 (9.1)	0.060
365-day	43 (10.1)	33 (43.4)	10 (8.9)	12 (5.4)	3 (9.1)	0.042
≥ 75 years	264 (30.1)	210 (23.9)	116 (13.2)	258 (29.4)	29 (3.3)	
1-day	7 (2.7)	19 (9)	4 (3.4)	5 (1.9)	0	0.133
2-day	9 (3.4)	28 (13.3)	5 (4.3)	9 (3.5)	0	0.161
7-day	10 (3.8)	41 (19.5)	7 (6)	11 (4.3)	0	0.100
30-day	17 (6.4)	60 (28.6)	11 (9.5)	18 (7)	0	0.043
90-day	22 (8.3)	86 (41)	16 (13.8)	32 (12.4)	0	0.114
180-day	25 (9.5)	99 (47.1)	20 (17.2)	40 (15.5)	0	0.185
365-day	35 (13.3)	115 (54.8)	26 (22.4)	51 (19.8)	2 (6.9)	0.235

[a]Values expressed as total number (percentage).

[b]Fisher's Exact Probability statistic, was used.

del Pozo Vegas C, *et al. BMJ Open* 2023; 13:e078815. doi: 10.1136/bmjopen-2023-078815

Supplementary Table S2: Summary descriptives table by groups of acute heart failure

	Not Suffering acute heart failure *N=75*	Suffering acute heart failure *N=210*	OR	p.ratio	p.overall
1-day:					0.406
0	65 (86.7%)	191 (91.0%)	Ref.	Ref.	
1	10 (13.3%)	19 (9.05%)	0.64 [0.29;1.52]	0.304	
2-day:					0.706
0	63 (84.0%)	182 (86.7%)	Ref.	Ref.	
1	12 (16.0%)	28 (13.3%)	0.80 [0.39;1.74]	0.566	
7-day:					0.807
0	62 (82.7%)	169 (80.5%)	Ref.	Ref.	
1	13 (17.3%)	41 (19.5%)	1.15 [0.59;2.37]	0.693	
30-day:					1.000
0	54 (72.0%)	150 (71.4%)	Ref.	Ref.	
1	21 (28.0%)	60 (28.6%)	1.03 [0.57;1.88]	0.934	
90-day:					0.306
0	50 (66.7%)	124 (59.0%)	Ref.	Ref.	
1	25 (33.3%)	86 (41.0%)	1.38 [0.80;2.44]	0.249	
180-day:					0.125
0	48 (64.0%)	111 (52.9%)	Ref.	Ref.	
1	27 (36.0%)	99 (47.1%)	1.58 [0.92;2.75]	0.097	
365-day:					0.096
0	43 (57.3%)	95 (45.2%)	Ref.	Ref.	
1	32 (42.7%)	115 (54.8%)	1.62 [0.95;2.78]	0.074	

0: survival; 1: non-survival

Supplementary Table S3: Summary descriptives table by groups of Arrythmia

	Not Suffering Arrythmia *N=113*	Suffering Arrythmia *N=116*	OR	p.ratio	p.overall
1-day:					1.000
0	110 (97.3%)	112 (96.6%)	Ref.	Ref.	
1	3 (2.65%)	4 (3.45%)	1.29 [0.26;7.15]	0.748	
2-day:					0.722
0	110 (97.3%)	111 (95.7%)	Ref.	Ref.	
1	3 (2.65%)	5 (4.31%)	1.62 [0.37;8.55]	0.524	
7-day:					0.333
0	110 (97.3%)	109 (94.0%)	Ref.	Ref.	
1	3 (2.65%)	7 (6.03%)	2.28 [0.60;11.4]	0.233	
30-day:					0.060
0	110 (97.3%)	105 (90.5%)	Ref.	Ref.	
1	3 (2.65%)	11 (9.48%)	3.69 [1.10;17.5]	0.034	
90-day:					0.149
0	105 (92.9%)	100 (86.2%)	Ref.	Ref.	
1	8 (7.08%)	16 (13.8%)	2.07 [0.86;5.38]	0.103	
180-day:					0.092
0	103 (91.2%)	96 (82.8%)	Ref.	Ref.	
1	10 (8.85%)	20 (17.2%)	2.12 [0.96;4.99]	0.063	
365-day:					0.015
0	102 (90.3%)	90 (77.6%)	Ref.	Ref.	
1	11 (9.73%)	26 (22.4%)	2.65 [1.26;5.91]	0.010	

0: survival; 1: non-survival

Supplementary Table S4: Summary descriptives table by groups of Hypertensive Emergency

	**Not Suffering Hypertensive Emergency** *N=33*	**Suffering Hypertensive Emergency** *N=29*	**OR**	**p.ratio**	**p.overall**
1-day: 0	33 (100%)	29 (100%)	Ref.	Ref.	.
2-day:					1.000
0	32 (97.0%)	29 (100%)	Ref.	Ref.	
1	1 (3.03%)	0 (0.00%)	. [.;.]	.	
7-day:					1.000
0	32 (97.0%)	29 (100%)	Ref.	Ref.	
1	1 (3.03%)	0 (0.00%)	. [.;.]	.	
30-day:					1.000
0	32 (97.0%)	29 (100%)	Ref.	Ref.	
1	1 (3.03%)	0 (0.00%)	. [.;.]	.	
90-day:					1.000
0	32 (97.0%)	29 (100%)	Ref.	Ref.	
1	1 (3.03%)	0 (0.00%)	. [.;.]	.	
180-day:					0.241
0	30 (90.9%)	29 (100%)	Ref.	Ref.	
1	3 (9.09%)	0 (0.00%)	. [.;.]	.	
365-day:					1.000
0	30 (90.9%)	27 (93.1%)	Ref.	Ref.	
1	3 (9.09%)	2 (6.90%)	0.76 [0.08;5.36]	0.782	

0: survival; 1: non-survival

del Pozo Vegas C, *et al. BMJ Open* 2023; 13:e078815. doi: 10.1136/bmjopen-2023-078815

Supplementary Table S5: Summary descriptives table by groups of ischemic heart disease

	**Not suffering ischemic heart disease** *N=425*	**Suffering ischemic heart disease** *N=264*	**OR**	**p.ratio**	**p.overall**
1-day:					1.000
0	413 (97.2%)	257 (97.3%)	Ref.	Ref.	
1	12 (2.82%)	7 (2.65%)	0.95 [0.34;2.41]	0.910	
2-day:					1.000
0	411 (96.7%)	255 (96.6%)	Ref.	Ref.	
1	14 (3.29%)	9 (3.41%)	1.04 [0.42;2.43]	0.925	
7-day:					0.360
0	401 (94.4%)	254 (96.2%)	Ref.	Ref.	
1	24 (5.65%)	10 (3.79%)	0.66 [0.30;1.38]	0.280	
30-day:					0.994
0	399 (93.9%)	247 (93.6%)	Ref.	Ref.	
1	26 (6.12%)	17 (6.44%)	1.06 [0.55;1.98]	0.859	
90-day:					0.902
0	392 (92.2%)	242 (91.7%)	Ref.	Ref.	
1	33 (7.76%)	22 (8.33%)	1.08 [0.61;1.89]	0.785	
180-day:					0.922
0	387 (91.1%)	239 (90.5%)	Ref.	Ref.	
1	38 (8.94%)	25 (9.47%)	1.07 [0.62;1.81]	0.811	
365-day:					0.254
0	382 (89.9%)	229 (86.7%)	Ref.	Ref.	
1	43 (10.1%)	35 (13.3%)	1.36 [0.84;2.18]	0.211	

0: survival; 1: non-survival

del Pozo Vegas C, *et al. BMJ Open* 2023; 13:e078815. doi: 10.1136/bmjopen-2023-078815

Supplementary Table S6: Summary descriptives table by groups of syncope

	Not suffering syncope<br>*N=221*	Suffering syncope<br>*N=258*	OR	p.ratio	p.overall
1-day:					0.065
0	221 (100%)	253 (98.1%)	Ref.	Ref.	
1	0 (0.00%)	5 (1.94%)	. [.;.]	.	
2-day:					0.004
0	221 (100%)	249 (96.5%)	Ref.	Ref.	
1	0 (0.00%)	9 (3.49%)	. [.;.]	.	
7-day:					0.018
0	220 (99.5%)	247 (95.7%)	Ref.	Ref.	
1	1 (0.45%)	11 (4.26%)	8.65 [1.65;214]	0.007	
30-day:					0.002
0	219 (99.1%)	240 (93.0%)	Ref.	Ref.	
1	2 (0.90%)	18 (6.98%)	7.67 [2.16;52.7]	0.001	
90-day:					<0.001
0	214 (96.8%)	226 (87.6%)	Ref.	Ref.	
1	7 (3.17%)	32 (12.4%)	4.24 [1.93;10.8]	<0.001	
180-day:					<0.001
0	211 (95.5%)	218 (84.5%)	Ref.	Ref.	
1	10 (4.52%)	40 (15.5%)	3.82 [1.93;8.31]	<0.001	
365-day:					<0.001
0	209 (94.6%)	207 (80.2%)	Ref.	Ref.	
1	12 (5.43%)	51 (19.8%)	4.24 [2.26;8.58]	<0.001	

0: survival; 1: non-survival

del Pozo Vegas C, *et al. BMJ Open* 2023; 13:e078815. doi: 10.1136/bmjopen-2023-078815

Supplementary Table S7: Log-regression table by mortality groups

	Survival	Non-Survival	Hazard Ratio [95% Confidence interval]	p.value
	N=1414	***N=330***		
Cohort:				
≤ 74 years	766 (54.2%)	101 (30.6%)	Ref.	Ref.
≥ 75 years	648 (45.8%)	229 (69.4%)	2.41 [1.91;3.04]	<0.001
Sex:				
Male	805 (56.9%)	214 (64.8%)	Ref.	Ref.
Female	609 (43.1%)	116 (35.2%)	0.74 [0.59;0.93]	0.010
Nursing home				
No	1297 (91.7%)	257 (77.9%)	Ref.	Ref.
Yes	117 (8.27%)	73 (22.1%)	2.69 [2.07;3.49]	<0.001
Respiratory Rate	17.9 (6.35)	22.1 (10.5)	1.06 [1.05;1.07]	<0.001
SpO2	95.7 (5.23)	87.2 (13.0)	0.94 [0.93;0.94]	<0.001
SBP	138 (32.7)	123 (40.8)	0.99 [0.98;0.99]	<0.001
DBP	79.0 (19.1)	69.6 (24.5)	0.98 [0.97;0.98]	<0.001
Heart rate	83.5 (31.8)	95.1 (42.9)	1.01 [1.01;1.01]	<0.001
Temperature	36.1 (0.68)	36.1 (0.98)	0.94 [0.81;1.10]	0.424
GCS	14.8 (1.25)	12.8 (4.00)	0.79 [0.77;0.81]	<0.001
Glucose	141 (57.3)	191 (90.8)	1.01 [1.00;1.01]	<0.001
Lactate	1.99 (1.35)	4.63 (3.79)	1.37 [1.33;1.40]	<0.001
NIMV:				
No	1387 (98.1%)	270 (81.8%)	Ref.	Ref.
Yes	27 (1.91%)	60 (18.2%)	6.85 [5.17;9.08]	<0.001
Mechanical ventilation:				
No	1404 (99.3%)	278 (84.2%)	Ref.	Ref.
Yes	10 (0.71%)	52 (15.8%)	14.5 [10.7;19.6]	<0.001
Transcutaneous pacemaker:				

	**Survival**	**Non-Survival**	**Hazard Ratio [95% Confidence interval]**	**p.value**
	N=1414	***N=330***		
No	1364 (96.5%)	320 (97.0%)	Ref.	Ref.
Yes	50 (3.54%)	10 (3.03%)	0.91 [0.48;1.70]	0.759
Cardioversion:				
No	1396 (98.7%)	315 (95.5%)	Ref.	Ref.
Yes	18 (1.27%)	15 (4.55%)	3.24 [1.93;5.43]	<0.001
Defibrillation.:				
No	1406 (99.4%)	306 (92.7%)	Ref.	Ref.
Yes	8 (0.57%)	24 (7.27%)	9.04 [5.95;13.7]	<0.001
Vasoactive agents:				
No	1402 (99.2%)	282 (85.5%)	Ref.	Ref.
Yes	12 (0.85%)	48 (14.5%)	13.4 [9.81;18.2]	<0.001
Prehospital syndromic suspect:				
Ischemic heart disease	611 (43.2%)	78 (23.6%)	Ref.	Ref.
Acute heart failure	138 (9.76%)	147 (44.5%)	6.01 [4.57;7.92]	<0.001
Arrhythmia	192 (13.6%)	37 (11.2%)	1.46 [0.98;2.15]	0.060
Syncope	416 (29.4%)	63 (19.1%)	1.16 [0.83;1.61]	0.394
Hypertensive emergency	57 (4.03%)	5 (1.52%)	0.68 [0.28;1.69]	0.412
ACCI	2.31 (2.33)	4.29 (2.96)	1.23 [1.19;1.27]	<0.001

References

1. Vandenbroucke JP, von Elm E, Altman DG, Gøtzsche PC, Mulrow CD, Pocock SJ, Poole C, Schlesselman JJ, Egger M; STROBE Initiative. Strengthening the Reporting of Observational Studies in Epidemiology (STROBE): explanation and elaboration. PLoS Med. 2007 Oct 16;4(10):e297.
