# Supplementary material for: Prehospital acute life-threatening cardiovascular disease in elderly: an observational, prospective, multicentre, ambulance-based cohort study
Source: BMJ Open. 2023 Nov 23;13(11):e078815. doi: 10.1136/bmjopen-2023-078815 (PMC10668192; doi:10.1136/bmjopen-2023-078815)
Supplement: Author's manuscript [file bmjopen-2023-078815.draft_revisions.pdf]

BMJ Open is committed to open peer review. As part of this commitment we make the peer review history of every article we publish publicly available.

When an article is published we post the peer reviewers' comments and the authors' responses online. We also post the versions of the paper that were used during peer review. These are the versions that the peer review comments apply to.

The versions of the paper that follow are the versions that were submitted during the peer review process. They are not the versions of record or the final published versions. They should not be cited or distributed as the published version of this manuscript.

BMJ Open is an open access journal and the full, final, typeset and author-corrected version of record of the manuscript is available on our site with no access controls, subscription charges or pay-per-view fees (http://bmjopen.bmj.com).

If you have any questions on BMJ Open's open peer review process please email info.bmjopen@bmj.com

BMJ Open

Prehospital acute life-threatening cardiovascular disease in elderly: short- and long-term outcome based on-scene vital signs, and comorbidities.

Journal:	*BMJ Open*
Manuscript ID	bmjopen-2023-078815
Article Type:	Original research
Date Submitted by the Author:	12-Aug-2023
Complete List of Authors:	del Pozo Vegas, Carlos; Hospital Clinico Universitario de Valladolid, Emergency Department; Universidad de Valladolid<br>Zalama-Sánchez, Daniel; Hospital Clinico Universitario de Valladolid, Emergency Department<br>Sanz-Garcia, Ancor; University of Castilla-La Mancha - Center for University Studies Talavera de la Reina<br>López-Izquierdo, Raúl; Hosp Univ Rio Hortega<br>Sáez-Belloso, Silvia; Universidad de Valladolid<br>Oleaga, Cristina Mazas Perez; Universidad Europea del Atlántico; Universidad Internacional Iberoamericana<br>Azpíroz, Irma Domínguez; Universidad Europea del Atlántico; Universidad Internacional Iberoamericana<br>Pascual, Iñaki Elío; Universidad Europea del Atlántico; Universidade Internacional do Cuanza Cuito<br>Martín-Rodríguez, Francisco; Universidad de Valladolid
Keywords:	ACCIDENT & EMERGENCY MEDICINE, Aged, 80 and over, CARDIOLOGY

SCHOLARONE™
Manuscripts

I, the Submitting Author has the right to grant and does grant on behalf of all authors of the Work (as defined in the below author licence), an exclusive licence and/or a non-exclusive licence for contributions from authors who are: i) UK Crown employees; ii) where BMJ has agreed a CC-BY licence shall apply, and/or iii) in accordance with the terms applicable for US Federal Government officers or employees acting as part of their official duties; on a worldwide, perpetual, irrevocable, royalty-free basis to BMJ Publishing Group Ltd ("BMJ") its licensees and where the relevant Journal is co-owned by BMJ to the co-owners of the Journal, to publish the Work in this journal and any other BMJ products and to exploit all rights, as set out in our licence.

The Submitting Author accepts and understands that any supply made under these terms is made by BMJ to the Submitting Author unless you are acting as an employee on behalf of your employer or a postgraduate student of an affiliated institution which is paying any applicable article publishing charge ("APC") for Open Access articles. Where the Submitting Author wishes to make the Work available on an Open Access basis (and intends to pay the relevant APC), the terms of reuse of such Open Access shall be governed by a Creative Commons licence – details of these licences and which Creative Commons licence will apply to this Work are set out in our licence referred to above.

Other than as permitted in any relevant BMJ Author's Self Archiving Policies, I confirm this Work has not been accepted for publication elsewhere, is not being considered for publication elsewhere and does not duplicate material already published. I confirm all authors consent to publication of this Work and authorise the granting of this licence.

For peer review only

Prehospital acute life-threatening cardiovascular disease in elderly: short- and long-term outcome based on-scene vital signs, and comorbidities.

Carlos del Pozo Vegas[1,2], Daniel Zalama-Sánchez[1], Ancor Sanz-García[3,*], Raúl López-Izquierdo[2,4,5], Silvia Sáez-Belloso [6,7], Cristina Mazas Pérez-Oleaga[8,9,10]; Irma Domínguez Azpíroz[8,11,12]; Iñaki Elío Pascual[8,12,13]; Francisco Martín-Rodríguez[2,6]

[1] Emergency Department. Hospital Clínico Universitario. Valladolid, Spain.

[2] Faculty of Medicine. Universidad de Valladolid, Valladolid, Spain.

[3] Faculty of Health Sciences, Universidad de Castilla la Mancha, Talavera de la Reina, Spain.

[4] Emergency Department. Hospital Universitario Rio Hortega. Valladolid, Spain

[5] CIBER of Respiratory Diseases, Instituto de Salud Carlos III, C/Sinesio Delgado, 4 28029, Madrid, Spain.

[6] Advanced Life Support, Emergency Medical Services (SACYL), Valladolid, Spain.

[7] Faculty of Nursing. Universidad de Valladolid, Valladolid, Spain.

[8] Universidad Europea del Atlántico. Isabel Torres 21 39011 Santander, Spain.

[9] Universidad Internacional Iberoamericana. Arecibo, Puerto Rico 00613, USA.

[10] Universidad de La Romana. La Romana, República Dominicana

[11] Universidad Internacional Iberoamericana. Campeche 24560, México.

[12] Universidade Internacional do Cuanza. Cuito, Bié, Angola

[13] Fundación Universitaria Internacional de Colombia. Bogotá, Colombia

* *Corresponding author*

Corresponding author:

Dr. Ancor Sanz-García
Faculty of Health Sciences, Universidad de Castilla la Mancha. Avda. Real Fábrica de Seda, s/n 45600, Talavera de la Reina, Toledo
e-mail: ancor.sanz@gmail.com
Phone: 925 721 010

Authors' Contributions

CRediT authorship contribution statement:

Francisco Martín-Rodríguez conceptualized the project, managed and coordinated the project, assisted with the design of methodology, analyzed data, and prepared the initial and final drafts of

For peer review only - http://bmjopen.bmj.com/site/about/guidelines.xhtml

the manuscript. Ancor Sanz-García took responsibility for the data and their analysis. Carlos del Pozo Vegas, Daniel Zalama-Sánchez, Silvia Sáez-Belloso, and Raúl López Izquierdo assisted with management and coordination of the project, assisted with design of methodology, and helped review the manuscript. All authors performed a critical review and approval of the final manuscript for interpretation of the data and important intellectual input.

Summarized conflict of interest statements

All signing authors meet the requirements of authorship and have declared the nonexistence of potential conflicts of interest. Daniel Zalama-Sánchez, Ancor Sanz-García, Carlos del Pozo Vegas, Raúl López-Izquierdo, Silvia Sáez-Belloso, and Francisco Martín-Rodríguez report no conflicts of interest.

The authors have no disclosures to make.

On behalf of the other authors, the corresponding author guarantees the accuracy, transparency, and honesty of the data and information contained in the study, that no relevant information has been omitted and that all discrepancies between authors have been adequately resolved and described.

Funding information

This work was supported by the Gerencia Regional de Salud, Public Health System of Castilla y León (Spain) [grant number GRS 1903/A/19 and GRS 2131/A/20].

Sponsor’s role: none

Ethics approval

This study was approved by the Health Research Ethics Board from all participating centers. The study is registered in the WHO International Clinical Trials Registry Platform (ICTRP) with number [ISRCTN48326533 and ISRCTN49321933]. Details of the study design, statistical analysis plan and raw data are available online.

Notation of prior abstract publication/presentation

This article is an original work, has not been published before and is not being considered for publication elsewhere in its final form, in either printed or electronic media. It is not based on any previous communication to a society or meeting.

Data Availability

The data that support the findings of this study are available on request from the corresponding author ASG. The data are not publicly available due to restrictions, their containing information that could compromise the privacy of research participants.

Running head

Prehospital cardiovascular disease in elderly

For peer review only

For peer review only - http://bmjopen.bmj.com/site/about/guidelines.xhtml

Abstract

Background

The aim was to explore the association of demographic and prehospital parameters with short- and long-term mortality in acute life-threatening cardiovascular disease by using a hazard model, focusing on elderly individuals, by comparing patients under 75 years versus patients over 75 years of age.

Methods

This was a prospective, multicenter, observational, EMS-delivery study gathering data from two back-to-back studies between October 1, 2019, and November 30, 2021. Six advanced life support (ALS), forty-three basic life support (BLS), and five hospitals in Spain were considered. The primary outcome was in-hospital mortality from any cause within the first to the 365 day following EMS attendance. The main measures included prehospital demographics, biochemical variables, prehospital advanced life support techniques used, and syndromic suspected conditions.

Results

A total of 1744 patients fulfilled the inclusion criteria. The 365-day cumulative mortality in the elderly amounted to 26.1% (229 cases) vs 11.6% (11.6%) in patients under 75 years old. Elderly patients (≥ 75 years) presented a 2-fold risk of mortality compared to patients ≤ 74 years. Life-threatening interventions (mechanical ventilation, cardioversion and defibrillation) were also related to a 2-fold increased risk of mortality. Importantly, patients suffering from acute heart failure presented a more than 2-fold increased risk of mortality.

Conclusions

This study revealed the prehospital variables associated with the long-term mortality of patients suffering from acute cardiovascular disease. Our results provide important insights for the development of specific codes or scores for cardiovascular diseases to facilitate the risk of mortality characterization.

Keywords Prehospital; Emergency Medical Services; elderly; cardiovascular disease; mortality.

Key messages

What is already known on this topic: Elderly is a critical factor for prognosis

What this study adds: How this elderly affects cardiovascular patients treated by emergency medicine services is unknown

How this study might affect research, practice or policy: This study revealed the prehospital variables associated with the long-term mortality of patients suffering from acute cardiovascular disease.

For peer review only - http://bmjopen.bmj.com/site/about/guidelines.xhtml

Introduction

Cardiovascular diseases represent the leading cause of prehospital care, involving a surprising number of unplanned hospitalizations and sudden unexplained mortality (1). Emergency medical services (EMS) must handle this overwhelming patient workload quickly and efficiently, following clinical guidelines and under recognized training, e.g., basic and advanced cardiac life support (BCLS and ACLS) (2) (3).

The setup and implementation of specific detection codes for life-threatening conditions, e.g., cardiorespiratory arrest, ST-elevation coronary syndrome, and stroke, is a well-established procedure in health systems, and EMS plays an active role in detection, emergency critical care, and assisted transfer to a suitable hospital (4). Predefined standard protocols should be applied in diseases with clear symptomatology or well-defined guiding symptoms, and in general, no major operational problems are encountered. The handicap for EMS providers consists of the identification on-scene or *en* route of patients with acute life-threatening cardiovascular disease that is apparently masked. Early warning scores and point-of-care testing can provide an effective support tool to help at critical junctures in the complicated decision-making process (5) (6). However, the identification of high-risk subjects is challenging, particularly when distracting factors such as comorbidities or age come into play (7).

EMS has increasingly switched types of patients, with older adults becoming a major focus area of care (8). Among other reasons, falls, drug-taking mistakes, and exacerbations of chronic pathologies are more frequent in elderly individuals, i.e., EMS providers can attend to cases of older adults with comorbidities and multimedication. In particular, cardiovascular diseases constitute the first cause of emergency appointments and inpatient hospitalization in older adults, affecting both males and females (9). Life expectancy has increased significantly, meaning that older adults with atrial fibrillation, acute coronary syndrome, congestive heart failure, valvular heart disease, and other cardiovascular processes are becoming increasingly prevalent (10). Complications related to aging can hamper anamnesis and clinical examination and sometimes disrupt responsiveness mechanisms, e.g., 20% of older adults exhibit atypical symptoms of acute coronary syndrome (11). Additionally, coexisting comorbidities may trigger interactions in the cardiovascular system, such as anemia, chronic kidney disease, or diabetes (12). Comorbidities naturally rise sharply with age and necessitate an appropriate analysis.

The purpose of the study was to explore the association of demographic and prehospital parameters with short- and long-term mortality in acute life-threatening cardiovascular disease by using a hazard model. In particular, we focused on the elderly by comparing two age cohorts: under 75 years versus over 75 years.

For peer review only - http://bmjopen.bmj.com/site/about/guidelines.xhtml

Methods

Study design

This prospective, multicenter, observational, EMS-delivery, analysis gathers inputs obtained from two back-to-back studies, "Prehospital identification of prognostic biomarkers in time-dependent diseases" (HITScore) and "Identification of biomarkers of clinical-risk deterioration in prehospital care" (preBIOs).

Ethical approval was granted by the Rio Hortega University Hospital Ethics Committee, references PI041-19 and PI217-20, the trial registered on the ISRCTN database (ISRCTN48326533 and ISRCTN49321933), according to STrengthening the Reporting of OBservational studies in Epidemiology (Supplementary data p3) (13), and conducted in accordance with the Declaration of Helsinki.

Study setting

The study was conducted in four Spanish provinces (Burgos, Salamanca, Segovia, and Valladolid), enrolling uninterrupted adults (>18 years) with a syndromic cardiovascular suspect who were transferred by ambulance to the Emergency Department (ED) between October 1, 2019, and November 30, 2021. Global community medical care was provided by the Public Health System (SACYL) and included the Emergency Coordination Center (1-1-2 phone backup), six advanced life support (ALS), forty-three basic life support (BLS), and five hospitals (one minor general district hospital and four university tertiary hospitals). Normally, BLS is staffed by two emergency medical technicians (EMTs), performing on-scene or *en route* BCLS work-up protocols, and ALS is made up of two EMTs, an emergency registered nurse (ERN) and a physician, conducting ACLS operations.

No exception, all cases were examined by an ALS, and following the assessment and diagnostic tests, the physician determined, in line with current guidelines and according to the individual clinical situation, the need for transfer to the ED, as well as the type of ambulance: BLS or ALS. All hospitals presented the Acute Cardiac Care Unit (ACCU), and three hospitals presented the cardiac intervention room 24/7 and emergency cardiac surgery unit. Patients who required emergency hemodynamic studies or advanced cardiologic care, and these facilities were not available at the reference hospital, were evacuated as top priority (daytime by Helicopter Emergency Medical Service, and nighttime by ALS, mandatory with turnaround times under 1 hour) to other hospitals included in the study.

Population

Recruitment was consecutive. Participants enrolled in the study were defined as adults (>18 years) with acute cardiovascular disease (prehospital syndromic suspected condition) managed by EMS and transferred to the ED. Noncardiovascular disorders, minors, pregnant women (known or apparent), terminally ill patients (condition confirmed by a medical report), or on-site discharge were excluded.

For peer review only

An informed consent form, managed by the ERN and applicable for the entire follow-up study, was reviewed and countersigned by all participants. In the absence of appropriate understanding by the patient, a research associate in the ED tried to collect the consent form signed by the patient or by a family member or legal guardian. If, despite all efforts, consent was not obtained, the patient was removed and excluded from the study.

Data collection

Mandatory on-site hands-on training was conducted for all staff before the study started and included the standardized procedure for taking vital signs, handling, calibration, and cleaning of the point-of-care testing device, as well as data input to a database specially created for this purpose.

Age, sex, nursing home location, and on-scene vital signs (respiratory rate, oxygen saturation, systolic and diastolic blood pressure, heart rate, temperature, Glasgow Coma Scale, glucose, lactate, and electrocardiogram) were collected and recorded by the ERN. A LifePAK® 15 monitor-defibrillator (Physio-Control, Inc., Redmond, USA) was applied to obtain oxygen saturation, blood pressure, heart rate and electrocardiogram. A ThermoScan® PRO 6000 thermometer (Welch Allyn, Inc, Skaneateles Falls, USA) was used to collect temperature, and finally, the analyzer epoc® (Siemens Healthcare GmbH, Erlangen Germany) was employed to perform prehospital analysis.

The physician subsequently checked the electrocardiogram and recorded the baseline heart rhythm, as well as the 17 comorbidities categories required to calculate the Age-Charlson comorbidity index (ACCI), composed of myocardial infarction, congestive heart failure, peripheral vascular disease, stroke or transient ischemic attack, dementia, chronic obstructive pulmonary disease, connective tissue disease peptic ulcer disease, mild liver disease, uncomplicated diabetes mellitus, hemiplegia, moderate to severe chronic kidney disease, diabetes mellitus with end-organ damage, localized solid tumor, leukemia, lymphoma, moderate to severe liver disease, metastatic solid tumor, and acquired immunodeficiency syndrome.

Outcomes

The primary outcome was cumulative mortality, from prehospital care to one year of follow-up, segregating time periods as follows: 1, 2, 7, 30, 90, 180, and 365 days. In addition, a comparison was performed matching two age-typed cohorts, a group of ≤ 74 years vs a group of ≥ 75 years, with age discrimination in line with similar reports (14) (15).

The secondary prehospital outcomes included advanced airway management (noninvasive or invasive mechanical ventilation), electrical therapy (transcutaneous pacemaker, cardioversion, or defibrillation) and/or vasoactive agents. Finally, the ALS physician appointed the prehospital syndromic suspected condition, involving ischemic heart disease, acute heart failure, arrhythmia, syncope, or hypertensive emergency.

For peer review only

The secondary hospital outcomes were collected from the electronic medical records obtained at the one-year follow-up and comprised cumulative mortality (all-cause), admission rate, echocardisocopy, percutaneous interventional vascular surgery, emergent surgery, advanced airway management, vasoactive agents, and ACCU admission.

Data analyses

Descriptive results and the associations between age and cardiovascular diagnosis with the variables were assessed by the Mann–Whitney U test or the chi-squared test, when appropriate, and the effect size in the form of standardized mean difference was provided. Absolute values and percentages were used for categorical variables, and median interquartile ranges (IQR) were used for continuous variables because they did not follow a normal distribution. A Cox regression was performed to evaluate the association of demographic and prehospital parameters (those with $p<0.001$ in the log-rank univariate analysis) with mortality. Furthermore, survival according to age, cardiovascular diagnosis, and the combination of both variables was obtained using the Kaplan–Meier method (KM). Data for prehospital covariates were prospectively collected and registered in a database generated with IBM SPSS Statistics for Apple version 20.0 software. (IBM Corp, Armonk USA). The caseload entry system was test-run to delete unclear or ambiguous items and to verify the adequacy of the data gathering system. The data present missing values completely at random; therefore, the strategy used (listwise deletion) does not imply biased means, variances or regression weights. The statistical power (from 1 to 100) of the present study is 89.4 based on the following considerations: (i) the sample used is n = 517, (ii) significant level of p = 0.05, (iii) expected odds ratios (OR) of 0.405, and (iv) 14% of events.

For peer review only - http://bmjopen.bmj.com/site/about/guidelines.xhtml

Results

Among 2409 cases, 1744 patients with a syndromic cardiovascular suspect managed by EMS and transferred to the ED were finally included in the final analysis. There were 867 patients from cohort #1 (≤ 74 years) and 877 from cohort #2 (≥ 75 years), 49.7% vs 50.3% (figure 1).

Cohort #1 was characterized by a median age of 62 years (IQR: 52-69), and 33.1% (287 cases) were females. Half of the cases (56.2%) showed sinus rhythm, and the comorbidity burden resulted in an ACCI of 1 point (IQR: 0-3), especially myocardial infarction (27.3%), chronic pulmonary disease (21.8%) and nonmetastatic solid tumor (13.7%). In contrast, cohort #2 was described by a median age of 84 years (IQR: 79-88), 49.9% (438 cases) were female, and one-fifth of nursing homes. Despite sinus rhythm being the most common baseline cardiac rhythm (38.5%), the ratio of atrial fibrillation (33.4%) is noteworthy. The ACCI score was significantly higher, with a median of 3 points (IQR: 2-5) and a very pronounced prevalence of myocardial infarction (36.6%), congestive heart failure (31.8%), and nonmetastatic solid tumor (23.8%) (table 1).

The 365-day cumulative mortality in the elderly amounted to 26.1% (229 cases) vs 11.6% (11.6%) in patients under 75 years old. Cohort #1 presented a significantly increased percentage of mechanical ventilation, defibrillation, cardioversion, percutaneous interventional vascular surgery, and ACCU admission vs cohort #2, with a relatively increased incidence of noninvasive mechanical ventilation and transcutaneous pacemakers. Concerning prehospital syndromic suspected conditions, patients under 75 years old presented half with ischemic heart disease (49%), followed by syncope (25.2%). The elderly also showed ischemic heart disease (30.1%) and syncope (29.4%), stressing in particular the elevated incidence of acute heart failure (23.9% vs. 8.8%, respectively intercohort) (table 2). Cumulative mortality by prehospital syndromic suspected condition is reported in Supplementary Table S1, and the OR of each prehospital syndromic suspected condition for each mortality interval can be found in Supplementary Tables S2 to S6.

The Cox regression (Table 3) that included all those variables with p<0.001 in the long-rank analysis (supplementary table S7) showed that being elderly, being in a nursing home, high respiratory rate, low systolic blood pressure, high levels of lactate, use of noninvasive mechanical ventilation, mechanical ventilation, cardioversion defibrillation, suffering from acute heart failure and an elevated Age-Charlson comorbidity index were variables statistically significantly associated with mortality. The aforementioned results for elderly individuals and those with acute heart failure are illustrated in Figure 2. Figure 2A shows the survival curve of patients ≤ 74 years vs ≥ 75 years, with statistically significant differences (p<0.001). Figure 2B shows the mortality curves for each prehospital syndromic suspected condition. Acute heart failure presented the highest mortality. When analyzing the results according to age, those patients suffering from arrhythmia and syncope presented statistically significant differences in the mortality curves (Supplementary Figure S1).

For peer review only - http://bmjopen.bmj.com/site/about/guidelines.xhtml

For peer review only

Discussion

This prospective, multicenter, observational, EMS-delivery study assessed the association of prehospital variables, particularly elderly variables, with short- and long-term mortality in patients with acute life-threatening cardiovascular diseases. Elderly patients (≥ 75 years) presented a 2-fold risk of mortality compared to patients ≤ 74 years. Life-threatening interventions (mechanical ventilation, cardioversion and defibrillation) were also related to a 2-fold increased risk of mortality. Importantly, patients suffering from acute heart failure presented a more than 2-fold increased risk of mortality.

Cardiovascular diseases are the leading cause of EMS attendance (1) and are associated with high morbidity and mortality (16). Therefore, the accurate and quick characterization of patients at the first contact could provide critical information for the development of specialized risk scores (17) or specific detection codes (4). In this sense, the present study aims to describe the association of prehospital variables with mortality, particularly with elderly individuals, which is one of the main risk factors for cardiovascular diseases (18). Our results align with this evidence, since the categorization of patients ≤ 74 years and ≥ 75 years revealed that older patients presented a higher rate of mortality. Moreover, other factors related to elderly individuals, such as being in nursing homes or the number of comorbidities, measured by the ACCI, were risk factors for mortality. As expected, the higher the number of comorbidities, the higher the risk of mortality. This is not surprising since several comorbidities worsen cardiovascular conditions (12). In fact, comorbidities make patient examination difficult, which is particularly true in the prehospital setting (19).

Despite all this evidence, the effect of age on mortality was not found for short-term mortality, and no statistically significant effect was found for one- or two-day mortality; instead, the statistical significance was relevant at 30 days onward. This short-term mortality result is somewhat surprising, since it has been previously described at the prehospital level that higher ages are associated with higher short-term mortality (20). Perhaps this difference could be explained by the fact that those patients were characterized as suffering mainly from respiratory diseases (20). The long-term mortality relationship with age has already been described in the prehospital setting (21).

Lactate was statistically related to long-term mortality; this has also been reported, as for respiratory rate and age, for long-term mortality when dealing with all the patients and not with disease-specific analysis (21). Enriquez de Salamanca Gambara et al. (21) also found that GCS and SpO_2 were associated with mortality. Perhaps GCS was more related to neurological diseases than cardiovascular diseases, which is our case. A similar argument could be made for SpO_2, which could be related to respiratory diseases.

For peer review only - http://bmjopen.bmj.com/site/about/guidelines.xhtml

Life-threatening interventions are closely related to patients suffering from cardiovascular disease (22), which was also our case, since noninvasive mechanical ventilation, mechanical ventilation, cardioversion, and defibrillation were procedures present in the nonsurvivor group.

Particularly relevant is the fact that the mortality of each prehospital syndromic suspected condition was different when considering elderly individuals. As expected, acute heart failure was the main condition related to death; however, when analyzing mortality according to age groups, only arrhythmia and syncope presented statistically significant differences. This should be interpreted as the higher the age, the higher the probability of death, but only for those two conditions. The other conditions should be treated independently of age. The results for syncope and arrhythmia could be explained by the increased incidence with age, particularly at 70 years for syncope (23) and arrythmias, especially for atrial fibrillation (24).

Our study has some strengths, including the sample size, novelty, and study design with a reduced loss-to-follow-up rate. It was conducted in both rural and urban areas, and our results could be generalizable to other health systems. This last point is based on the fact that all parameters associated with mortality can be easily accessed by the EMS staff. Only lactate determination will require a point-of-care device, which is now a reality in several EMSs (25). This generalizability could lead to scores or specific detection codes. However, some limitations must be considered. First, this is an observational study, so we cannot rule out the possibility of selection bias, although participating centers had previous experience and enrolled consecutive patients with prehospital acute cardiovascular disease. To obtain a representative sampling, cases were collected 24/7/365 nonstop in urban, semiurban and rural areas and in different ambulance stations. Second, possible bias may exist in relation to case inclusion in the study. All prehospital acute cardiovascular conditions were included; however, certain disorders may raise uncertainty, i.e., stomachache could be labeled as a digestive disease at prehospital care, although following complementary in-hospital tests (imaging studies, laboratory tests, etc.), the disease ends up being categorized as ischemic heart disease. Nevertheless, the limitation was dampened for two reasons. On-scene, ALS physicians could issue up to a maximum of three diagnostic suspicions, and therefore, any case with a diagnosis of acute cardiovascular disease was eventually included in the analysis. In addition, the diagnostic coincidence between prehospital syndromic cardiovascular suspect (ischemic heart disease, acute heart failure, arrhythmia, syncope, and hypertensive emergency) and the final in-hospital diagnosis was very consistently strong. Third, data extractors were not blinded. To avoid cross-contamination, EMS providers lacked access to hospital follow-up data, and vice versa, hospital investigators remained blinded to prehospital care data. To accurately link the data between prehospital care and hospital follow-up, at least 5 of the following extractors had to be an exact match: health system ID card, incident reference (EMS-register), first and last name, age, sex at birth, date, and/or time of arrival at

For peer review only - http://bmjopen.bmj.com/site/about/guidelines.xhtml

the ED. Total access to the master database was given exclusively by the principal investigator and data manager. Finally, an adequate sample size was used in the present preliminary study, but multicenter studies in different health systems are needed to confirm the generalizability of the results.

In summary, this study revealed the prehospital variables associated with the long-term mortality of patients suffering from acute cardiovascular disease. Elderly, life-threatening interventions, acute heart failure, comorbidities, and lactate should be considered when the EMS faces patients with acute life-threatening cardiovascular diseases. In particular, the effect of elderly patients presenting arrhythmia or syncope should be considered. Finally, our results could pave the way to the development of specific codes or scores for cardiovascular diseases to facilitate the risk of mortality characterization.

For peer review only

For peer review only - http://bmjopen.bmj.com/site/about/guidelines.xhtml

References

1. Townsend N, Kazakiewicz D, Lucy Wright F, Timmis A, Huculeci R, Torbica A, et al. Epidemiology of cardiovascular disease in Europe. Nat Rev Cardiol. 2022; 19(2): p. 133-143.
2. Perkins GD, Graesner JT, Semeraro F, Olasveengen T, Soar J, Lott C, et al. European Resuscitation Council Guidelines 2021: Executive summary. Resuscitation. 2021; 161: p. 1-60.
3. Wyckoff MH, Singletary EM, Soar J, Olasveengen TM, Greif R, Liley HG, et al. 2021 International Consensus on Cardiopulmonary Resuscitation and Emergency Cardiovascular Care Science With Treatment Recommendations: Summary From the Basic Life Support; Advanced Life Support; Neonatal Life Support; Education, Implementation, and Teams. Circulation. 2022; 145(9): p. e645-e721.
4. Andersson HB, Seth M, Gurm HS, Bates ER. Pre-hospital Transport Times and Outcomes After Different Reperfusion Strategies for ST-Elevation Myocardial Infarction. Am J Cardiol. 2019; 123(3): p. 375-381.
5. Pirneskoski J, Lääperi M, Kuisma M, Olkkola KT, Nurmi J. Ability of prehospital NEWS to predict 1-day and 7-day mortality is reduced in older adult patients. Emerg Med J. 2021; 38(12): p. 913-918.
6. Martín-Rodríguez F, López-Izquierdo R, Castro Villamor MA, Del Pozo Vegas C, Delgado Benito MDP, Martínez Caballero CM, et al. The Prognostic Value of Prehospital Blood Lactate Levels to Predict Early Mortality in Acute Cardiovascular Disease. Shock. 2020; 53(2): p. 164-170.
7. Forman DE, Maurer MS, Boyd C, Brindis R, Salive ME, Horne FM, et al. Multimorbidity in Older Adults With Cardiovascular Disease. J Am Coll Cardiol. 2018; 71(19): p. 2149-2161.
8. Probst MA, Gibson T, Weiss RE, Yagapen AN, Malveau SE, Adler DH, et al. Risk Stratification of Older Adults Who Present to the Emergency Department With Syncope: The FAINT Score. Ann Emerg Med. 2020; 75(2): p. 147-158.
9. Ijaz N, Buta B, Xue QL, Mohess DT, Bushan A, Tran H, et al. Interventions for Frailty Among Older Adults With Cardiovascular Disease: JACC State-of-the-Art Review. J Am Coll Cardiol. 2022; 79(5): p. 482-503.
10. Triposkiadis F, Xanthopoulos A, Butler J. Cardiovascular Aging and Heart Failure: JACC Review Topic of the Week. J Am Coll Cardiol. 2019; 74(6): p. 804-813.
11. Magidson PD. The Aged Heart. Emerg Med Clin North Am. 2022; 40(4): p. 637-649.
12. Triposkiadis F, Xanthopoulos A, Parissis J, Butler J, Farmakis D. Pathogenesis of chronic heart failure: cardiovascular aging, risk factors, comorbidities, and disease modifiers. Heart Fail Rev. 2022; 27(1): p. 337-344.
13. von Elm E, Altman DG, Egger M, Pocock SJ, Gøtzsche PC, Vandenbroucke JP. The Strengthening the Reporting of Observational Studies in Epidemiology (STROBE) statement: guidelines for reporting observational studies. Lancet. 2007; 370(9596): p. 1453-1457.
14. Duvekot MHC, Venema E, Rozeman AD, Moudrous W, Vermeij FH, Biekart M, et al. Comparison of eight prehospital stroke scales to detect intracranial large-vessel occlusion in suspected stroke (PRESTO): a prospective observational study. Lancet Neurol. 2021; 20(3): p. 213-221.
15. Martín-Rodríguez F, Sanz-García A, Ortega Moreno L, Del Pozo Vegas C, Castro-Villamor MA, Martín-Conty JL, et al. Risk for early death in acutely ill older adults attended by prehospital emergency medical services. Emergencias. 2020; 32(3): p. 117-184
16. Roth G, Mensah G, Johnson C, et al. Global Burden of Cardiovascular Diseases and Risk Factors, 1990–2019. J Am Coll Cardiol. 2020; 76:2982–3021.

For peer review only

17. Than, M.P., Flaws, D.F., Cullen, L. et al. Cardiac Risk Stratification Scoring Systems for Suspected Acute Coronary Syndromes in the Emergency Department. Curr Emerg Hosp Med Rep. 2013; 1:53–63.
18. Wibring K, Herlitz J, Christensson L, Lingman M, Bång A. Prehospital factors associated with an acute life-threatening condition in non-traumatic chest pain patients - A systematic review. Int J Cardiol. 2016; 219:373-9.
19. Lai PH, Lancet EA, Weiden MD, Webber MP, Zeig-Owens R, Hall CB, Prezant DJ. Characteristics Associated With Out-of-Hospital Cardiac Arrests and Resuscitations During the Novel Coronavirus Disease 2019 Pandemic in New York City. JAMA Cardiol. 2020; 5:1154-1163.
20. Martín-Rodríguez F, López-Izquierdo R, Sanz-García A, Del Pozo Vegas C, Ángel Castro Villamor M, Mayo-Iscar A, et al. Novel Prehospital Phenotypes and Outcomes in Adult-Patients with Acute Disease. J Med Syst. 2022; 46:45.
21. Enriquez de Salamanca Gambara R, Sanz-García A, Martín-Conty JL, Polonio-López B, Del Pozo Vegas C, Martín-Rodríguez F, López-Izquierdo R. Long-Term Mortality in Patients Transferred by Emergency Medical Services: Prospective Cohort Study. Prehosp Disaster Med. 2023; 38:352-359.
22. Beygui F, Castren M, Brunetti ND, et al. Pre-hospital management of patients with chest pain and/or dyspnoea of cardiac origin. A position paper of the Acute Cardiovascular Care Association (ACCA) of the ESC. European Heart Journal: Acute Cardiovascular Care. 2020; 9:59-81.
23. Torabi P, Rivasi G, Hamrefors V, Ungar A, Sutton R, Brignole M, Fedorowski A. Early and late-onset syncope: insight into mechanisms. Eur Heart J. 2022; 43:2116-2123.
24. Gómez-Doblas JJ, Muñiz J, Martin JJ, Rodríguez-Roca G, Lobos JM, Awamleh P, et al. Prevalence of atrial fibrillation in Spain. OFRECE study results. Rev Esp Cardiol. 2014; 67:259-69
25. Morris E, McCartney D, Lasserson D, Van den Bruel A, Fisher R, Hayward G. Point-of-care lactate testing for sepsis at presentation to health care: a systematic review of patient outcomes. Br J Gen Pract. 2017 Dec;67(665):e859-e870.

For peer review only

Tables and figures

Table 1. Demographic and clinical baseline variables

Variable	≤ 74 years	≥ 75 years	Standardized difference[b]	Odds ratio[c] (95%CI)	*p* value[d]
No. (%) with data[a]	867 (49.7)	877 (50.3)	N.A.	N.A.	N.A.
**Epidemiological variables**					
Sex, female	287 (33.1)	438 (49.9)	0.168	0.50 [0.41;0.60]	<0.001
Age, year	62 (52-69)	84 (79-88)	2.486	N.A	<0.001
Nursing homes	21 (2.4)	169 (19.3)	0.169	0.10 [0.06;0.16]	<0.001
**On-scene vital signs**					
RR, breaths/min	16 (14-19)	17 (14-23)	0.166	0.98 [0.97;0.99]	0.001
SpO2, %	97 (95-98)	96 (93-98)	-0.225	1.03 [1.02;1.04]	<0.001
SBP, mmHg	135 (112-153)	134 (112-157)	0.043	1.00 [1.00;1.00]	0.366
DBP, mmHg	80 (67-94)	73 (60-86)	-0.301	1.02 [1.01;1.02]	<0.001
HR, beats/min	80 (65-100)	78 (62-99)	-0.119	1.00 [1.00;1.01]	0.013
Temperature, °C	36 (35.9-36.5)	36 (35.8-36.5)	0.013	0.98 [0.87;1.11]	0.778
Glasgow coma scale, points	15 (15-15)	15 (15-15)	-0.019	1.01 [0.97;1.05]	0.691
Glucose, mg/dL	124 (105-151)	141 (116-183)	0.266	1.00 [0.99;1.00]	<0.001
Lactate, mmol/L	1.77 (1.1-2.82)	1.97 (1.19-3.1)	0.042	0.98 [0.94;1.02]	0.376
**Baseline cardiac rhythm**					
Sinus	487 (56.2)	338 (38.5)	-0.176	2.04 [1.69;2.47]	<0.001
Atrial fibrillation	127 (14.6)	293 (33.4)	0.187	0.34 [0.27;0.43]	<0.001
Atrial flutter	7 (0.8)	8 (0.9)	0.001	0.89 [0.30;2.52]	0.820
Atrial tachycardia	98 (11.3)	63 (7.2)	-0.041	1.64 [1.18;2.30]	0.003
Supraventricular tachycardia	23 (2.7)	12 (1.4)	-0.012	1.95 [0.98;4.10]	0.058
Ventricular tachycardia	22 (2.5)	4 (0.5)	-0.020	5.50 [2.08;19.3]	<0.001
Sinus bradycardia	62 (7.2)	57 (6.5)	-0.006	1.11 [0.76;1.61]	0.591
1° degree block	3 (0.3)	24 (2.7)	0.023	0.13 [0.03;0.37]	<0.001
2° block type I	2 (0.2)	2 (0.2)	-0.000	1.01 [0.11;9.73]	0.991
2° block type II	4 (0.5)	7 (0.8)	0.003	0.59 [0.15;2.00]	0.397
Complete block	16 (1.8)	24 (2.7)	0.008	0.67 [0.35;1.27]	0.219
Pacemaker	6 (0.7)	40 (4.6)	0.008	0.15 [0.06;0.33]	<0.001
Junctional	1 (0.1)	3 (0.3)	0.038	0.37 [0.01;3.16]	0.380
Idioventricular	3 (0.3)	0	-0.003	N.A	
Asystole	3 (0.3)	0	-0.003	N.A	
Ventricular fibrillation	3 (0.3)	2 (0.2)	-0.001	1.48 [0.23;12.8]	0.678
**Comorbidities**					
ACCI, points	1 (0-3)	3 (2-5)	0.557	0.79 [0.76;0.83]	<0.001
Congestive heart failure	109 (12.6)	279 (31.8)	0.192	0.31 [0.24;0.39]	<0.001
Myocardial infarction	237 (27.3)	321 (36.6)	0.092	0.65 [0.53;0.80]	<0.001
Peripheral vascular disease	97 (11.2)	137 (15.6)	0.044	0.68 [0.51;0.90]	0.007
Cerebrovascular disease	37 (4.3)	120 (13.7)	0.094	0.28 [0.19;0.41]	<0.001
Hemiplegia	23 (2.7)	48 (5.5)	0.028	0.47 [0.28;0.78]	0.003
Chronic pulmonary disease	189 (21.8)	199 (22.7)	0.008	0.95 [0.76;1.19]	0.655
DM uncomplicated	113 (13)	151 (17.2)	0.041	0.72 [0.55;0.94]	0.015
DM end organ damage	73 (8.4)	129 (14.7)	0.062	0.53 [0.39;0.72]	<0.001
Moderate-severe CKD	41 (4.7)	190 (21.7)	0.169	0.18 [0.13;0.25]	<0.001
Mild hepatic disease	30 (3.5)	27 (3.1)	-0.003	1.13 [0.66;1.93]	0.654
Severe hepatic disease	21 (2.4)	16 (1.8)	-0.006	1.33 [0.69;2.62]	0.387
Peptic ulcer disease	75 (8.7)	76 (8.7)	0.002	1.00 [0.71;1.40]	0.991
AIDS	4 (0.5)	1 (0.1)	-0.003	3.68 [0.51;101]	0.175
Lymphoma	7 (0.8)	16 (1.8)	0.010	0.44 [0.17;1.05]	0.063
Leukemia	13 (1.5)	13 (1.5)	-0.002	1.01 [0.46;2.23]	0.977
Metastatic solid tumor	20 (2.3)	33 (3.8)	0.014	0.61 [0.34;1.06]	0.077
Nonmetastatic solid tumor	119 (13.7)	209 (23.8)	0.101	0.51 [0.40;0.65]	<0.001
Connective tissue disease	48 (5.5)	49 (5.6)	0.001	0.99 [0.66;1.49]	0.963
Dementia	27 (3.1)	136 (15.5)	0.123	0.18 [0.11;0.27]	<0.001

Abbreviations: NA: Not applicable; Ref: reference; RR: respiratory rate; SPO2: oxygen saturation; SBP: systolic blood pressure; DBP: diastolic blood pressure; HR: heart rate; ACCI: Age-Charlson comorbidity index; DM: diabetes mellitus; CKP: chronic kidney disease; AIDS: acquired immunodeficiency syndrome.

For peer review only - http://bmjopen.bmj.com/site/about/guidelines.xhtml

[a]Values expressed as total number (percentage) and medians (25th-75th percentile), as appropriate.
[b]The Mann–Whitney U test or chi-squared test was used as appropriate.
cCohen's d-test was used to estimate the effect size.
[d]Fisher's exact probability statistic was used.

For peer review only

For peer review only - http://bmjopen.bmj.com/site/about/guidelines.xhtml

Table 2. Primary and key secondary outcomes

Variable	≤ 74 years	≥ 75 years	Standardized difference[b]	Odds ratio[c] (95%CI)	*p* value[d]
No. (%) with data[a]	867 (49.7)	877 (50.3)	N.A.	N.A.	N.A.
**Cumulative mortality**					
1-day	25 (2.9)	35 (4)	0.011	0.72 [0.42;1.20]	0.205
2-day	30 (3.5)	51 (5.8)	0.023	0.58 [0.36;0.92]	0.019
7-day	42 (4.8)	69 (7.9)	0.030	0.60 [0.40;0.88]	0.010
30-day	53 (6.1)	106 (12.1)	0.059	0.47 [0.33;0.67]	<0.001
90-day	74 (8.5)	156 (17.8)	0.092	0.43 [0.32;0.58]	<0.001
180-day	88 (10.1)	184 (21)	0.108	0.43 [0.32;0.56]	<0.001
365-day	101 (11.6)	229 (26.1)	0.144	0.37 [0.29;0.48]	<0.001
**Secondary outcome (support on-scene)**					
NIMV	22 (2.5)	65 (7.4)	0.048	0.33 [0.20;0.53]	<0.001
Mechanical ventilation	39 (4.5)	23 (2.6)	-0.018	1.74 [1.04;2.99]	0.034
Transcutaneous pacemaker	27 (3.1)	33 (3.8)	0.006	0.82 [0.49;1.38]	0.458
Cardioversion	22 (2.5)	11 (1.3)	-0.012	2.03 [1.00;4.41]	0.049
Defibrillation	24 (2.8)	8 (0.9)	-0.018	3.05 [1.41;7.35]	0.004
Vasoactive agents	31 (3.6)	29 (3.3)	-0.002		0.758
**Prehospital syndromic suspected condition**					
Ischemic heart disease	425 (49)	264 (30.1)	-0.189	0.45 [0.37;0.55]	<0.001
Acute heart failure	76 (8.8)	210 (23.9)	0.152	3.32 [2.51;4.43]	<0.001
Arrhythmia	112 (12.9)	116 (13.2)	0.001	1.02 [0.77;1.34]	0.961
Syncope	221 (25.2)	258 (29.4)	0.039	1.22 [0.99;1.50]	0.074
Hypertensive emergency	33 (3.8)	29 (3.3)	-0.005	0.86 [0.52;1.44]	0.664
**Hospital outcome**					
Inpatient	424 (48.9)	487 (55.5)	0.066	1.30 [1.08;1.58]	0.006
Hospitalization time, days	1 (0-6)	2 (0-7)	0.024	1.00 [0.99;1.01]	0.613
Echocardisocopy	335 (38.6)	303 (34.5)	-0.040	0.84 [0.69;1.02]	0.076
Fibrinolysis	26 (3)	10 (1.1)	-0.018	0.38 [0.17;0.77]	0.006
PIVS	249 (28.7)	157 (17.9)	-0.108	0.54 [0.43;0.68]	<0.001
Emergent surgery	21 (2.4)	18 (2.1)	-0.003	0.85 [0.44;1.60]	0.602
NIMV	26 (3)	70 (8)	0.049	2.79 [1.78;4.51]	<0.001
Mechanical ventilation	65 (7.5)	36 (4.1)	-0.033	0.53 [0.34;0.80]	<0.001
Vasoactive agents	63 (7.3)	55 (6.3)	-0.010	0.85 [0.59;1.24]	0.002
ACCU-admission	268 (30.9)	149 (19)	-0.139	0.46 [0.36;0.57]	<0.001

Abbreviations: NA: not applicable; Ref: reference; NIMV: noninvasive mechanical ventilation; PIVS: percutaneous interventional vascular surgery; ACCU: acute cardiac care unit.

[a]Values expressed as total number (percentage) and medians (25th-75th percentile), as appropriate.

[b]The Mann–Whitney U test or chi-squared test was used as appropriate.

cCohen's d-test was used to estimate the effect size.

[d]Fisher's exact probability statistic was used.

For peer review only

Table 3. Hazard ratio derived from Cox regression.

Variable	Hazard ratio	5% Confidence interval	95% Confidence interval	*p* value
Cohort ≥ 75 years	1.98	1.51	2.60	<0.001
Being in nursing home	1.42	1.07	1.89	0.014
Respiratory rate	1.02	1.01	1.04	<0.001
Systolic blood pressure	0.99	0.98	0.99	0.017
Lactate	1.25	1.20	1.30	<0.001
Noninvasive mechanical ventilation	1.83	1.26	2.65	0.001
Mechanical ventilation	2.53	1.30	4.92	0.006
Cardioversion	2.21	1.22	4.03	0.009
Defibrillation	2.39	1.23	4.64	0.010
Acute heart failure	2.32	1.63	3.31	<0.001
Age-Charlson comorbidity index	1.13	1.09	1.18	<0.001

For peer review only

Figure 1. Study population flowchart

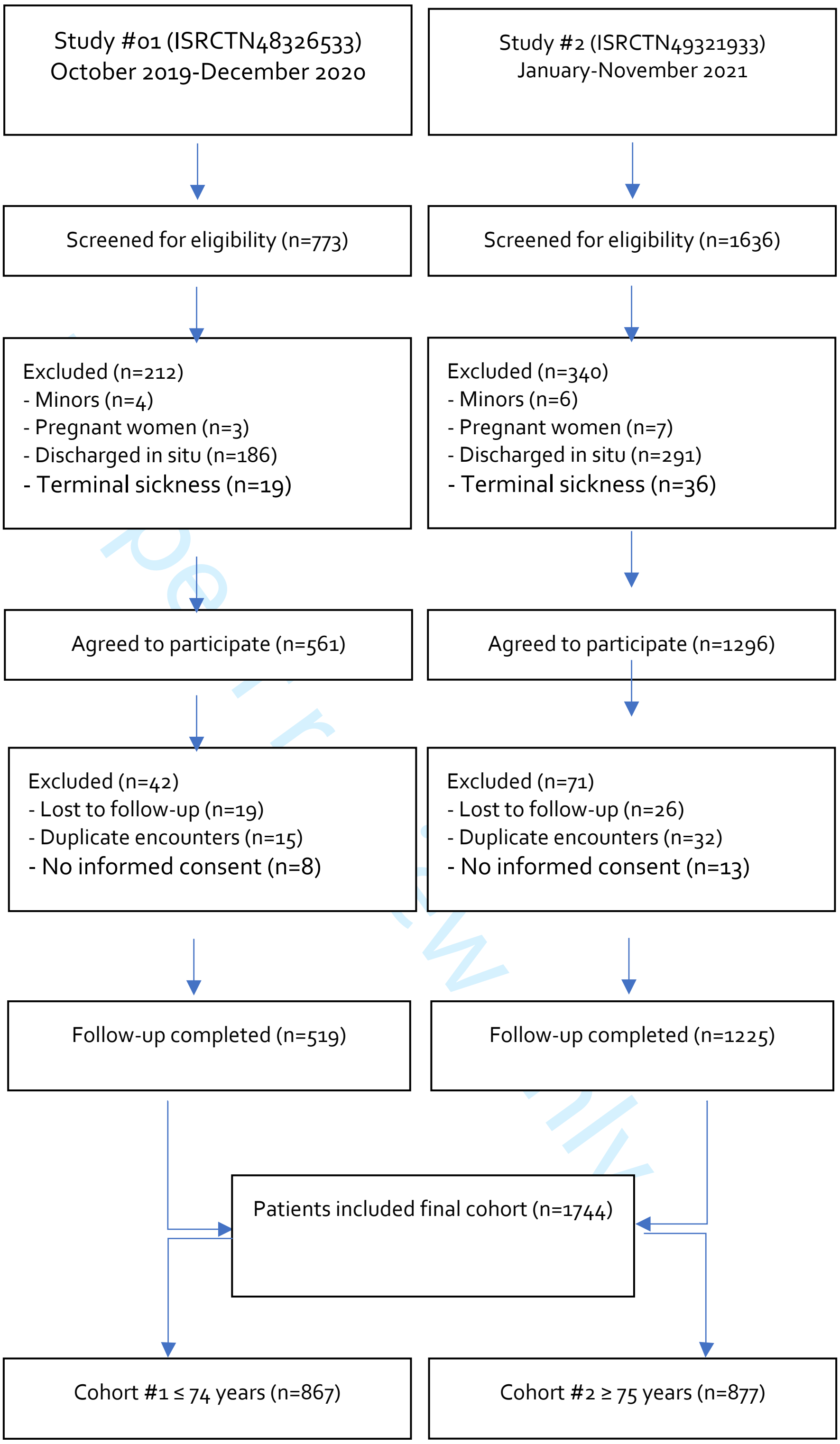

For peer review only - http://bmjopen.bmj.com/site/about/guidelines.xhtml

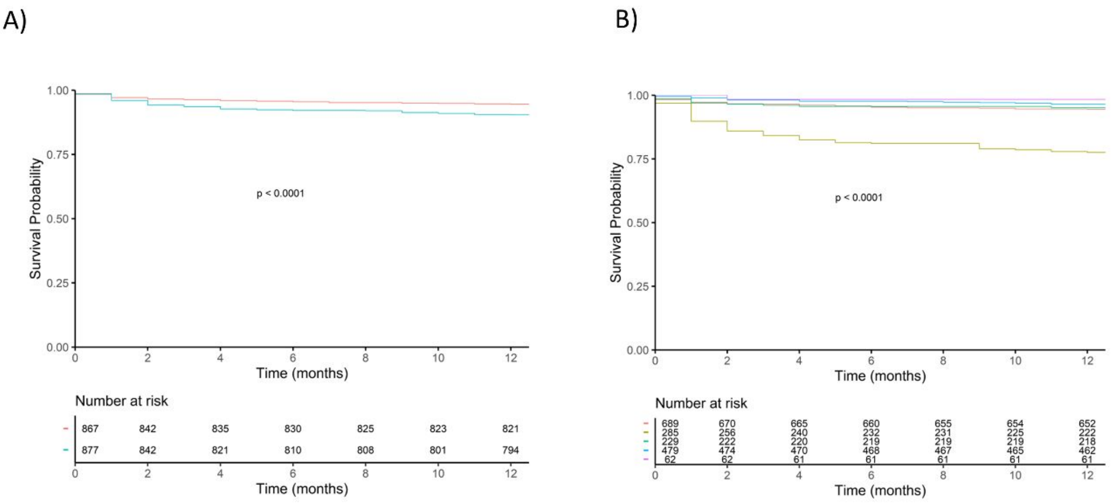

Figure 2. Kaplan–Meier survival curves for age ≤ 74 years vs ≥ 75 years (A) and cardiovascular diagnosis for both groups together (B). Figure 1A, Age ≤ 74 years (red line), and age ≥ 75 years (blue line). Figure 1B, ischemic heart disease (red line), acute heart failure (dark green), arrhythmia (light green), syncope (blue line), hypertensive emergency (purple line).

For peer review only - http://bmjopen.bmj.com/site/about/guidelines.xhtml

Supplementary data

For peer review only

Table of contents

Supplementary methods…..……………………………………………………………..3
Strengthening the Reporting of Observational Studies in Epidemiology (STROBE) Statement…..………..……………………………………………..…3
Supplementary Results.……………………………………………………….……5
- Supplementary Figure 1. Study population flowchart..………………………...5
- Supplementary Table S1. Cumulative mortality by prehospital syndromic suspect………………………………………………………………………………6
- Supplementary Table S2: Summary descriptives table by groups of acute heart failure...…..……………………………………………………………...........7
- Supplementary Table S3: Summary descriptives table by groups of Arrythmmia...……………………………………………………………………8
- Supplementary Table S4: Summary descriptives table by groups of Hypertensive emergency…………………………….................................9
- Supplementary Table S5 Summary descriptives table by groups of ischemic heart disease………..………………………………………………………..10
- Supplementary Table S6: Summary descriptives table by groups of syncope..11
- Supplementary Table S7: Log-regression table by mortality groups…………12
References...14

For peer review only

Supplementary Methods

STROBE[1] Statement—checklist of items that should be included in reports of observational studies

	Item No	Recommendation	Page No
**Title and abstract**	1	(*a*) Indicate the study's design with a commonly used term in the title or the abstract	1
		(*b*) Provide in the abstract an informative and balanced summary of what was done and what was found	3
**Introduction**			
Background/rationale	2	Explain the scientific background and rationale for the investigation being reported	5
Objectives	3	State specific objectives, including any prespecified hypotheses	5
**Methods**			
Study design	4	Present key elements of study design early in the paper	6
Setting	5	Describe the setting, locations, and relevant dates, including periods of recruitment, exposure, follow-up, and data collection	6
Participants	6	(*a*) *Cohort study*—Give the eligibility criteria, and the sources and methods of selection of participants. Describe methods of follow-up<br>*Case-control study*—Give the eligibility criteria, and the sources and methods of case ascertainment and control selection. Give the rationale for the choice of cases and controls<br>*Cross-sectional study*—Give the eligibility criteria, and the sources and methods of selection of participants	6
		(*b*) *Cohort study*—For matched studies, give matching criteria and number of exposed and unexposed<br>*Case-control study*—For matched studies, give matching criteria and the number of controls per case	6
Variables	7	Clearly define all outcomes, exposures, predictors, potential confounders, and effect modifiers. Give diagnostic criteria, if applicable	7
Data sources/ measurement	8*	For each variable of interest, give sources of data and details of methods of assessment (measurement). Describe comparability of assessment methods if there is more than one group	7
Bias	9	Describe any efforts to address potential sources of bias	7
Study size	10	Explain how the study size was arrived at	7
Quantitative variables	11	Explain how quantitative variables were handled in the analyses. If applicable, describe which groupings were chosen and why	7
Statistical methods	12	(*a*) Describe all statistical methods, including those used to control for confounding	7
		(*b*) Describe any methods used to examine subgroups and interactions	7
		(*c*) Explain how missing data were addressed	7
		(*d*) *Cohort study*—If applicable, explain how loss to follow-up was addressed<br>*Case-control study*—If applicable, explain how matching of cases and controls was addressed<br>*Cross-sectional study*—If applicable, describe analytical methods taking account of sampling strategy	7
		(*e*) Describe any sensitivity analyses	7

Continued on next page

For peer review only - http://bmjopen.bmj.com/site/about/guidelines.xhtml

**Results**			
Participants	13*	(a) Report numbers of individuals at each stage of study—eg numbers potentially eligible, examined for eligibility, confirmed eligible, included in the study, completing follow-up, and analysed	8
		(b) Give reasons for non-participation at each stage	8
		(c) Consider use of a flow diagram	8
Descriptive data	14*	(a) Give characteristics of study participants (eg demographic, clinical, social) and information on exposures and potential confounders	8
		(b) Indicate number of participants with missing data for each variable of interest	8
		(c) *Cohort study*—Summarise follow-up time (eg, average and total amount)	8
Outcome data	15*	*Cohort study*—Report numbers of outcome events or summary measures over time	*8*
		Case-control study—Report numbers in each exposure category, or summary measures of exposure	*8*
		Cross-sectional study—Report numbers of outcome events or summary measures	*8*
Main results	16	(*a*) Give unadjusted estimates and, if applicable, confounder-adjusted estimates and their precision (eg, 95% confidence interval). Make clear which confounders were adjusted for and why they were included	9
		(*b*) Report category boundaries when continuous variables were categorized	9
		(*c*) If relevant, consider translating estimates of relative risk into absolute risk for a meaningful time period	9
Other analyses	17	Report other analyses done—eg analyses of subgroups and interactions, and sensitivity analyses	9
**Discussion**			
Key results	18	Summarise key results with reference to study objectives	10-11
Limitations	19	Discuss limitations of the study, taking into account sources of potential bias or imprecision. Discuss both direction and magnitude of any potential bias	11-12
Interpretation	20	Give a cautious overall interpretation of results considering objectives, limitations, multiplicity of analyses, results from similar studies, and other relevant evidence	10,11,12
Generalisability	21	Discuss the generalisability (external validity) of the study results	11,12,13
**Other information**			
Funding	22	Give the source of funding and the role of the funders for the present study and, if applicable, for the original study on which the present article is based	2

*Give information separately for cases and controls in case-control studies and, if applicable, for exposed and unexposed groups in cohort and cross-sectional studies.

Note: An Explanation and Elaboration article discusses each checklist item and gives methodological background and published examples of transparent reporting. The STROBE checklist is best used in conjunction with this article (freely available on the Web sites of PLoS Medicine at http://www.plosmedicine.org/, Annals of Internal Medicine at http://www.annals.org/, and Epidemiology at http://www.epidem.com/). Information on the STROBE Initiative is available at www.strobe-statement.org.

For peer review only

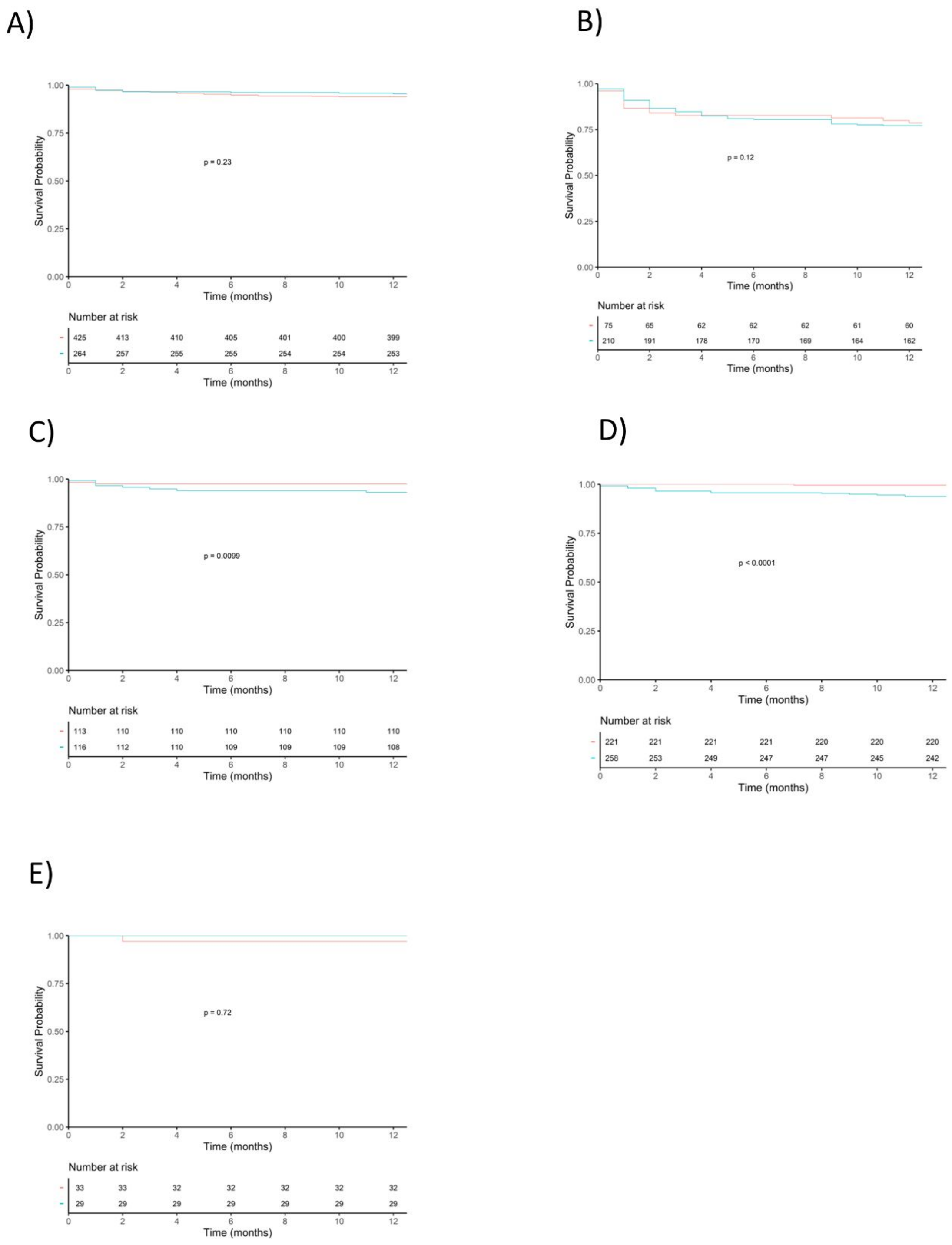

Supplementary Figure S1. Kaplan Meier survival curves for Age <75 years vs ≥ 75 years according to cardiovascular diagnosis. Ischemic heart disease (A), acute heart failure (B), arrhythmia (C), syncope (D), hypertensive emergency (E). Age ≤ 74 years (red line), and age ≥ 75 years (blue line).

Supplementary Table S1. Cumulative mortality by prehospital syndromic suspect

Variable[a]	Ischemic heart disease	Acute heart failure	Arrhythmia	Syncope	Hypertensive emergency	*p* value[b]
**≤ 74 years**	425 (49)	76 (8.8)	112 (12.9)	221 (25.2)	33 (3.8)	
1-day	12 (2.8)	10 (13.2)	3 (2.7)	0	0	0.022
2-day	14 (3.3)	12 (15.8)	3 (2.7)	0	1 (3)	0.033
7-day	24 (5.6)	13 (17.1)	3 (2.7)	1 (0.5)	1 (3)	0.002
30-day	26 (6.1)	21 (27.6)	3 (2.7)	2 (0.9)	1 (3)	0.002
90-day	33 (7.8)	26 (23.2)	7 (6.3)	7 (3.2)	1 (3)	0.012
180-day	38 (8.9)	28 (36.8)	9 (8)	10 (4.5)	3 (9.1)	0.060
365-day	43 (10.1)	33 (43.4)	10 (8.9)	12 (5.4)	3 (9.1)	0.042
**≥ 75 years**	264 (30.1)	210 (23.9)	116 (13.2)	258 (29.4)	29 (3.3)	
1-day	7 (2.7)	19 (9)	4 (3.4)	5 (1.9)	0	0.133
2-day	9 (3.4)	28 (13.3)	5 (4.3)	9 (3.5)	0	0.161
7-day	10 (3.8)	41 (19.5)	7 (6)	11 (4.3)	0	0.100
30-day	17 (6.4)	60 (28.6)	11 (9.5)	18 (7)	0	0.043
90-day	22 (8.3)	86 (41)	16 (13.8)	32 (12.4)	0	0.114
180-day	25 (9.5)	99 (47.1)	20 (17.2)	40 (15.5)	0	0.185
365-day	35 (13.3)	115 (54.8)	26 (22.4)	51 (19.8)	2 (6.9)	0.235

[a]Values expressed as total number (percentage).
[b]Fisher's Exact Probability statistic, was used.

For peer review only

For peer review only - http://bmjopen.bmj.com/site/about/guidelines.xhtml

Supplementary Table S2: Summary descriptives table by groups of acute heart failure

	Not Suffering acute heart failure<br>*N=75*	Suffering acute heart failure<br>*N=210*	OR	p.ratio	p.overall
1-day:					0.406
0	65 (86.7%)	191 (91.0%)	Ref.	Ref.	
1	10 (13.3%)	19 (9.05%)	0.64 [0.29;1.52]	0.304	
2-day:					0.706
0	63 (84.0%)	182 (86.7%)	Ref.	Ref.	
1	12 (16.0%)	28 (13.3%)	0.80 [0.39;1.74]	0.566	
7-day:					0.807
0	62 (82.7%)	169 (80.5%)	Ref.	Ref.	
1	13 (17.3%)	41 (19.5%)	1.15 [0.59;2.37]	0.693	
30-day:					1.000
0	54 (72.0%)	150 (71.4%)	Ref.	Ref.	
1	21 (28.0%)	60 (28.6%)	1.03 [0.57;1.88]	0.934	
90-day:					0.306
0	50 (66.7%)	124 (59.0%)	Ref.	Ref.	
1	25 (33.3%)	86 (41.0%)	1.38 [0.80;2.44]	0.249	
180-day:					0.125
0	48 (64.0%)	111 (52.9%)	Ref.	Ref.	
1	27 (36.0%)	99 (47.1%)	1.58 [0.92;2.75]	0.097	
365-day:					0.096
0	43 (57.3%)	95 (45.2%)	Ref.	Ref.	
1	32 (42.7%)	115 (54.8%)	1.62 [0.95;2.78]	0.074	

0: survival; 1: non-survival

Supplementary Table S3: Summary descriptives table by groups of Arrythmia

	**Not Suffering Arrythmia** *N=113*	**Suffering Arrythmia** *N=116*	**OR**	**p.ratio**	**p.overall**
1-day:					1.000
0	110 (97.3%)	112 (96.6%)	Ref.	Ref.	
1	3 (2.65%)	4 (3.45%)	1.29 [0.26;7.15]	0.748	
2-day:					0.722
0	110 (97.3%)	111 (95.7%)	Ref.	Ref.	
1	3 (2.65%)	5 (4.31%)	1.62 [0.37;8.55]	0.524	
7-day:					0.333
0	110 (97.3%)	109 (94.0%)	Ref.	Ref.	
1	3 (2.65%)	7 (6.03%)	2.28 [0.60;11.4]	0.233	
30-day:					0.060
0	110 (97.3%)	105 (90.5%)	Ref.	Ref.	
1	3 (2.65%)	11 (9.48%)	3.69 [1.10;17.5]	0.034	
90-day:					0.149
0	105 (92.9%)	100 (86.2%)	Ref.	Ref.	
1	8 (7.08%)	16 (13.8%)	2.07 [0.86;5.38]	0.103	
180-day:					0.092
0	103 (91.2%)	96 (82.8%)	Ref.	Ref.	
1	10 (8.85%)	20 (17.2%)	2.12 [0.96;4.99]	0.063	
365-day:					0.015
0	102 (90.3%)	90 (77.6%)	Ref.	Ref.	
1	11 (9.73%)	26 (22.4%)	2.65 [1.26;5.91]	0.010	

0: survival; 1: non-survival

Supplementary Table S4: Summary descriptives table by groups of Hypertensive Emergency

	**Not Suffering Hypertensive Emergency** *N=33*	**Suffering Hypertensive Emergency** *N=29*	**OR**	**p.ratio**	**p.overall**
1-day: 0	33 (100%)	29 (100%)	Ref.	Ref.	.
2-day:					1.000
0	32 (97.0%)	29 (100%)	Ref.	Ref.	
1	1 (3.03%)	0 (0.00%)	. [.;.]	.	
7-day:					1.000
0	32 (97.0%)	29 (100%)	Ref.	Ref.	
1	1 (3.03%)	0 (0.00%)	. [.;.]	.	
30-day:					1.000
0	32 (97.0%)	29 (100%)	Ref.	Ref.	
1	1 (3.03%)	0 (0.00%)	. [.;.]	.	
90-day:					1.000
0	32 (97.0%)	29 (100%)	Ref.	Ref.	
1	1 (3.03%)	0 (0.00%)	. [.;.]	.	
180-day:					0.241
0	30 (90.9%)	29 (100%)	Ref.	Ref.	
1	3 (9.09%)	0 (0.00%)	. [.;.]	.	
365-day:					1.000
0	30 (90.9%)	27 (93.1%)	Ref.	Ref.	
1	3 (9.09%)	2 (6.90%)	0.76 [0.08;5.36]	0.782	

0: survival; 1: non-survival

Supplementary Table S5: Summary descriptives table by groups of ischemic heart disease

	Not suffering ischemic heart disease *N=425*	Suffering ischemic heart disease *N=264*	OR	p.ratio	p.overall
1-day:					1.000
0	413 (97.2%)	257 (97.3%)	Ref.	Ref.	
1	12 (2.82%)	7 (2.65%)	0.95 [0.34;2.41]	0.910	
2-day:					1.000
0	411 (96.7%)	255 (96.6%)	Ref.	Ref.	
1	14 (3.29%)	9 (3.41%)	1.04 [0.42;2.43]	0.925	
7-day:					0.360
0	401 (94.4%)	254 (96.2%)	Ref.	Ref.	
1	24 (5.65%)	10 (3.79%)	0.66 [0.30;1.38]	0.280	
30-day:					0.994
0	399 (93.9%)	247 (93.6%)	Ref.	Ref.	
1	26 (6.12%)	17 (6.44%)	1.06 [0.55;1.98]	0.859	
90-day:					0.902
0	392 (92.2%)	242 (91.7%)	Ref.	Ref.	
1	33 (7.76%)	22 (8.33%)	1.08 [0.61;1.89]	0.785	
180-day:					0.922
0	387 (91.1%)	239 (90.5%)	Ref.	Ref.	
1	38 (8.94%)	25 (9.47%)	1.07 [0.62;1.81]	0.811	
365-day:					0.254
0	382 (89.9%)	229 (86.7%)	Ref.	Ref.	
1	43 (10.1%)	35 (13.3%)	1.36 [0.84;2.18]	0.211	

0: survival; 1: non-survival

Supplementary Table S6: Summary descriptives table by groups of syncope

	Not suffering syncope *N=221*	Suffering syncope *N=258*	OR	p.ratio	p.overall
1-day:					0.065
0	221 (100%)	253 (98.1%)	Ref.	Ref.	
1	0 (0.00%)	5 (1.94%)	. [.;.]	.	
2-day:					0.004
0	221 (100%)	249 (96.5%)	Ref.	Ref.	
1	0 (0.00%)	9 (3.49%)	. [.;.]	.	
7-day:					0.018
0	220 (99.5%)	247 (95.7%)	Ref.	Ref.	
1	1 (0.45%)	11 (4.26%)	8.65 [1.65;214]	0.007	
30-day:					0.002
0	219 (99.1%)	240 (93.0%)	Ref.	Ref.	
1	2 (0.90%)	18 (6.98%)	7.67 [2.16;52.7]	0.001	
90-day:					<0.001
0	214 (96.8%)	226 (87.6%)	Ref.	Ref.	
1	7 (3.17%)	32 (12.4%)	4.24 [1.93;10.8]	<0.001	
180-day:					<0.001
0	211 (95.5%)	218 (84.5%)	Ref.	Ref.	
1	10 (4.52%)	40 (15.5%)	3.82 [1.93;8.31]	<0.001	
365-day:					<0.001
0	209 (94.6%)	207 (80.2%)	Ref.	Ref.	
1	12 (5.43%)	51 (19.8%)	4.24 [2.26;8.58]	<0.001	

0: survival; 1: non-survival

For peer review only - http://bmjopen.bmj.com/site/about/guidelines.xhtml

Supplementary Table S7: Log-regression table by mortality groups

	Survival	Non-Survival	Hazard Ratio [95% Confidence interval]	p.value
	N=1414	***N=330***		
Cohort:				
≤ 74 years	766 (54.2%)	101 (30.6%)	Ref.	Ref.
≥ 75 years	648 (45.8%)	229 (69.4%)	2.41 [1.91;3.04]	<0.001
Sex:				
Male	805 (56.9%)	214 (64.8%)	Ref.	Ref.
Female	609 (43.1%)	116 (35.2%)	0.74 [0.59;0.93]	0.010
Nursing home				
No	1297 (91.7%)	257 (77.9%)	Ref.	Ref.
Yes	117 (8.27%)	73 (22.1%)	2.69 [2.07;3.49]	<0.001
Respiratory Rate	17.9 (6.35)	22.1 (10.5)	1.06 [1.05;1.07]	<0.001
SpO2	95.7 (5.23)	87.2 (13.0)	0.94 [0.93;0.94]	<0.001
SBP	138 (32.7)	123 (40.8)	0.99 [0.98;0.99]	<0.001
DBP	79.0 (19.1)	69.6 (24.5)	0.98 [0.97;0.98]	<0.001
Heart rate	83.5 (31.8)	95.1 (42.9)	1.01 [1.01;1.01]	<0.001
Temperature	36.1 (0.68)	36.1 (0.98)	0.94 [0.81;1.10]	0.424
GCS	14.8 (1.25)	12.8 (4.00)	0.79 [0.77;0.81]	<0.001
Glucose	141 (57.3)	191 (90.8)	1.01 [1.00;1.01]	<0.001
Lactate	1.99 (1.35)	4.63 (3.79)	1.37 [1.33;1.40]	<0.001
NIMV:				
No	1387 (98.1%)	270 (81.8%)	Ref.	Ref.
Yes	27 (1.91%)	60 (18.2%)	6.85 [5.17;9.08]	<0.001
Mechanical ventilation:				
No	1404 (99.3%)	278 (84.2%)	Ref.	Ref.
Yes	10 (0.71%)	52 (15.8%)	14.5 [10.7;19.6]	<0.001
Transcutaneous pacemaker:				

For peer review only - http://bmjopen.bmj.com/site/about/guidelines.xhtml

	Survival	Non-Survival	Hazard Ratio [95% Confidence interval]	p.value
	N=1414	*N=330*		
No	1364 (96.5%)	320 (97.0%)	Ref.	Ref.
Yes	50 (3.54%)	10 (3.03%)	0.91 [0.48;1.70]	0.759
Cardioversion:				
No	1396 (98.7%)	315 (95.5%)	Ref.	Ref.
Yes	18 (1.27%)	15 (4.55%)	3.24 [1.93;5.43]	<0.001
Defibrillation.:				
No	1406 (99.4%)	306 (92.7%)	Ref.	Ref.
Yes	8 (0.57%)	24 (7.27%)	9.04 [5.95;13.7]	<0.001
Vasoactive agents:				
No	1402 (99.2%)	282 (85.5%)	Ref.	Ref.
Yes	12 (0.85%)	48 (14.5%)	13.4 [9.81;18.2]	<0.001
Prehospital syndromic suspect:				
Ischemic heart disease	611 (43.2%)	78 (23.6%)	Ref.	Ref.
Acute heart failure	138 (9.76%)	147 (44.5%)	6.01 [4.57;7.92]	<0.001
Arrhythmia	192 (13.6%)	37 (11.2%)	1.46 [0.98;2.15]	0.060
Syncope	416 (29.4%)	63 (19.1%)	1.16 [0.83;1.61]	0.394
Hypertensive emergency	57 (4.03%)	5 (1.52%)	0.68 [0.28;1.69]	0.412
ACCI	2.31 (2.33)	4.29 (2.96)	1.23 [1.19;1.27]	<0.001

References

1. Vandenbroucke JP, von Elm E, Altman DG, Gøtzsche PC, Mulrow CD, Pocock SJ, Poole C, Schlesselman JJ, Egger M; STROBE Initiative. Strengthening the Reporting of Observational Studies in Epidemiology (STROBE): explanation and elaboration. PLoS Med. 2007 Oct 16;4(10):e297.

For peer review only

STROBE[1] Statement—checklist of items that should be included in reports of observational studies

	Item No	Recommendation	Page No
**Title and abstract**	1	(*a*) Indicate the study's design with a commonly used term in the title or the abstract	1
		(*b*) Provide in the abstract an informative and balanced summary of what was done and what was found	3
**Introduction**			
Background/rationale	2	Explain the scientific background and rationale for the investigation being reported	5
Objectives	3	State specific objectives, including any prespecified hypotheses	5
**Methods**			
Study design	4	Present key elements of study design early in the paper	6
Setting	5	Describe the setting, locations, and relevant dates, including periods of recruitment, exposure, follow-up, and data collection	6
Participants	6	(*a*) *Cohort study*—Give the eligibility criteria, and the sources and methods of selection of participants. Describe methods of follow-up<br>*Case-control study*—Give the eligibility criteria, and the sources and methods of case ascertainment and control selection. Give the rationale for the choice of cases and controls<br>*Cross-sectional study*—Give the eligibility criteria, and the sources and methods of selection of participants	6
		(*b*) *Cohort study*—For matched studies, give matching criteria and number of exposed and unexposed<br>*Case-control study*—For matched studies, give matching criteria and the number of controls per case	6
Variables	7	Clearly define all outcomes, exposures, predictors, potential confounders, and effect modifiers. Give diagnostic criteria, if applicable	7
Data sources/ measurement	8*	For each variable of interest, give sources of data and details of methods of assessment (measurement). Describe comparability of assessment methods if there is more than one group	7
Bias	9	Describe any efforts to address potential sources of bias	7
Study size	10	Explain how the study size was arrived at	7
Quantitative variables	11	Explain how quantitative variables were handled in the analyses. If applicable, describe which groupings were chosen and why	7
Statistical methods	12	(*a*) Describe all statistical methods, including those used to control for confounding	7
		(*b*) Describe any methods used to examine subgroups and interactions	7
		(*c*) Explain how missing data were addressed	7
		(*d*) *Cohort study*—If applicable, explain how loss to follow-up was addressed<br>*Case-control study*—If applicable, explain how matching of cases and controls was addressed<br>*Cross-sectional study*—If applicable, describe analytical methods taking account of sampling strategy	7
		(*e*) Describe any sensitivity analyses	7

Continued on next page

For peer review only

**Results**			
Participants	13*	(a) Report numbers of individuals at each stage of study—eg numbers potentially eligible, examined for eligibility, confirmed eligible, included in the study, completing follow-up, and analysed	8
		(b) Give reasons for non-participation at each stage	8
		(c) Consider use of a flow diagram	8
Descriptive data	14*	(a) Give characteristics of study participants (eg demographic, clinical, social) and information on exposures and potential confounders	8
		(b) Indicate number of participants with missing data for each variable of interest	8
		(c) *Cohort study*—Summarise follow-up time (eg, average and total amount)	8
Outcome data	15*	*Cohort study*—Report numbers of outcome events or summary measures over time	*8*
		Case-control study—Report numbers in each exposure category, or summary measures of exposure	*8*
		Cross-sectional study—Report numbers of outcome events or summary measures	*8*
Main results	16	(*a*) Give unadjusted estimates and, if applicable, confounder-adjusted estimates and their precision (eg, 95% confidence interval). Make clear which confounders were adjusted for and why they were included	9
		(*b*) Report category boundaries when continuous variables were categorized	9
		(*c*) If relevant, consider translating estimates of relative risk into absolute risk for a meaningful time period	9
Other analyses	17	Report other analyses done—eg analyses of subgroups and interactions, and sensitivity analyses	9
**Discussion**			
Key results	18	Summarise key results with reference to study objectives	10-11
Limitations	19	Discuss limitations of the study, taking into account sources of potential bias or imprecision. Discuss both direction and magnitude of any potential bias	11-12
Interpretation	20	Give a cautious overall interpretation of results considering objectives, limitations, multiplicity of analyses, results from similar studies, and other relevant evidence	10,11,12
Generalisability	21	Discuss the generalisability (external validity) of the study results	11,12,13
**Other information**			
Funding	22	Give the source of funding and the role of the funders for the present study and, if applicable, for the original study on which the present article is based	2

*Give information separately for cases and controls in case-control studies and, if applicable, for exposed and unexposed groups in cohort and cross-sectional studies.

Note: An Explanation and Elaboration article discusses each checklist item and gives methodological background and published examples of transparent reporting. The STROBE checklist is best used in conjunction with this article (freely available on the Web sites of PLoS Medicine at http://www.plosmedicine.org/, Annals of Internal Medicine at http://www.annals.org/, and Epidemiology at http://www.epidem.com/). Information on the STROBE Initiative is available at www.strobe-statement.org.

For peer review only

BMJ Open

Prehospital acute life-threatening cardiovascular disease in elderly: An observational, prospective, multicenter, ambulance-based cohort study.

Journal:	*BMJ Open*
Manuscript ID	bmjopen-2023-078815.R1
Article Type:	Original research
Date Submitted by the Author:	13-Sep-2023
Complete List of Authors:	del Pozo Vegas, Carlos; Hospital Clinico Universitario de Valladolid, Emergency Department; Universidad de Valladolid<br>Zalama-Sánchez, Daniel; Hospital Clinico Universitario de Valladolid, Emergency Department<br>Sanz-Garcia, Ancor; University of Castilla-La Mancha - Center for University Studies Talavera de la Reina<br>López-Izquierdo, Raúl; Hosp Univ Rio Hortega<br>Sáez-Belloso, Silvia; Universidad de Valladolid<br>Oleaga, Cristina Mazas Perez; Universidad Europea del Atlántico; Universidad Internacional Iberoamericana<br>Azpíroz, Irma Domínguez; Universidad Europea del Atlántico; Universidad Internacional Iberoamericana<br>Pascual, Iñaki Elío; Universidad Europea del Atlántico; Universidade Internacional do Cuanza Cuito<br>Martín-Rodríguez, Francisco; Universidad de Valladolid
**Primary Subject Heading**:	Emergency medicine
Secondary Subject Heading:	Cardiovascular medicine
Keywords:	ACCIDENT & EMERGENCY MEDICINE, Aged, 80 and over, CARDIOLOGY

SCHOLARONE™
Manuscripts

I, the Submitting Author has the right to grant and does grant on behalf of all authors of the Work (as defined in the below author licence), an exclusive licence and/or a non-exclusive licence for contributions from authors who are: i) UK Crown employees; ii) where BMJ has agreed a CC-BY licence shall apply, and/or iii) in accordance with the terms applicable for US Federal Government officers or employees acting as part of their official duties; on a worldwide, perpetual, irrevocable, royalty-free basis to BMJ Publishing Group Ltd ("BMJ") its licensees and where the relevant Journal is co-owned by BMJ to the co-owners of the Journal, to publish the Work in this journal and any other BMJ products and to exploit all rights, as set out in our licence.

The Submitting Author accepts and understands that any supply made under these terms is made by BMJ to the Submitting Author unless you are acting as an employee on behalf of your employer or a postgraduate student of an affiliated institution which is paying any applicable article publishing charge ("APC") for Open Access articles. Where the Submitting Author wishes to make the Work available on an Open Access basis (and intends to pay the relevant APC), the terms of reuse of such Open Access shall be governed by a Creative Commons licence – details of these licences and which Creative Commons licence will apply to this Work are set out in our licence referred to above.

Other than as permitted in any relevant BMJ Author's Self Archiving Policies, I confirm this Work has not been accepted for publication elsewhere, is not being considered for publication elsewhere and does not duplicate material already published. I confirm all authors consent to publication of this Work and authorise the granting of this licence.

For peer review only

Prehospital acute life-threatening cardiovascular disease in elderly: An observational, prospective, multicenter, ambulance-based cohort study.

Carlos del Pozo Vegas[1,2], Daniel Zalama-Sánchez[1], Ancor Sanz-García[3,*], Raúl López-Izquierdo[2,4,5], Silvia Sáez-Belloso [6,7], Cristina Mazas Pérez-Oleaga[8,9,10]; Irma Domínguez Azpíroz[8,11,12]; Iñaki Elío Pascual[8,12,13]; Francisco Martín-Rodríguez[2,6]

[1] Emergency Department. Hospital Clínico Universitario. Valladolid, Spain.

[2] Faculty of Medicine. Universidad de Valladolid, Valladolid, Spain.

[3] Faculty of Health Sciences, Universidad de Castilla la Mancha, Talavera de la Reina, Spain.

[4] Emergency Department. Hospital Universitario Rio Hortega. Valladolid, Spain

[5] CIBER of Respiratory Diseases, Instituto de Salud Carlos III, C/Sinesio Delgado, 4 28029, Madrid, Spain.

[6] Advanced Life Support, Emergency Medical Services (SACYL), Valladolid, Spain.

[7] Faculty of Nursing. Universidad de Valladolid, Valladolid, Spain.

[8] Universidad Europea del Atlántico. Isabel Torres 21 39011 Santander, Spain.

[9] Universidad Internacional Iberoamericana. Arecibo, Puerto Rico 00613, USA.

[10] Universidad de La Romana. La Romana, República Dominicana

[11] Universidad Internacional Iberoamericana. Campeche 24560, México.

[12] Universidade Internacional do Cuanza. Cuito, Bié, Angola

[13] Fundación Universitaria Internacional de Colombia. Bogotá, Colombia

[*] *Corresponding author*

Corresponding author:

Dr. Ancor Sanz-García
Faculty of Health Sciences, Universidad de Castilla la Mancha. Avda. Real Fábrica de Seda, s/n 45600, Talavera de la Reina, Toledo
e-mail: ancor.sanz@gmail.com
Phone: 925 721 010

Running head

Prehospital cardiovascular disease in elderly

For peer review only - http://bmjopen.bmj.com/site/about/guidelines.xhtml

Abstract

Objective

The aim was to explore the association of demographic and prehospital parameters with short- and long-term mortality in acute life-threatening cardiovascular disease by using a hazard model, focusing on elderly individuals, by comparing patients under 75 years versus patients over 75 years of age.

Design

Prospective, multicenter, observational, study.

Setting

Emergency medical services (EMS) delivery study gathering data from two back-to-back studies between October 1, 2019, and November 30, 2021. Six advanced life support (ALS), forty-three basic life support (BLS), and five hospitals in Spain were considered.

Participants

Adult patients suffering from acute life-threatening cardiovascular disease attended by the EMS.

Primary and secondary outcome measures

The primary outcome was in-hospital mortality from any cause within the first to the 365 days following EMS attendance. The main measures included prehospital demographics, biochemical variables, prehospital advanced life support techniques used, and syndromic suspected conditions.

Results

A total of 1744 patients fulfilled the inclusion criteria. The 365-day cumulative mortality in the elderly amounted to 26.1% (229 cases) vs 11.6% (11.6%) in patients under 75 years old. Elderly patients (≥ 75 years) presented a 2-fold risk of mortality compared to patients ≤ 74 years. Life-threatening interventions (mechanical ventilation, cardioversion and defibrillation) were also related to a 2-fold increased risk of mortality. Importantly, patients suffering from acute heart failure presented a more than 2-fold increased risk of mortality.

Conclusions

This study revealed the prehospital variables associated with the long-term mortality of patients suffering from acute cardiovascular disease. Our results provide important insights for the development of specific codes or scores for cardiovascular diseases to facilitate the risk of mortality characterization.

For peer review only - http://bmjopen.bmj.com/site/about/guidelines.xhtml

Keywords Prehospital; Emergency Medical Services; elderly; cardiovascular disease; mortality.

Strengths and limitations of this study

-How the elderly affects cardiovascular patients treated by emergency medicine services is unknown

-This study revealed the prehospital variables associated with the long-term mortality of patients suffering from acute cardiovascular disease.

-The strengths of this study are sample size, the study design with a reduced loss-to-follow-up rate, and it was conducted in both rural and urban areas

-The parameters associated with mortality can be easily accessed by EMS staff, allowing generalization to other health systems.

-This generalizability could lead to scores or specific detection codes.

For peer review only

Introduction

Cardiovascular diseases represent the leading cause of prehospital care, involving a surprising number of unplanned hospitalizations and sudden unexplained mortality (1). Emergency medical services (EMS) must handle this overwhelming patient workload quickly and efficiently, following clinical guidelines and under recognized training, e.g., basic and advanced cardiac life support (BCLS and ACLS) (2) (3).

The setup and implementation of specific detection codes for life-threatening conditions, e.g., cardiorespiratory arrest, ST-elevation coronary syndrome, and stroke, is a well-established procedure in health systems, and EMS plays an active role in detection, emergency critical care, and assisted transfer to a suitable hospital (4). Predefined standard protocols should be applied in diseases with clear symptomatology or well-defined guiding symptoms, and in general, no major operational problems are encountered. The handicap for EMS providers consists of the identification on-scene or *en* route of patients with acute life-threatening cardiovascular disease that is apparently masked. Early warning scores and point-of-care testing can provide an effective support tool to help at critical junctures in the complicated decision-making process (5) (6). However, the identification of high-risk subjects is challenging, particularly when distracting factors such as comorbidities or age come into play (7).

EMS has increasingly switched types of patients, with older adults becoming a major focus area of care (8). Defining the concept of older adults is complex, as there are different categories and timelines, and there is no standard criterion to say that an older person is considered elderly (9). However, there is a general acceptance that persons over 65 years of age are considered elderly, and persons over 75 years of age are classified as late elderly. On the other hand, life expectancy and ageing control have improved considerably, with age-related comorbidities usually appearing later, which is the main reason why the 75-year age limit has been selected to differentiate the cohorts of the present work (10). Among other reasons, falls, drug-taking mistakes, and exacerbations of chronic pathologies are more frequent in elderly individuals, i.e., EMS providers can attend to cases of older adults with

For peer review only - http://bmjopen.bmj.com/site/about/guidelines.xhtml

comorbidities and multimedication. In particular, cardiovascular diseases constitute the first cause of emergency appointments and inpatient hospitalization in older adults, affecting both males and females (11). Life expectancy has increased significantly, meaning that older adults with atrial fibrillation, acute coronary syndrome, congestive heart failure, valvular heart disease, and other cardiovascular processes are becoming increasingly prevalent (12). Complications related to aging can hamper anamnesis and clinical examination and sometimes disrupt responsiveness mechanisms, e.g., 20% of older adults exhibit atypical symptoms of acute coronary syndrome (13). Additionally, coexisting comorbidities may trigger interactions in the cardiovascular system, such as anemia, chronic kidney disease, or diabetes (14). Comorbidities naturally rise sharply with age and necessitate an appropriate analysis. Nonetheless, at 75 years old, cardiovascular pathology in particular showed considerable progression with significant increases in comorbidities, the emergence of additional diseases and the exacerbation of already prevalent pathologies. The group of elderly individuals over 75 years old constitutes a cluster of special follow-ups that could be especially worthwhile for an in-depth characterization (10) (15).

The purpose of the study was to explore the association of demographic and prehospital parameters with short- and long-term mortality in acute life-threatening cardiovascular disease by using a hazard model. In particular, we focused on the elderly by comparing two age cohorts: under 75 years versus over 75 years.

For peer review only - http://bmjopen.bmj.com/site/about/guidelines.xhtml

Methods

Study design

This prospective, multicenter, observational, EMS delivery analysis gathers inputs obtained from two back-to-back studies, "Prehospital identification of prognostic biomarkers in time-dependent diseases" (HITScore) and "Identification of biomarkers of clinical-risk deterioration in prehospital care" (preBIOs).

Ethical approval was granted by the Rio Hortega University Hospital Ethics Committee, references PI041-19 and PI217-20 (principal investigator's reference center. This authorization applies to the other locations covered by the study). This study was conducted according to STrengthening the Reporting of OBservational studies in Epidemiology (Supplementary data p3) (16) and the Declaration of Helsinki.

Study setting

The study was conducted in four Spanish provinces (Burgos, Salamanca, Segovia, and Valladolid), enrolling uninterrupted adults (>18 years) with a syndromic cardiovascular suspect who were transferred by ambulance to the Emergency Department (ED) between October 1, 2019, and November 30, 2021. Global community medical care was provided by the Public Health System (SACYL) and included the Emergency Coordination Center (1-1-2 phone backup), six advanced life support (ALS), forty-three basic life support (BLS), and five hospitals (one minor general district hospital and four university tertiary hospitals). Normally, BLS is staffed by two emergency medical technicians (EMTs), performing on-scene or *en route* BCLS work-up protocols, and ALS is made up of two EMTs, an emergency registered nurse (ERN) and a physician, conducting ACLS operations.

No exception, all cases were examined by an ALS, and following the assessment and diagnostic tests, the physician determined, in line with current guidelines and according to the individual clinical situation, the need for transfer to the ED, as well as the type of ambulance: BLS or ALS. All hospitals presented the Acute Cardiac Care Unit (ACCU), and three hospitals presented the cardiac intervention room 24/7 and emergency cardiac surgery unit. Patients who required emergency hemodynamic studies or advanced cardiologic care and for whom these facilities were not available at the reference hospital were evacuated as top priority (daytime by Helicopter Emergency Medical Service and nighttime by ALS, mandatory with turnaround times under 1 hour) to other hospitals included in the study.

Population

For peer review only - http://bmjopen.bmj.com/site/about/guidelines.xhtml

Recruitment was consecutive. Participants enrolled in the study were defined as adults (>18 years) with acute cardiovascular disease (prehospital syndromic suspected condition) managed by EMS and transferred to the ED. Noncardiovascular disorders, minors, pregnant women (known or apparent), terminally ill patients (condition confirmed by a medical report), or on-site discharge were excluded.

An informed consent form, managed by the ERN and applicable for the entire follow-up study, was reviewed and countersigned by all participants. In the absence of appropriate understanding by the patient, a research associate in the ED tried to collect the consent form signed by the patient or by a family member or legal guardian. If, despite all efforts, consent was not obtained, the patient was removed and excluded from the study.

Data collection

Mandatory on-site hands-on training was conducted for all staff before the study started and included the standardized procedure for taking vital signs, handling, calibration, and cleaning of the point-of-care testing device, as well as data input to a database specially created for this purpose. A specific database was designed, with access by individual password and double authentication. In this database, we entered both the data collected from the EMS medical record and later the data from hospital care and subsequent follow-up by reviewing the electronic medical record (in two steps, at 30 and 365 days). Once the data had been linked, the data manager anonymized the patient identifiers.

Age, sex, nursing home location, and on-scene vital signs (respiratory rate, oxygen saturation, systolic and diastolic blood pressure, heart rate, temperature, Glasgow Coma Scale, glucose, lactate, and electrocardiogram) were collected and recorded by the ERN. A LifePAK® 15 monitor-defibrillator (Physio-Control, Inc., Redmond, USA) was applied to obtain oxygen saturation, blood pressure, heart rate and electrocardiogram. A ThermoScan® PRO 6000 thermometer (Welch Allyn, Inc., Skaneateles Falls, USA) was used to collect temperature, and finally, the analyser epoc® (Siemens Healthcare GmbH, Erlangen Germany) was employed to perform prehospital analysis.

The physician subsequently checked the electrocardiogram and recorded the baseline heart rhythm, as well as the 17 comorbidities categories required to calculate the Age-Charlson comorbidity index (ACCI), composed of myocardial infarction, congestive heart failure, peripheral vascular disease, stroke or transient ischemic attack, dementia, chronic obstructive pulmonary disease, connective tissue disease peptic ulcer disease, mild liver disease,

For peer review only - http://bmjopen.bmj.com/site/about/guidelines.xhtml

uncomplicated diabetes mellitus, hemiplegia, moderate to severe chronic kidney disease, diabetes mellitus with end-organ damage, localized solid tumor, leukemia, lymphoma, moderate to severe liver disease, metastatic solid tumor, and acquired immunodeficiency syndrome.

Outcomes

The primary outcome was cumulative mortality, from prehospital care to one year of follow-up, segregating time periods as follows: 1, 2, 7, 30, 90, 180, and 365 days. In addition, a comparison was performed matching two age-typed cohorts, a group of ≤ 74 years vs a group of ≥ 75 years, with age discrimination in line with similar reports (17) (18).

The secondary prehospital outcomes included advanced airway management (noninvasive or invasive mechanical ventilation), electrical therapy (transcutaneous pacemaker, cardioversion, or defibrillation) and/or vasoactive agents. Finally, the ALS physician appointed the prehospital syndromic suspected condition, involving ischemic heart disease, acute heart failure, arrhythmia, syncope, or hypertensive emergency.

The secondary hospital outcomes were collected from the electronic medical records obtained at the one-year follow-up and comprised cumulative mortality (all-cause), admission rate, echocardisocopy, percutaneous interventional vascular surgery, emergent surgery, advanced airway management, vasoactive agents, and ACCU admission.

Data analyses

Descriptive results and the associations between age and cardiovascular diagnosis with the variables were assessed by the Mann–Whitney U test or the chi-squared test, when appropriate, and the effect size in the form of standardized mean difference was provided. Absolute values and percentages were used for categorical variables, and median interquartile ranges (IQR) were used for continuous variables because they did not follow a normal distribution. The procedure to determine those variables associated with mortality was as follows: First, a log-rank univariate analysis was performed. Then, a Cox regression (which included only those variables with $p<0.001$ in the log-rank univariate analysis) was performed to evaluate the association of demographic and prehospital parameters with mortality. The Cox regression results were expressed as the hazard ratio and 95% confidence interval (CI). Furthermore, survival according to age, cardiovascular diagnosis, and the combination of both variables was obtained using the Kaplan–Meier method (KM). The cumulative mortality by prehospital syndromic suspect and the difference between groups were assessed by the chi-

For peer review only - http://bmjopen.bmj.com/site/about/guidelines.xhtml

squared test. Finally, the descriptive statistics and association of mortality at 1, 2, 7, 30, 90, 180 and 365 days for each prehospital syndromic suspect were assessed by univariate comparison and expressed as odds ratios and 95% CIs.

Data for prehospital covariates were prospectively collected and registered in a database generated with IBM SPSS Statistics for Apple version 20.0 software. (IBM Corp, Armonk USA). The caseload entry system was tested to delete unclear or ambiguous items and to verify the adequacy of the data-gathering system. The data present missing values completely at random; therefore, the strategy used (listwise deletion) does not imply biased means, variances or regression weights. The statistical power (from 1 to 100) of the present study is 89.4 based on the following considerations: (i) the sample used is n = 517, (ii) significant level of p = 0.05, (iii) expected odds ratios (OR) of 0.405, and (iv) 14% of events.

Patient and Public Involvement

No patient involved

For peer review only

Results

Among 2409 cases, 1744 patients with a syndromic cardiovascular suspect managed by EMS and transferred to the ED were finally included in the final analysis. There were 867 patients from cohort #1 (≤ 74 years) and 877 from cohort #2 (≥ 75 years), 49.7% vs 50.3% (figure 1). Cohort #1 was characterized by a median age of 62 years (IQR: 52-69), and 33.1% (287 cases) were females. Half of the cases (56.2%) showed sinus rhythm, and the comorbidity burden resulted in an ACCI of 1 point (IQR: 0-3), especially myocardial infarction (27.3%), chronic pulmonary disease (21.8%) and nonmetastatic solid tumors (13.7%). In contrast, cohort #2 was described by a median age of 84 years (IQR: 79-88), 49.9% (438 cases) were female, and one-fifth lived in nursing homes. Despite sinus rhythm being the most common baseline cardiac rhythm (38.5%), the ratio of atrial fibrillation (33.4%) is noteworthy. The ACCI score was significantly higher, with a median of 3 points (IQR: 2-5) and a very pronounced prevalence of myocardial infarction (36.6%), congestive heart failure (31.8%), and nonmetastatic solid tumors (23.8%) (table 1).

The 365-day cumulative mortality in the elderly amounted to 26.1% (229 cases) vs 11.6% (11.6%) in patients under 75 years old. Cohort #1 presented a significantly increased percentage of mechanical ventilation, defibrillation, cardioversion, percutaneous interventional vascular surgery, and ACCU admission vs cohort #2, with a relatively increased incidence of noninvasive mechanical ventilation and transcutaneous pacemakers. Concerning prehospital syndromic suspected conditions, half of the patients under 75 years old presented with ischemic heart disease (49%), followed by syncope (25.2%). The elderly also showed ischemic heart disease (30.1%) and syncope (29.4%), stressing in particular the elevated incidence of acute heart failure (23.9% vs. 8.8%, respectively, intercohort) (table 2). Cumulative mortality by prehospital syndromic suspected condition is reported in Supplementary Table S1, as only those patients under 75 years presented statistically significant differences between the prehospital syndromic suspected conditions. The OR of each prehospital syndromic suspected condition for each mortality interval can be found in Supplementary Tables S2 to S6.

The Cox regression (Table 3) that included all those variables with p<0.001 in the long-rank analysis (supplementary table S7) showed that being elderly, being in a nursing home, high respiratory rate, low systolic blood pressure, high levels of lactate, use of noninvasive mechanical ventilation, mechanical ventilation, cardioversion defibrillation, suffering from

For peer review only - http://bmjopen.bmj.com/site/about/guidelines.xhtml

acute heart failure and an elevated Age-Charlson comorbidity index were variables statistically significantly associated with mortality. The aforementioned results for elderly individuals and those with acute heart failure are illustrated in Figure 2. Figure 2A shows the survival curve of patients ≤ 74 years vs ≥ 75 years, with statistically significant differences ($p<0.001$) that appear at the beginning of the follow-up and remain stable throughout time. Figure 2B shows the mortality curves for each prehospital syndromic suspected condition, with acute heart failure presenting the highest mortality that started at the beginning of the follow-up and remained stable over time. When analysing the results according to age, those patients suffering from arrhythmia and syncope presented statistically significant differences in the mortality curves (Supplementary Figure S1) but not the other conditions. This was also the case when considering the association of each particular condition with mortality at 1, 2, 7, 30, 90, 180 and 365 days (Supplementary Tables S2 to S6); only arrhythmia and syncope presented statistically significant time points, arrhythmia only at 365-day mortality, and syncope at all time points except for 1-day mortality.

For peer review only

For peer review only - http://bmjopen.bmj.com/site/about/guidelines.xhtml

Discussion

This prospective, multicenter, observational, EMS delivery study assessed the association of prehospital variables, particularly elderly variables, with short- and long-term mortality in patients with acute life-threatening cardiovascular diseases. Elderly patients (≥ 75 years) presented a 2-fold risk of mortality compared to patients ≤ 74 years. Life-threatening interventions (mechanical ventilation, cardioversion, and defibrillation) were also related to a 2-fold increased risk of mortality. Importantly, patients suffering from acute heart failure presented a more than 2-fold increased risk of mortality.

Cardiovascular diseases are the leading cause of EMS attendance (1) and are associated with high morbidity and mortality (19). Therefore, the accurate and quick characterization of patients at the first contact could provide critical information for the development of specialized risk scores (20) or specific detection codes (4). In this sense, the present study aims to describe the association of prehospital variables with mortality, particularly in elderly individuals, which is one of the main risk factors for cardiovascular diseases (21). Our results align with this evidence, since the categorization of patients ≤ 74 years and ≥ 75 years revealed that older patients presented a higher rate of mortality. Moreover, other factors related to elderly individuals, such as being in nursing homes or the number of comorbidities, measured by the ACCI, were risk factors for mortality. As expected, the higher the number of comorbidities, the higher the risk of mortality. This is not surprising since several comorbidities worsen cardiovascular conditions (14). In fact, comorbidities make patient examination difficult, which is particularly true in the prehospital setting (22). Strikingly, the leap to worse long-term mortality outcomes occurs above 75 years old, whereas below this age, short-term mortality outcomes take prominence.

Despite all this evidence, the effect of age on mortality was not found for short-term mortality, and no statistically significant effect was found for one- or two-day mortality; instead, the statistical significance was relevant at 30 days onwards. This short-term mortality result is somewhat surprising, since it has been previously described at the prehospital level that older ages are associated with higher short-term mortality (23). Perhaps this difference could be explained by the fact that those patients were characterized as suffering mainly from respiratory diseases (23). The long-term mortality relationship with age has already been described in the prehospital setting (24).

For peer review only - http://bmjopen.bmj.com/site/about/guidelines.xhtml

Lactate was statistically related to long-term mortality. Lactate value as a predictor of mortality is well documented in prehospital critical care and represents a very powerful indicator of mitochondrial hypoperfusion, directly affecting the production of bioavailable energy for all physiological processes, including the cardiovascular system (25). This has also been reported, as for respiratory rate and age, for long-term mortality when dealing with all the patients and not with disease-specific analysis (24). Enriquez de Salamanca Gambara et al. (24) also found that GCS and SpO2 were associated with mortality. Perhaps GCS was more related to neurological diseases than cardiovascular diseases, which is our case. A similar argument could be made for SpO2, which could be related to respiratory diseases.

Life-threatening interventions are closely related to patients suffering from cardiovascular disease (26), which was also our case, since noninvasive mechanical ventilation, mechanical ventilation, cardioversion, and defibrillation were procedures present in the nonsurvivor group. Patients who must undergo advanced life support interventions on scene or en route, even overcoming the situation that originated the life-threatening intervention, are also negatively impacted in the long term. In other words, in patients required to receive more aggressive manoeuvres, special caution should be applied regarding evolution, and even more so in elderly and frail patients.

Particularly relevant is the fact that the mortality of each prehospital syndromic suspected condition was different when considering elderly individuals. As expected, acute heart failure was the main condition related to death; however, when analysing mortality according to age groups, only arrhythmia and syncope presented statistically significant differences. This should be interpreted as the higher the age, the higher the probability of death, but only for those two conditions. The other conditions should be treated independently of age. The results for syncope and arrhythmia could be explained by the increased incidence with age, particularly at 70 years for syncope (27) and arrythmias, especially for atrial fibrillation (28).

Our study has some strengths, including the sample size, novelty, and study design with a reduced loss-to-follow-up rate. It was conducted in both rural and urban areas, and our results could be generalizable to other health systems. This last point is based on the fact that all parameters associated with mortality can be easily accessed by the EMS staff. Only lactate determination will require a point-of-care device, which is now a reality in several EMSs (29). This generalizability could lead to scores or specific detection codes. However, some limitations must be considered. First, this is an observational study, so we cannot rule out the

For peer review only - http://bmjopen.bmj.com/site/about/guidelines.xhtml

possibility of selection bias, although participating centers had previous experience and enrolled consecutive patients with prehospital acute cardiovascular disease. To obtain a representative sampling, cases were collected 24/7/365 nonstop in urban, semiurban and rural areas and in different ambulance stations. Second, possible bias may exist in relation to case inclusion in the study. All prehospital acute cardiovascular conditions were included; however, certain disorders may raise uncertainty, i.e., stomachache could be labelled as a digestive disease at prehospital care, although following complementary in-hospital tests (imaging studies, laboratory tests, etc.), the disease ends up being categorized as ischemic heart disease. Nevertheless, the limitation was dampened for two reasons. On-scene, ALS physicians could issue up to a maximum of three diagnostic suspicions, and therefore, any case with a diagnosis of acute cardiovascular disease was eventually included in the analysis. In addition, the diagnostic coincidence between prehospital syndromic cardiovascular suspect (ischemic heart disease, acute heart failure, arrhythmia, syncope, and hypertensive emergency) and the final in-hospital diagnosis was very consistently strong. Third, data extractors were not blinded. To avoid cross-contamination, EMS providers lacked access to hospital follow-up data, and hospital investigators remained blinded to prehospital care data. To accurately link the data between prehospital care and hospital follow-up, at least 5 of the following extractors had to be an exact match: health system ID card, incident reference (EMS-register), first and last name, age, sex at birth, date, and/or time of arrival at the ED. Total access to the master database was given exclusively by the principal investigator and data manager. Fourth, modifiable lifestyles (smoking, alcohol consumption, weight, diet quality and physical activity) or medication history are indeed associated with short- and long-term mortality in life-threatening acute cardiovascular disease. Nevertheless, these data could not be collected in prehospital care or during the in-hospital follow-up phase. For subsequent investigations, a postindex event interview procedure will be implemented to collect these data and thus be able to analyse the influence of these covariates on the final outcome. Finally, an adequate sample size was used in the present preliminary study, but multicenter studies in different health systems are needed to confirm the generalizability of the results.

In summary, this study revealed the prehospital variables associated with the long-term mortality of patients suffering from acute cardiovascular disease. Elderly, life-threatening interventions, acute heart failure, comorbidities, and lactate should be considered when EMS

For peer review only - http://bmjopen.bmj.com/site/about/guidelines.xhtml

faces patients with acute life-threatening cardiovascular diseases. In particular, the effect of elderly patients presenting arrhythmia or syncope should be considered. Finally, our results could pave the way for the development of specific codes or scores for cardiovascular diseases to facilitate the risk of mortality characterization.

For peer review only

For peer review only - http://bmjopen.bmj.com/site/about/guidelines.xhtml

Authors' Contributions

CRediT authorship contribution statement:

Francisco Martín-Rodríguez conceptualized the project, managed and coordinated the project, assisted with the design of methodology, analysed data, and prepared the initial and final drafts of the manuscript. Ancor Sanz-García took responsibility for the data and their analysis. Carlos del Pozo Vegas, Daniel Zalama-Sánchez, Silvia Sáez-Belloso, and Raúl López Izquierdo assisted with the management and coordination of the project, assisted with the design of the methodology, and helped review the manuscript. All authors performed a critical review and approved the final manuscript for interpretation of the data and important intellectual input.

Summarized conflict of interest statements

All signing authors meet the requirements of authorship and have declared the nonexistence of potential conflicts of interest. Daniel Zalama-Sánchez, Ancor Sanz-García, Carlos del Pozo Vegas, Raúl López-Izquierdo, Silvia Sáez-Belloso, and Francisco Martín-Rodríguez report no conflicts of interest.

The authors have no disclosures to make.

On behalf of the other authors, the corresponding author guarantees the accuracy, transparency, and honesty of the data and information contained in the study, that no relevant information has been omitted and that all discrepancies between authors have been adequately resolved and described.

Funding information

This work was supported by the Gerencia Regional de Salud, Public Health System of Castilla y León (Spain) [grant numbers GRS 1903/A/19 and GRS 2131/A/20].

Sponsor role: none

Ethics approval

Ethical approval was granted by the Rio Hortega University Hospital Ethics Committee, references PI041-19 and PI217-20 (principal investigator's reference center. This authorization applies to the other locations covered by the study).

Notation of prior abstract publication/presentation

For peer review only

This article is an original work, has not been published before and is not being considered for publication elsewhere in its final form, in either printed or electronic media. It is not based on any previous communication to a society or meeting.

Data Availability

The data that support the findings of this study are available on request from the corresponding author ASG. The data are not publicly available due to restrictions, and their information could compromise the privacy of research participants.

For peer review only

For peer review only - http://bmjopen.bmj.com/site/about/guidelines.xhtml

References

1. Townsend N, Kazakiewicz D, Lucy Wright F, Timmis A, Huculeci R, Torbica A, et al. Epidemiology of cardiovascular disease in Europe. Nat Rev Cardiol. 2022; 19(2): p. 133-143.
2. Perkins GD, Graesner JT, Semeraro F, Olasveengen T, Soar J, Lott C, et al. European Resuscitation Council Guidelines 2021: Executive summary. Resuscitation. 2021; 161: p. 1-60.
3. Wyckoff MH, Singletary EM, Soar J, Olasveengen TM, Greif R, Liley HG, et al. 2021 International Consensus on Cardiopulmonary Resuscitation and Emergency Cardiovascular Care Science With Treatment Recommendations: Summary From the Basic Life Support; Advanced Life Support; Neonatal Life Support; Education, Implementation, and Teams. Circulation. 2022; 145(9): p. e645-e721.
4. Andersson HB, Seth M, Gurm HS, Bates ER. Pre-hospital Transport Times and Outcomes After Different Reperfusion Strategies for ST-Elevation Myocardial Infarction. Am J Cardiol. 2019; 123(3): p. 375-381.
5. Pirneskoski J, Lääperi M, Kuisma M, Olkkola KT, Nurmi J. Ability of prehospital NEWS to predict 1-day and 7-day mortality is reduced in older adult patients. Emerg Med J. 2021; 38(12): p. 913-918.
6. Martín-Rodríguez F, López-Izquierdo R, Castro Villamor MA, Del Pozo Vegas C, Delgado Benito MDP, Martínez Caballero CM, et al. The Prognostic Value of Prehospital Blood Lactate Levels to Predict Early Mortality in Acute Cardiovascular Disease. Shock. 2020; 53(2): p. 164-170.
7. Forman DE, Maurer MS, Boyd C, Brindis R, Salive ME, Horne FM, et al. Multimorbidity in Older Adults With Cardiovascular Disease. J Am Coll Cardiol. 2018; 71(19): p. 2149-2161.
8. Probst MA, Gibson T, Weiss RE, Yagapen AN, Malveau SE, Adler DH, et al. Risk Stratification of Older Adults Who Present to the Emergency Department With Syncope: The FAINT Score. Ann Emerg Med. 2020; 75(2): p. 147-158.
9. Deardorff WJ, Barnes DE, Jeon SY, Boscardin WJ, Langa KM, Covinsky KE, et al. Development and External Validation of a Mortality Prediction Model for Community-Dwelling Older Adults With Dementia. JAMA Intern Med. 2022; 182(11):1161-1170.
10. Tian Y, Li T, Zhang M, Wang R, Ma C, Guo Y, Wang R. Impact of the Time-Weighted Average Glucose Concentration and Diabetes on In-Hospital Mortality in Critically Ill Patients Older Than 75 Years: A Retrospective Cohort Study. Endocr Pract. 2022; 28(11):1152-1158.
11. Ijaz N, Buta B, Xue QL, Mohess DT, Bushan A, Tran H, et al. Interventions for Frailty Among Older Adults With Cardiovascular Disease: JACC State-of-the-Art Review. J Am Coll Cardiol. 2022; 79(5): p. 482-503.
12. Triposkiadis F, Xanthopoulos A, Butler J. Cardiovascular Aging and Heart Failure: JACC Review Topic of the Week. J Am Coll Cardiol. 2019; 74(6): p. 804-813.
13. Magidson PD. The Aged Heart. Emerg Med Clin North Am. 2022; 40(4): p. 637-649.
14. Triposkiadis F, Xanthopoulos A, Parissis J, Butler J, Farmakis D. Pathogenesis of chronic heart failure: cardiovascular aging, risk factors, comorbidities, and disease modifiers. Heart Fail Rev. 2022; 27(1): p. 337-344.

For peer review only - http://bmjopen.bmj.com/site/about/guidelines.xhtml

15. Peikert A, Martinez FA, Vaduganathan M, Claggett BL, Kulac IJ, Desai AS, et al. Efficacy and Safety of Dapagliflozin in Heart Failure With Mildly Reduced or Preserved Ejection Fraction According to Age: The DELIVER Trial. Circ Heart Fail. 2022; 15(10): e010080.
16. von Elm E, Altman DG, Egger M, Pocock SJ, Gøtzsche PC, Vandenbroucke JP. The Strengthening the Reporting of Observational Studies in Epidemiology (STROBE) statement: guidelines for reporting observational studies. Lancet. 2007; 370(9596): p. 1453-1457.
17. Duvekot MHC, Venema E, Rozeman AD, Moudrous W, Vermeij FH, Biekart M, et al. Comparison of eight prehospital stroke scales to detect intracranial large-vessel occlusion in suspected stroke (PRESTO): a prospective observational study. Lancet Neurol. 2021; 20(3): p. 213-221.
18. Martín-Rodríguez F, Sanz-García A, Ortega Moreno L, Del Pozo Vegas C, Castro-Villamor MA, Martín-Conty JL, et al. Risk for early death in acutely ill older adults attended by prehospital emergency medical services. Emergencias. 2020; 32(3): p. 117-184
19. Roth G, Mensah G, Johnson C, et al. Global Burden of Cardiovascular Diseases and Risk Factors, 1990–2019. J Am Coll Cardiol. 2020; 76:2982–3021.
20. Than, M.P., Flaws, D.F., Cullen, L. et al. Cardiac Risk Stratification Scoring Systems for Suspected Acute Coronary Syndromes in the Emergency Department. Curr Emerg Hosp Med Rep. 2013; 1:53–63.
21. Wibring K, Herlitz J, Christensson L, Lingman M, Bång A. Prehospital factors associated with an acute life-threatening condition in non-traumatic chest pain patients - A systematic review. Int J Cardiol. 2016; 219:373-9.
22. Lai PH, Lancet EA, Weiden MD, Webber MP, Zeig-Owens R, Hall CB, Prezant DJ. Characteristics Associated With Out-of-Hospital Cardiac Arrests and Resuscitations During the Novel Coronavirus Disease 2019 Pandemic in New York City. JAMA Cardiol. 2020; 5:1154-1163.
23. Martín-Rodríguez F, López-Izquierdo R, Sanz-García A, Del Pozo Vegas C, Ángel Castro Villamor M, Mayo-Iscar A, et al. Novel Prehospital Phenotypes and Outcomes in Adult-Patients with Acute Disease. J Med Syst. 2022; 46:45.
24. Enriquez de Salamanca Gambara R, Sanz-García A, Martín-Conty JL, Polonio-López B, Del Pozo Vegas C, Martín-Rodríguez F, López-Izquierdo R. Long-Term Mortality in Patients Transferred by Emergency Medical Services: Prospective Cohort Study. Prehosp Disaster Med. 2023; 38:352-359.
25 Martín-Rodríguez F, López-Izquierdo R, Castro Villamor MA, Del Pozo Vegas C, Delgado Benito MDP, Martínez Caballero CM, Priego Martínez V, et al. The Prognostic Value of Prehospital Blood Lactate Levels to Predict Early Mortality in Acute Cardiovascular Disease. Shock. 2020; 53(2):164-170
26. Beygui F, Castren M, Brunetti ND, et al. Pre-hospital management of patients with chest pain and/or dyspnoea of cardiac origin. A position paper of the Acute Cardiovascular Care Association (ACCA) of the ESC. European Heart Journal: Acute Cardiovascular Care. 2020; 9:59-81.
27. Torabi P, Rivasi G, Hamrefors V, Ungar A, Sutton R, Brignole M, Fedorowski A. Early and late-onset syncope: insight into mechanisms. Eur Heart J. 2022; 43:2116-2123.

For peer review only - http://bmjopen.bmj.com/site/about/guidelines.xhtml

28. Gómez-Doblas JJ, Muñiz J, Martin JJ, Rodríguez-Roca G, Lobos JM, Awamleh P, et al. Prevalence of atrial fibrillation in Spain. OFRECE study results. Rev Esp Cardiol. 2014; 67:259-69
29. Morris E, McCartney D, Lasserson D, Van den Bruel A, Fisher R, Hayward G. Point-of-care lactate testing for sepsis at presentation to health care: a systematic review of patient outcomes. Br J Gen Pract. 2017 Dec;67(665):e859-e870.

For peer review only

For peer review only - http://bmjopen.bmj.com/site/about/guidelines.xhtml

Tables and figures

Table 1. Demographic and clinical baseline variables

Variable	≤ 74 years	≥ 75 years	Standardized difference[b]	Odds ratio[c] (95%CI)	*p* value[d]
No. (%) with data[a]	867 (49.7)	877 (50.3)	N.A.	N.A.	N.A.
**Epidemiological variables**					
Sex, female	287 (33.1)	438 (49.9)	0.168	0.50 [0.41;0.60]	<0.001
Age, year	62 (52-69)	84 (79-88)	2.486	N.A	<0.001
Nursing homes	21 (2.4)	169 (19.3)	0.169	0.10 [0.06;0.16]	<0.001
**On-scene vital signs**					
RR, breaths/min	16 (14-19)	17 (14-23)	0.166	0.98 [0.97;0.99]	0.001
SpO2, %	97 (95-98)	96 (93-98)	-0.225	1.03 [1.02;1.04]	<0.001
SBP, mmHg	135 (112-153)	134 (112-157)	0.043	1.00 [1.00;1.00]	0.366
DBP, mmHg	80 (67-94)	73 (60-86)	-0.301	1.02 [1.01;1.02]	<0.001
HR, beats/min	80 (65-100)	78 (62-99)	-0.119	1.00 [1.00;1.01]	0.013
Temperature, °C	36 (35.9-36.5)	36 (35.8-36.5)	0.013	0.98 [0.87;1.11]	0.778
Glasgow coma scale, points	15 (15-15)	15 (15-15)	-0.019	1.01 [0.97;1.05]	0.691
Glucose, mg/dL	124 (105-151)	141 (116-183)	0.266	1.00 [0.99;1.00]	<0.001
Lactate, mmol/L	1.77 (1.1-2.82)	1.97 (1.19-3.1)	0.042	0.98 [0.94;1.02]	0.376
**Baseline cardiac rhythm**					
Sinus	487 (56.2)	338 (38.5)	-0.176	2.04 [1.69;2.47]	<0.001
Atrial fibrillation	127 (14.6)	293 (33.4)	0.187	0.34 [0.27;0.43]	<0.001
Atrial flutter	7 (0.8)	8 (0.9)	0.001	0.89 [0.30;2.52]	0.820
Atrial tachycardia	98 (11.3)	63 (7.2)	-0.041	1.64 [1.18;2.30]	0.003
Supraventricular tachycardia	23 (2.7)	12 (1.4)	-0.012	1.95 [0.98;4.10]	0.058
Ventricular tachycardia	22 (2.5)	4 (0.5)	-0.020	5.50 [2.08;19.3]	<0.001
Sinus bradycardia	62 (7.2)	57 (6.5)	-0.006	1.11 [0.76;1.61]	0.591
1° degree block	3 (0.3)	24 (2.7)	0.023	0.13 [0.03;0.37]	<0.001
2° block type I	2 (0.2)	2 (0.2)	-0.000	1.01 [0.11;9.73]	0.991
2° block type II	4 (0.5)	7 (0.8)	0.003	0.59 [0.15;2.00]	0.397
Complete block	16 (1.8)	24 (2.7)	0.008	0.67 [0.35;1.27]	0.219
Pacemaker	6 (0.7)	40 (4.6)	0.008	0.15 [0.06;0.33]	<0.001
Junctional	1 (0.1)	3 (0.3)	0.038	0.37 [0.01;3.16]	0.380
Idioventricular	3 (0.3)	0	-0.003	N.A	
Asystole	3 (0.3)	0	-0.003	N.A	
Ventricular fibrillation	3 (0.3)	2 (0.2)	-0.001	1.48 [0.23;12.8]	0.678
**Comorbidities**					
ACCI, points	1 (0-3)	3 (2-5)	0.557	0.79 [0.76;0.83]	<0.001
Congestive heart failure	109 (12.6)	279 (31.8)	0.192	0.31 [0.24;0.39]	<0.001
Myocardial infarction	237 (27.3)	321 (36.6)	0.092	0.65 [0.53;0.80]	<0.001
Peripheral vascular disease	97 (11.2)	137 (15.6)	0.044	0.68 [0.51;0.90]	0.007
Cerebrovascular disease	37 (4.3)	120 (13.7)	0.094	0.28 [0.19;0.41]	<0.001
Hemiplegia	23 (2.7)	48 (5.5)	0.028	0.47 [0.28;0.78]	0.003
Chronic pulmonary disease	189 (21.8)	199 (22.7)	0.008	0.95 [0.76;1.19]	0.655
DM uncomplicated	113 (13)	151 (17.2)	0.041	0.72 [0.55;0.94]	0.015
DM end organ damage	73 (8.4)	129 (14.7)	0.062	0.53 [0.39;0.72]	<0.001
Moderate-severe CKD	41 (4.7)	190 (21.7)	0.169	0.18 [0.13;0.25]	<0.001
Mild hepatic disease	30 (3.5)	27 (3.1)	-0.003	1.13 [0.66;1.93]	0.654
Severe hepatic disease	21 (2.4)	16 (1.8)	-0.006	1.33 [0.69;2.62]	0.387
Peptic ulcer disease	75 (8.7)	76 (8.7)	0.002	1.00 [0.71;1.40]	0.991
AIDS	4 (0.5)	1 (0.1)	-0.003	3.68 [0.51;101]	0.175
Lymphoma	7 (0.8)	16 (1.8)	0.010	0.44 [0.17;1.05]	0.063
Leukemia	13 (1.5)	13 (1.5)	-0.002	1.01 [0.46;2.23]	0.977
Metastatic solid tumor	20 (2.3)	33 (3.8)	0.014	0.61 [0.34;1.06]	0.077
Nonmetastatic solid tumor	119 (13.7)	209 (23.8)	0.101	0.51 [0.40;0.65]	<0.001

For peer review only - http://bmjopen.bmj.com/site/about/guidelines.xhtml

For peer review only

Connective tissue disease	48 (5.5)	49 (5.6)	0.001	0.99 [0.66;1.49]	0.963
Dementia	27 (3.1)	136 (15.5)	0.123	0.18 [0.11;0.27]	<0.001

Abbreviations: NA: Not applicable; Ref: reference; RR: respiratory rate; SPO2: oxygen saturation; SBP: systolic blood pressure; DBP: diastolic blood pressure; HR: heart rate; ACCI: Age-Charlson comorbidity index; DM: diabetes mellitus; CKP: chronic kidney disease; AIDS: acquired immunodeficiency syndrome.

[a]Values expressed as total number (percentage) and medians (25th-75th percentile), as appropriate.

[b]The Mann–Whitney U test or chi-squared test was used as appropriate.

cCohen's d-test was used to estimate the effect size.

[d]Fisher's exact probability statistic was used.

For peer review only

Table 2. Primary and key secondary outcomes

Variable	≤ 74 years	≥ 75 years	Standardized difference[b]	Odds ratio[c] (95%CI)	*p* value[d]
No. (%) with data[a]	867 (49.7)	877 (50.3)	N.A.	N.A.	N.A.
**Cumulative mortality**					
1-day	25 (2.9)	35 (4)	0.011	0.72 [0.42;1.20]	0.205
2-day	30 (3.5)	51 (5.8)	0.023	0.58 [0.36;0.92]	0.019
7-day	42 (4.8)	69 (7.9)	0.030	0.60 [0.40;0.88]	0.010
30-day	53 (6.1)	106 (12.1)	0.059	0.47 [0.33;0.67]	<0.001
90-day	74 (8.5)	156 (17.8)	0.092	0.43 [0.32;0.58]	<0.001
180-day	88 (10.1)	184 (21)	0.108	0.43 [0.32;0.56]	<0.001
365-day	101 (11.6)	229 (26.1)	0.144	0.37 [0.29;0.48]	<0.001
**Secondary outcome (support on-scene)**					
NIMV	22 (2.5)	65 (7.4)	0.048	0.33 [0.20;0.53]	<0.001
Mechanical ventilation	39 (4.5)	23 (2.6)	-0.018	1.74 [1.04;2.99]	0.034
Transcutaneous pacemaker	27 (3.1)	33 (3.8)	0.006	0.82 [0.49;1.38]	0.458
Cardioversion	22 (2.5)	11 (1.3)	-0.012	2.03 [1.00;4.41]	0.049
Defibrillation	24 (2.8)	8 (0.9)	-0.018	3.05 [1.41;7.35]	0.004
Vasoactive agents	31 (3.6)	29 (3.3)	-0.002		0.758
**Prehospital syndromic suspected condition**					
Ischemic heart disease	425 (49)	264 (30.1)	-0.189	0.45 [0.37;0.55]	<0.001
Acute heart failure	76 (8.8)	210 (23.9)	0.152	3.32 [2.51;4.43]	<0.001
Arrhythmia	112 (12.9)	116 (13.2)	0.001	1.02 [0.77;1.34]	0.961
Syncope	221 (25.2)	258 (29.4)	0.039	1.22 [0.99;1.50]	0.074
Hypertensive emergency	33 (3.8)	29 (3.3)	-0.005	0.86 [0.52;1.44]	0.664
**Hospital outcome**					
Inpatient	424 (48.9)	487 (55.5)	0.066	1.30 [1.08;1.58]	0.006
Hospitalization time, days	1 (0-6)	2 (0-7)	0.024	1.00 [0.99;1.01]	0.613
Echocardisocopy	335 (38.6)	303 (34.5)	-0.040	0.84 [0.69;1.02]	0.076
Fibrinolysis	26 (3)	10 (1.1)	-0.018	0.38 [0.17;0.77]	0.006
PIVS	249 (28.7)	157 (17.9)	-0.108	0.54 [0.43;0.68]	<0.001
Emergent surgery	21 (2.4)	18 (2.1)	-0.003	0.85 [0.44;1.60]	0.602
NIMV	26 (3)	70 (8)	0.049	2.79 [1.78;4.51]	<0.001
Mechanical ventilation	65 (7.5)	36 (4.1)	-0.033	0.53 [0.34;0.80]	<0.001
Vasoactive agents	63 (7.3)	55 (6.3)	-0.010	0.85 [0.59;1.24]	0.002
ACCU-admission	268 (30.9)	149 (19)	-0.139	0.46 [0.36;0.57]	<0.001

Abbreviations: NA: not applicable; Ref: reference; NIMV: noninvasive mechanical ventilation; PIVS: percutaneous interventional vascular surgery; ACCU: acute cardiac care unit.

[a]Values expressed as total number (percentage) and medians (25th-75th percentile), as appropriate.

[b]The Mann–Whitney U test or chi-squared test was used as appropriate.

cCohen's d-test was used to estimate the effect size.

[d]Fisher's exact probability statistic was used.

For peer review only - http://bmjopen.bmj.com/site/about/guidelines.xhtml

Table 3. Hazard ratio derived from Cox regression.

Variable	Hazard ratio	5% Confidence interval	95% Confidence interval	*p* value
Cohort ≥ 75 years	1.98	1.51	2.60	<0.001
Being in nursing home	1.42	1.07	1.89	0.014
Respiratory rate	1.02	1.01	1.04	<0.001
Systolic blood pressure	0.99	0.98	0.99	0.017
Lactate	1.25	1.20	1.30	<0.001
Noninvasive mechanical ventilation	1.83	1.26	2.65	0.001
Mechanical ventilation	2.53	1.30	4.92	0.006
Cardioversion	2.21	1.22	4.03	0.009
Defibrillation	2.39	1.23	4.64	0.010
Acute heart failure	2.32	1.63	3.31	<0.001
Age-Charlson comorbidity index	1.13	1.09	1.18	<0.001

For peer review only

For peer review only - http://bmjopen.bmj.com/site/about/guidelines.xhtml

Figure 1. Study population flowchart

Figure 2. Kaplan–Meier survival curves for age ≤ 74 years vs ≥ 75 years (A) and cardiovascular diagnosis for both groups together (B). Figure 1A, Age ≤ 74 years (red line) and age ≥ 75 years (blue line). Figure 1B, ischemic heart disease (red line), acute heart failure (dark green), arrhythmia (light green), syncope (blue line), hypertensive emergency (purple line).

For peer review only

For peer review only - http://bmjopen.bmj.com/site/about/guidelines.xhtml

Figure 1. Study population flowchart

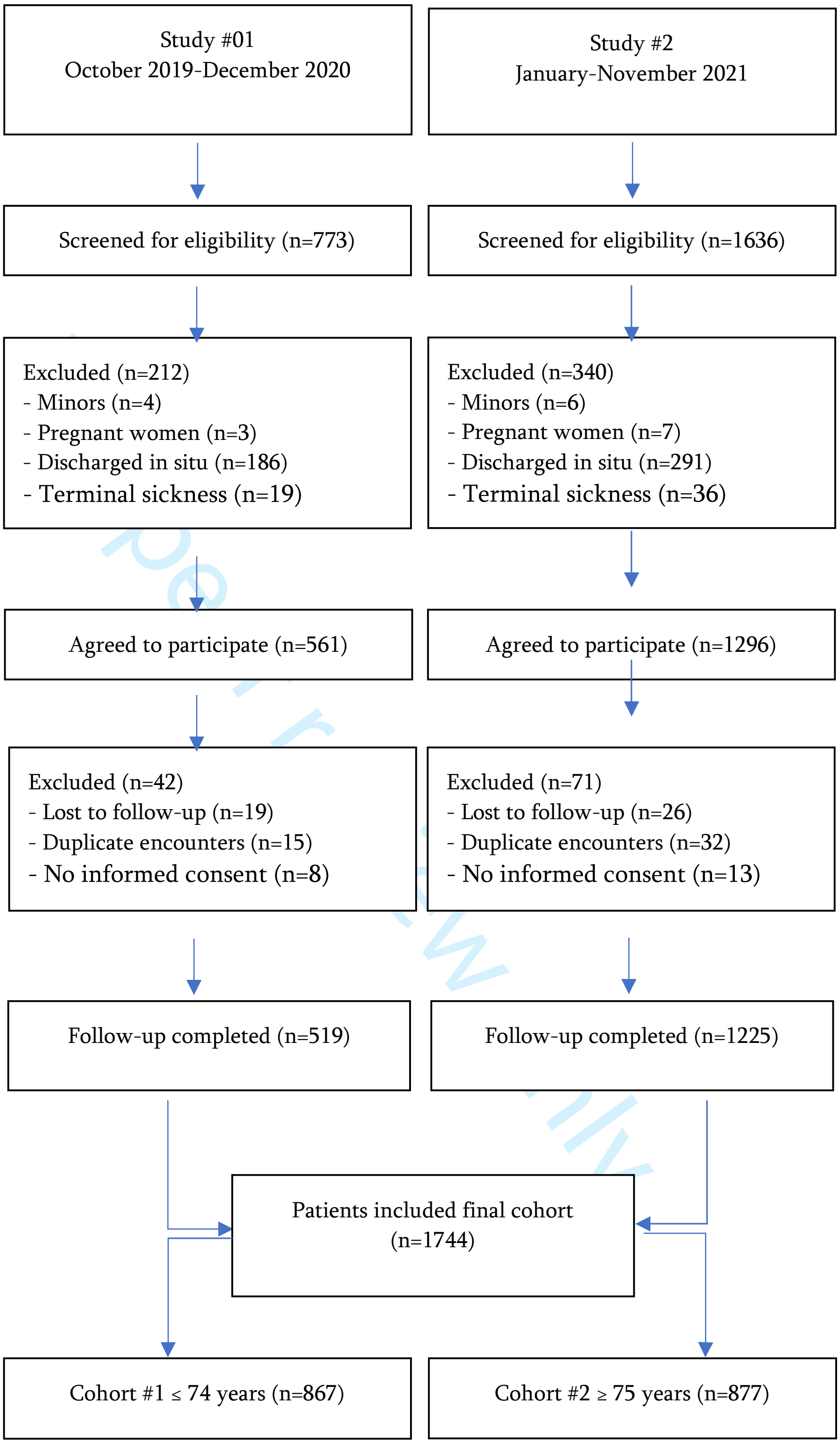

For peer review only - http://bmjopen.bmj.com/site/about/guidelines.xhtml

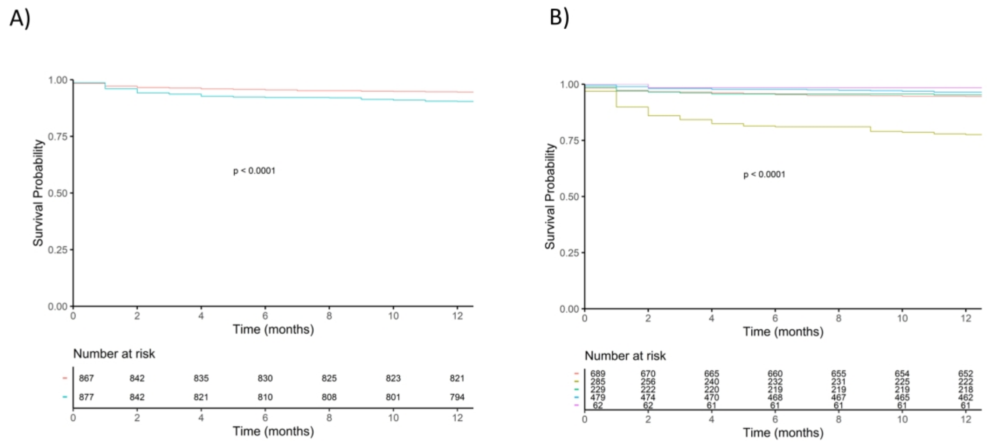

Figure 2. Kaplan–Meier survival curves for age ≤ 74 years vs ≥ 75 years (A) and cardiovascular diagnosis for both groups together (B). Figure 1A, Age ≤ 74 years (red line) and age ≥ 75 years (blue line). Figure 1B, ischemic heart disease (red line), acute heart failure (dark green), arrhythmia (light green), syncope (blue line), hypertensive emergency (purple line).

254x190mm (300 x 300 DPI)

Supplementary data

For peer review only

For peer review only - http://bmjopen.bmj.com/site/about/guidelines.xhtml

Table of contents

Supplementary methods…..……………………………………………………………..3
Strengthening the Reporting of Observational Studies in Epidemiology (STROBE) Statement…...……….....……………………………………….…3
Supplementary Results..………………………………………………………….……5
- Supplementary Figure 1. Kaplan Meier survival curves according to cardiovascular diagnosis and age…………………………………………………...5
- Supplementary Table S1. Cumulative mortality by prehospital syndromic suspect……………………………………………………………………………6
- Supplementary Table S2: Summary descriptives table by groups of acute heart failure...…...……………………………………………………………............7
- Supplementary Table S3: Summary descriptives table by groups of Arrythmmia...……………………………………………………………………8
- Supplementary Table S4: Summary descriptives table by groups of Hypertensive emergency…………………………….................................9
- Supplementary Table S5 Summary descriptives table by groups of ischemic heart disease…….……..…………………………………………………..10
- Supplementary Table S6: Summary descriptives table by groups of syncope..11
- Supplementary Table S7: Log-regression table by mortality groups…………12
References..14

For peer review only - http://bmjopen.bmj.com/site/about/guidelines.xhtml

Supplementary Methods

STROBE[1] Statement—checklist of items that should be included in reports of observational studies

	Item No	Recommendation	Page No
**Title and abstract**	1	(*a*) Indicate the study's design with a commonly used term in the title or the abstract	1
		(*b*) Provide in the abstract an informative and balanced summary of what was done and what was found	3
**Introduction**			
Background/rationale	2	Explain the scientific background and rationale for the investigation being reported	5
Objectives	3	State specific objectives, including any prespecified hypotheses	5
**Methods**			
Study design	4	Present key elements of study design early in the paper	6
Setting	5	Describe the setting, locations, and relevant dates, including periods of recruitment, exposure, follow-up, and data collection	6
Participants	6	(*a*) *Cohort study*—Give the eligibility criteria, and the sources and methods of selection of participants. Describe methods of follow-up<br>*Case-control study*—Give the eligibility criteria, and the sources and methods of case ascertainment and control selection. Give the rationale for the choice of cases and controls<br>*Cross-sectional study*—Give the eligibility criteria, and the sources and methods of selection of participants	6
		(*b*) *Cohort study*—For matched studies, give matching criteria and number of exposed and unexposed<br>*Case-control study*—For matched studies, give matching criteria and the number of controls per case	6
Variables	7	Clearly define all outcomes, exposures, predictors, potential confounders, and effect modifiers. Give diagnostic criteria, if applicable	7
Data sources/ measurement	8*	For each variable of interest, give sources of data and details of methods of assessment (measurement). Describe comparability of assessment methods if there is more than one group	7
Bias	9	Describe any efforts to address potential sources of bias	7
Study size	10	Explain how the study size was arrived at	7
Quantitative variables	11	Explain how quantitative variables were handled in the analyses. If applicable, describe which groupings were chosen and why	7
Statistical methods	12	(*a*) Describe all statistical methods, including those used to control for confounding	7
		(*b*) Describe any methods used to examine subgroups and interactions	7
		(*c*) Explain how missing data were addressed	7
		(*d*) *Cohort study*—If applicable, explain how loss to follow-up was addressed<br>*Case-control study*—If applicable, explain how matching of cases and controls was addressed<br>*Cross-sectional study*—If applicable, describe analytical methods taking account of sampling strategy	7
		(*e*) Describe any sensitivity analyses	7

Continued on next page

For peer review only - http://bmjopen.bmj.com/site/about/guidelines.xhtml

**Results**			
Participants	13*	(a) Report numbers of individuals at each stage of study—eg numbers potentially eligible, examined for eligibility, confirmed eligible, included in the study, completing follow-up, and analysed	8
		(b) Give reasons for non-participation at each stage	8
		(c) Consider use of a flow diagram	8
Descriptive data	14*	(a) Give characteristics of study participants (eg demographic, clinical, social) and information on exposures and potential confounders	8
		(b) Indicate number of participants with missing data for each variable of interest	8
		(c) *Cohort study*—Summarise follow-up time (eg, average and total amount)	8
Outcome data	15*	*Cohort study*—Report numbers of outcome events or summary measures over time	*8*
		Case-control study—Report numbers in each exposure category, or summary measures of exposure	*8*
		Cross-sectional study—Report numbers of outcome events or summary measures	*8*
Main results	16	(*a*) Give unadjusted estimates and, if applicable, confounder-adjusted estimates and their precision (eg, 95% confidence interval). Make clear which confounders were adjusted for and why they were included	9
		(*b*) Report category boundaries when continuous variables were categorized	9
		(*c*) If relevant, consider translating estimates of relative risk into absolute risk for a meaningful time period	9
Other analyses	17	Report other analyses done—eg analyses of subgroups and interactions, and sensitivity analyses	9
**Discussion**			
Key results	18	Summarise key results with reference to study objectives	10-11
Limitations	19	Discuss limitations of the study, taking into account sources of potential bias or imprecision. Discuss both direction and magnitude of any potential bias	11-12
Interpretation	20	Give a cautious overall interpretation of results considering objectives, limitations, multiplicity of analyses, results from similar studies, and other relevant evidence	10,11,12
Generalisability	21	Discuss the generalisability (external validity) of the study results	11,12,13
**Other information**			
Funding	22	Give the source of funding and the role of the funders for the present study and, if applicable, for the original study on which the present article is based	2

*Give information separately for cases and controls in case-control studies and, if applicable, for exposed and unexposed groups in cohort and cross-sectional studies.

Note: An Explanation and Elaboration article discusses each checklist item and gives methodological background and published examples of transparent reporting. The STROBE checklist is best used in conjunction with this article (freely available on the Web sites of PLoS Medicine at http://www.plosmedicine.org/, Annals of Internal Medicine at http://www.annals.org/, and Epidemiology at http://www.epidem.com/). Information on the STROBE Initiative is available at www.strobe-statement.org.

For peer review only - http://bmjopen.bmj.com/site/about/guidelines.xhtml

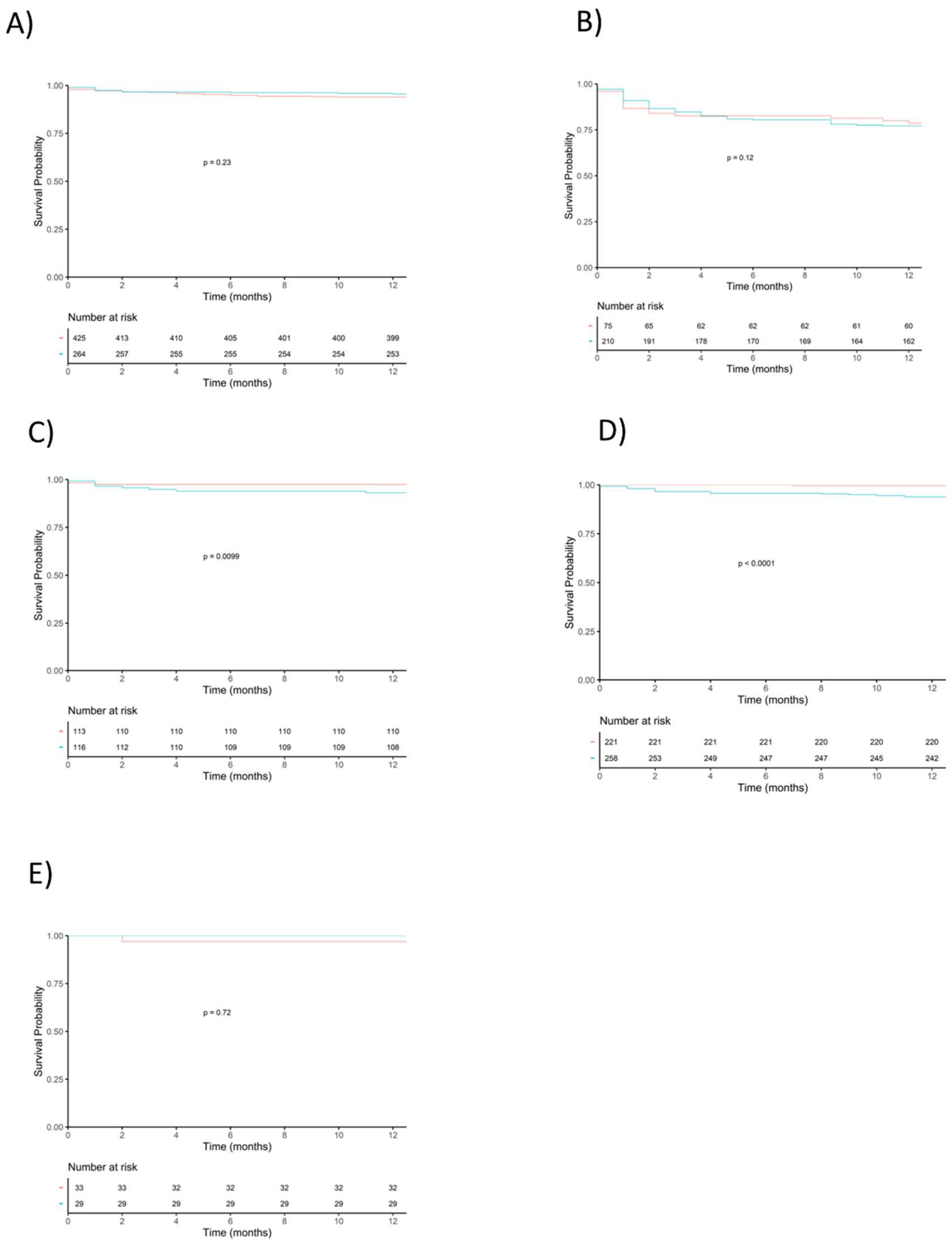

Supplementary Figure S1. Kaplan Meier survival curves for Age <75 years vs ≥ 75 years according to cardiovascular diagnosis. Ischemic heart disease (A), acute heart failure (B), arrhythmia (C), syncope (D), hypertensive emergency (E). Age ≤ 74 years (red line), and age ≥ 75 years (blue line).

Supplementary Table S1. Cumulative mortality by prehospital syndromic suspect

Variable[a]	Ischemic heart disease	Acute heart failure	Arrhythmia	Syncope	Hypertensive emergency	*p* value[b]
**≤ 74 years**	425 (49)	76 (8.8)	112 (12.9)	221 (25.2)	33 (3.8)	
1-day	12 (2.8)	10 (13.2)	3 (2.7)	0	0	0.022
2-day	14 (3.3)	12 (15.8)	3 (2.7)	0	1 (3)	0.033
7-day	24 (5.6)	13 (17.1)	3 (2.7)	1 (0.5)	1 (3)	0.002
30-day	26 (6.1)	21 (27.6)	3 (2.7)	2 (0.9)	1 (3)	0.002
90-day	33 (7.8)	26 (23.2)	7 (6.3)	7 (3.2)	1 (3)	0.012
180-day	38 (8.9)	28 (36.8)	9 (8)	10 (4.5)	3 (9.1)	0.060
365-day	43 (10.1)	33 (43.4)	10 (8.9)	12 (5.4)	3 (9.1)	0.042
**≥ 75 years**	264 (30.1)	210 (23.9)	116 (13.2)	258 (29.4)	29 (3.3)	
1-day	7 (2.7)	19 (9)	4 (3.4)	5 (1.9)	0	0.133
2-day	9 (3.4)	28 (13.3)	5 (4.3)	9 (3.5)	0	0.161
7-day	10 (3.8)	41 (19.5)	7 (6)	11 (4.3)	0	0.100
30-day	17 (6.4)	60 (28.6)	11 (9.5)	18 (7)	0	0.043
90-day	22 (8.3)	86 (41)	16 (13.8)	32 (12.4)	0	0.114
180-day	25 (9.5)	99 (47.1)	20 (17.2)	40 (15.5)	0	0.185
365-day	35 (13.3)	115 (54.8)	26 (22.4)	51 (19.8)	2 (6.9)	0.235

[a]Values expressed as total number (percentage).

[b]Fisher's Exact Probability statistic, was used.

For peer review only

Supplementary Table S2: Summary descriptives table by groups of acute heart failure

	Not Suffering acute heart failure<br>*N=75*	Suffering acute heart failure<br>*N=210*	OR	p.ratio	p.overall
1-day:					0.406
0	65 (86.7%)	191 (91.0%)	Ref.	Ref.	
1	10 (13.3%)	19 (9.05%)	0.64 [0.29;1.52]	0.304	
2-day:					0.706
0	63 (84.0%)	182 (86.7%)	Ref.	Ref.	
1	12 (16.0%)	28 (13.3%)	0.80 [0.39;1.74]	0.566	
7-day:					0.807
0	62 (82.7%)	169 (80.5%)	Ref.	Ref.	
1	13 (17.3%)	41 (19.5%)	1.15 [0.59;2.37]	0.693	
30-day:					1.000
0	54 (72.0%)	150 (71.4%)	Ref.	Ref.	
1	21 (28.0%)	60 (28.6%)	1.03 [0.57;1.88]	0.934	
90-day:					0.306
0	50 (66.7%)	124 (59.0%)	Ref.	Ref.	
1	25 (33.3%)	86 (41.0%)	1.38 [0.80;2.44]	0.249	
180-day:					0.125
0	48 (64.0%)	111 (52.9%)	Ref.	Ref.	
1	27 (36.0%)	99 (47.1%)	1.58 [0.92;2.75]	0.097	
365-day:					0.096
0	43 (57.3%)	95 (45.2%)	Ref.	Ref.	
1	32 (42.7%)	115 (54.8%)	1.62 [0.95;2.78]	0.074	

0: survival; 1: non-survival

Supplementary Table S3: Summary descriptives table by groups of Arrythmia

	Not Suffering Arrythmia N=113	Suffering Arrythmia N=116	OR	p.ratio	p.overall
1-day:					1.000
0	110 (97.3%)	112 (96.6%)	Ref.	Ref.	
1	3 (2.65%)	4 (3.45%)	1.29 [0.26;7.15]	0.748	
2-day:					0.722
0	110 (97.3%)	111 (95.7%)	Ref.	Ref.	
1	3 (2.65%)	5 (4.31%)	1.62 [0.37;8.55]	0.524	
7-day:					0.333
0	110 (97.3%)	109 (94.0%)	Ref.	Ref.	
1	3 (2.65%)	7 (6.03%)	2.28 [0.60;11.4]	0.233	
30-day:					0.060
0	110 (97.3%)	105 (90.5%)	Ref.	Ref.	
1	3 (2.65%)	11 (9.48%)	3.69 [1.10;17.5]	0.034	
90-day:					0.149
0	105 (92.9%)	100 (86.2%)	Ref.	Ref.	
1	8 (7.08%)	16 (13.8%)	2.07 [0.86;5.38]	0.103	
180-day:					0.092
0	103 (91.2%)	96 (82.8%)	Ref.	Ref.	
1	10 (8.85%)	20 (17.2%)	2.12 [0.96;4.99]	0.063	
365-day:					0.015
0	102 (90.3%)	90 (77.6%)	Ref.	Ref.	
1	11 (9.73%)	26 (22.4%)	2.65 [1.26;5.91]	0.010	

0: survival; 1: non-survival

Supplementary Table S4: Summary descriptives table by groups of Hypertensive Emergency

	**Not Suffering Hypertensive Emergency** *N=33*	**Suffering Hypertensive Emergency** *N=29*	**OR**	**p.ratio**	**p.overall**
1-day: 0	33 (100%)	29 (100%)	Ref.	Ref.	.
2-day:					1.000
0	32 (97.0%)	29 (100%)	Ref.	Ref.	
1	1 (3.03%)	0 (0.00%)	. [.;.]	.	
7-day:					1.000
0	32 (97.0%)	29 (100%)	Ref.	Ref.	
1	1 (3.03%)	0 (0.00%)	. [.;.]	.	
30-day:					1.000
0	32 (97.0%)	29 (100%)	Ref.	Ref.	
1	1 (3.03%)	0 (0.00%)	. [.;.]	.	
90-day:					1.000
0	32 (97.0%)	29 (100%)	Ref.	Ref.	
1	1 (3.03%)	0 (0.00%)	. [.;.]	.	
180-day:					0.241
0	30 (90.9%)	29 (100%)	Ref.	Ref.	
1	3 (9.09%)	0 (0.00%)	. [.;.]	.	
365-day:					1.000
0	30 (90.9%)	27 (93.1%)	Ref.	Ref.	
1	3 (9.09%)	2 (6.90%)	0.76 [0.08;5.36]	0.782	

0: survival; 1: non-survival

Supplementary Table S5: Summary descriptives table by groups of ischemic heart disease

	Not suffering ischemic heart disease N=425	Suffering ischemic heart disease N=264	OR	p.ratio	p.overall
1-day:					1.000
0	413 (97.2%)	257 (97.3%)	Ref.	Ref.	
1	12 (2.82%)	7 (2.65%)	0.95 [0.34;2.41]	0.910	
2-day:					1.000
0	411 (96.7%)	255 (96.6%)	Ref.	Ref.	
1	14 (3.29%)	9 (3.41%)	1.04 [0.42;2.43]	0.925	
7-day:					0.360
0	401 (94.4%)	254 (96.2%)	Ref.	Ref.	
1	24 (5.65%)	10 (3.79%)	0.66 [0.30;1.38]	0.280	
30-day:					0.994
0	399 (93.9%)	247 (93.6%)	Ref.	Ref.	
1	26 (6.12%)	17 (6.44%)	1.06 [0.55;1.98]	0.859	
90-day:					0.902
0	392 (92.2%)	242 (91.7%)	Ref.	Ref.	
1	33 (7.76%)	22 (8.33%)	1.08 [0.61;1.89]	0.785	
180-day:					0.922
0	387 (91.1%)	239 (90.5%)	Ref.	Ref.	
1	38 (8.94%)	25 (9.47%)	1.07 [0.62;1.81]	0.811	
365-day:					0.254
0	382 (89.9%)	229 (86.7%)	Ref.	Ref.	
1	43 (10.1%)	35 (13.3%)	1.36 [0.84;2.18]	0.211	

0: survival; 1: non-survival

Supplementary Table S6: Summary descriptives table by groups of syncope

	**Not suffering syncope** *N=221*	**Suffering syncope** *N=258*	**OR**	**p.ratio**	**p.overall**
1-day:					0.065
0	221 (100%)	253 (98.1%)	Ref.	Ref.	
1	0 (0.00%)	5 (1.94%)	. [.;.]	.	
2-day:					0.004
0	221 (100%)	249 (96.5%)	Ref.	Ref.	
1	0 (0.00%)	9 (3.49%)	. [.;.]	.	
7-day:					0.018
0	220 (99.5%)	247 (95.7%)	Ref.	Ref.	
1	1 (0.45%)	11 (4.26%)	8.65 [1.65;214]	0.007	
30-day:					0.002
0	219 (99.1%)	240 (93.0%)	Ref.	Ref.	
1	2 (0.90%)	18 (6.98%)	7.67 [2.16;52.7]	0.001	
90-day:					<0.001
0	214 (96.8%)	226 (87.6%)	Ref.	Ref.	
1	7 (3.17%)	32 (12.4%)	4.24 [1.93;10.8]	<0.001	
180-day:					<0.001
0	211 (95.5%)	218 (84.5%)	Ref.	Ref.	
1	10 (4.52%)	40 (15.5%)	3.82 [1.93;8.31]	<0.001	
365-day:					<0.001
0	209 (94.6%)	207 (80.2%)	Ref.	Ref.	
1	12 (5.43%)	51 (19.8%)	4.24 [2.26;8.58]	<0.001	

0: survival; 1: non-survival

For peer review only - http://bmjopen.bmj.com/site/about/guidelines.xhtml

Supplementary Table S7: Log-regression table by mortality groups

	Survival<br>*N=1414*	Non-Survival<br>*N=330*	Hazard Ratio [95% Confidence interval]	p.value
Cohort:				
≤ 74 years	766 (54.2%)	101 (30.6%)	Ref.	Ref.
≥ 75 years	648 (45.8%)	229 (69.4%)	2.41 [1.91;3.04]	<0.001
Sex:				
Male	805 (56.9%)	214 (64.8%)	Ref.	Ref.
Female	609 (43.1%)	116 (35.2%)	0.74 [0.59;0.93]	0.010
Nursing home				
No	1297 (91.7%)	257 (77.9%)	Ref.	Ref.
Yes	117 (8.27%)	73 (22.1%)	2.69 [2.07;3.49]	<0.001
Respiratory Rate	17.9 (6.35)	22.1 (10.5)	1.06 [1.05;1.07]	<0.001
SpO2	95.7 (5.23)	87.2 (13.0)	0.94 [0.93;0.94]	<0.001
SBP	138 (32.7)	123 (40.8)	0.99 [0.98;0.99]	<0.001
DBP	79.0 (19.1)	69.6 (24.5)	0.98 [0.97;0.98]	<0.001
Heart rate	83.5 (31.8)	95.1 (42.9)	1.01 [1.01;1.01]	<0.001
Temperature	36.1 (0.68)	36.1 (0.98)	0.94 [0.81;1.10]	0.424
GCS	14.8 (1.25)	12.8 (4.00)	0.79 [0.77;0.81]	<0.001
Glucose	141 (57.3)	191 (90.8)	1.01 [1.00;1.01]	<0.001
Lactate	1.99 (1.35)	4.63 (3.79)	1.37 [1.33;1.40]	<0.001
NIMV:				
No	1387 (98.1%)	270 (81.8%)	Ref.	Ref.
Yes	27 (1.91%)	60 (18.2%)	6.85 [5.17;9.08]	<0.001
Mechanical ventilation:				
No	1404 (99.3%)	278 (84.2%)	Ref.	Ref.
Yes	10 (0.71%)	52 (15.8%)	14.5 [10.7;19.6]	<0.001
Transcutaneous pacemaker:				

	Survival	Non-Survival	Hazard Ratio [95% Confidence interval]	p.value
	N=1414	*N=330*		
No	1364 (96.5%)	320 (97.0%)	Ref.	Ref.
Yes	50 (3.54%)	10 (3.03%)	0.91 [0.48;1.70]	0.759
Cardioversion:				
No	1396 (98.7%)	315 (95.5%)	Ref.	Ref.
Yes	18 (1.27%)	15 (4.55%)	3.24 [1.93;5.43]	<0.001
Defibrillation.:				
No	1406 (99.4%)	306 (92.7%)	Ref.	Ref.
Yes	8 (0.57%)	24 (7.27%)	9.04 [5.95;13.7]	<0.001
Vasoactive agents:				
No	1402 (99.2%)	282 (85.5%)	Ref.	Ref.
Yes	12 (0.85%)	48 (14.5%)	13.4 [9.81;18.2]	<0.001
Prehospital syndromic suspect:				
Ischemic heart disease	611 (43.2%)	78 (23.6%)	Ref.	Ref.
Acute heart failure	138 (9.76%)	147 (44.5%)	6.01 [4.57;7.92]	<0.001
Arrhythmia	192 (13.6%)	37 (11.2%)	1.46 [0.98;2.15]	0.060
Syncope	416 (29.4%)	63 (19.1%)	1.16 [0.83;1.61]	0.394
Hypertensive emergency	57 (4.03%)	5 (1.52%)	0.68 [0.28;1.69]	0.412
ACCI	2.31 (2.33)	4.29 (2.96)	1.23 [1.19;1.27]	<0.001

References

1. Vandenbroucke JP, von Elm E, Altman DG, Gøtzsche PC, Mulrow CD, Pocock SJ, Poole C, Schlesselman JJ, Egger M; STROBE Initiative. Strengthening the Reporting of Observational Studies in Epidemiology (STROBE): explanation and elaboration. PLoS Med. 2007 Oct 16;4(10):e297.

For peer review only

For peer review only - http://bmjopen.bmj.com/site/about/guidelines.xhtml

STROBE[1] Statement—checklist of items that should be included in reports of observational studies

	Item No	Recommendation	Page No
**Title and abstract**	1	(*a*) Indicate the study's design with a commonly used term in the title or the abstract	1
		(*b*) Provide in the abstract an informative and balanced summary of what was done and what was found	3
**Introduction**			
Background/rationale	2	Explain the scientific background and rationale for the investigation being reported	5
Objectives	3	State specific objectives, including any prespecified hypotheses	5
**Methods**			
Study design	4	Present key elements of study design early in the paper	6
Setting	5	Describe the setting, locations, and relevant dates, including periods of recruitment, exposure, follow-up, and data collection	6
Participants	6	(*a*) *Cohort study*—Give the eligibility criteria, and the sources and methods of selection of participants. Describe methods of follow-up<br>*Case-control study*—Give the eligibility criteria, and the sources and methods of case ascertainment and control selection. Give the rationale for the choice of cases and controls<br>*Cross-sectional study*—Give the eligibility criteria, and the sources and methods of selection of participants	6
		(*b*) *Cohort study*—For matched studies, give matching criteria and number of exposed and unexposed<br>*Case-control study*—For matched studies, give matching criteria and the number of controls per case	6
Variables	7	Clearly define all outcomes, exposures, predictors, potential confounders, and effect modifiers. Give diagnostic criteria, if applicable	7
Data sources/ measurement	8*	For each variable of interest, give sources of data and details of methods of assessment (measurement). Describe comparability of assessment methods if there is more than one group	7
Bias	9	Describe any efforts to address potential sources of bias	7
Study size	10	Explain how the study size was arrived at	7
Quantitative variables	11	Explain how quantitative variables were handled in the analyses. If applicable, describe which groupings were chosen and why	7
Statistical methods	12	(*a*) Describe all statistical methods, including those used to control for confounding	7
		(*b*) Describe any methods used to examine subgroups and interactions	7
		(*c*) Explain how missing data were addressed	7
		(*d*) *Cohort study*—If applicable, explain how loss to follow-up was addressed<br>*Case-control study*—If applicable, explain how matching of cases and controls was addressed<br>*Cross-sectional study*—If applicable, describe analytical methods taking account of sampling strategy	7
		(*e*) Describe any sensitivity analyses	7

Continued on next page

For peer review only - http://bmjopen.bmj.com/site/about/guidelines.xhtml

**Results**			
Participants	13*	(a) Report numbers of individuals at each stage of study—eg numbers potentially eligible, examined for eligibility, confirmed eligible, included in the study, completing follow-up, and analysed	8
		(b) Give reasons for non-participation at each stage	8
		(c) Consider use of a flow diagram	8
Descriptive data	14*	(a) Give characteristics of study participants (eg demographic, clinical, social) and information on exposures and potential confounders	8
		(b) Indicate number of participants with missing data for each variable of interest	8
		(c) *Cohort study*—Summarise follow-up time (eg, average and total amount)	8
Outcome data	15*	*Cohort study*—Report numbers of outcome events or summary measures over time	*8*
		Case-control study—Report numbers in each exposure category, or summary measures of exposure	*8*
		Cross-sectional study—Report numbers of outcome events or summary measures	*8*
Main results	16	(*a*) Give unadjusted estimates and, if applicable, confounder-adjusted estimates and their precision (eg, 95% confidence interval). Make clear which confounders were adjusted for and why they were included	9
		(*b*) Report category boundaries when continuous variables were categorized	9
		(*c*) If relevant, consider translating estimates of relative risk into absolute risk for a meaningful time period	9
Other analyses	17	Report other analyses done—eg analyses of subgroups and interactions, and sensitivity analyses	9
**Discussion**			
Key results	18	Summarise key results with reference to study objectives	10-11
Limitations	19	Discuss limitations of the study, taking into account sources of potential bias or imprecision. Discuss both direction and magnitude of any potential bias	11-12
Interpretation	20	Give a cautious overall interpretation of results considering objectives, limitations, multiplicity of analyses, results from similar studies, and other relevant evidence	10,11,12
Generalisability	21	Discuss the generalisability (external validity) of the study results	11,12,13
**Other information**			
Funding	22	Give the source of funding and the role of the funders for the present study and, if applicable, for the original study on which the present article is based	2

*Give information separately for cases and controls in case-control studies and, if applicable, for exposed and unexposed groups in cohort and cross-sectional studies.

Note: An Explanation and Elaboration article discusses each checklist item and gives methodological background and published examples of transparent reporting. The STROBE checklist is best used in conjunction with this article (freely available on the Web sites of PLoS Medicine at http://www.plosmedicine.org/, Annals of Internal Medicine at http://www.annals.org/, and Epidemiology at http://www.epidem.com/). Information on the STROBE Initiative is available at www.strobe-statement.org.

For peer review only

BMJ Open

Prehospital acute life-threatening cardiovascular disease in elderly: An observational, prospective, multicenter, ambulance-based cohort study.

Journal:	*BMJ Open*
Manuscript ID	bmjopen-2023-078815.R2
Article Type:	Original research
Date Submitted by the Author:	06-Nov-2023
Complete List of Authors:	del Pozo Vegas, Carlos; Hospital Clinico Universitario de Valladolid, Emergency Department; Universidad de Valladolid<br>Zalama-Sánchez, Daniel; Hospital Clinico Universitario de Valladolid, Emergency Department<br>Sanz-Garcia, Ancor; University of Castilla-La Mancha - Center for University Studies Talavera de la Reina<br>López-Izquierdo, Raúl; Hosp Univ Rio Hortega<br>Sáez-Belloso, Silvia; Universidad de Valladolid<br>Oleaga, Cristina Mazas Perez; Universidad Europea del Atlántico; Universidad Internacional Iberoamericana<br>Azpíroz, Irma Domínguez; Universidad Europea del Atlántico; Universidad Internacional Iberoamericana<br>Pascual, Iñaki Elío; Universidad Europea del Atlántico; Universidade Internacional do Cuanza Cuito<br>Martín-Rodríguez, Francisco; Universidad de Valladolid
**Primary Subject Heading**:	Emergency medicine
Secondary Subject Heading:	Cardiovascular medicine
Keywords:	ACCIDENT & EMERGENCY MEDICINE, Aged, 80 and over, CARDIOLOGY

SCHOLARONE™
Manuscripts

I, the Submitting Author has the right to grant and does grant on behalf of all authors of the Work (as defined in the below author licence), an exclusive licence and/or a non-exclusive licence for contributions from authors who are: i) UK Crown employees; ii) where BMJ has agreed a CC-BY licence shall apply, and/or iii) in accordance with the terms applicable for US Federal Government officers or employees acting as part of their official duties; on a worldwide, perpetual, irrevocable, royalty-free basis to BMJ Publishing Group Ltd ("BMJ") its licensees and where the relevant Journal is co-owned by BMJ to the co-owners of the Journal, to publish the Work in this journal and any other BMJ products and to exploit all rights, as set out in our licence.

The Submitting Author accepts and understands that any supply made under these terms is made by BMJ to the Submitting Author unless you are acting as an employee on behalf of your employer or a postgraduate student of an affiliated institution which is paying any applicable article publishing charge ("APC") for Open Access articles. Where the Submitting Author wishes to make the Work available on an Open Access basis (and intends to pay the relevant APC), the terms of reuse of such Open Access shall be governed by a Creative Commons licence – details of these licences and which Creative Commons licence will apply to this Work are set out in our licence referred to above.

Other than as permitted in any relevant BMJ Author's Self Archiving Policies, I confirm this Work has not been accepted for publication elsewhere, is not being considered for publication elsewhere and does not duplicate material already published. I confirm all authors consent to publication of this Work and authorise the granting of this licence.

For peer review only

Prehospital acute life-threatening cardiovascular disease in elderly: An observational, prospective, multicenter, ambulance-based cohort study.

Carlos del Pozo Vegas[1,2], Daniel Zalama-Sánchez[1], Ancor Sanz-García[3,*], Raúl López-Izquierdo[2,4,5], Silvia Sáez-Belloso [6,7], Cristina Mazas Pérez-Oleaga[8,9,10]; Irma Domínguez Azpíroz[8,11,12]; Iñaki Elío Pascual[8,12,13]; Francisco Martín-Rodríguez[2,6]

[1] Emergency Department. Hospital Clínico Universitario. Valladolid, Spain.

[2] Faculty of Medicine. Universidad de Valladolid, Valladolid, Spain.

[3] Faculty of Health Sciences, Universidad de Castilla la Mancha, Talavera de la Reina, Spain.

[4] Emergency Department. Hospital Universitario Rio Hortega. Valladolid, Spain

[5] CIBER of Respiratory Diseases, Instituto de Salud Carlos III, C/Sinesio Delgado, 4 28029, Madrid, Spain.

[6] Advanced Life Support, Emergency Medical Services (SACYL), Valladolid, Spain.

[7] Faculty of Nursing. Universidad de Valladolid, Valladolid, Spain.

[8] Universidad Europea del Atlántico. Isabel Torres 21 39011 Santander, Spain.

[9] Universidad Internacional Iberoamericana. Arecibo, Puerto Rico 00613, USA.

[10] Universidad de La Romana. La Romana, República Dominicana

[11] Universidad Internacional Iberoamericana. Campeche 24560, México.

[12] Universidade Internacional do Cuanza. Cuito, Bié, Angola

[13] Fundación Universitaria Internacional de Colombia. Bogotá, Colombia

[*] *Corresponding author*

Corresponding author:

Dr. Ancor Sanz-García
Faculty of Health Sciences, Universidad de Castilla la Mancha. Avda. Real Fábrica de Seda, s/n 45600, Talavera de la Reina, Toledo
e-mail: ancor.sanz@gmail.com
Phone: 925 721 010

Running head

Prehospital cardiovascular disease in elderly

For peer review only - http://bmjopen.bmj.com/site/about/guidelines.xhtml

Abstract

Objective

The aim was to explore the association of demographic and prehospital parameters with short- and long-term mortality in acute life-threatening cardiovascular disease by using a hazard model, focusing on elderly individuals, by comparing patients under 75 years versus patients over 75 years of age.

Design

Prospective, multicenter, observational study.

Setting

Emergency medical services (EMS) delivery study gathering data from two back-to-back studies between October 1, 2019, and November 30, 2021. Six advanced life support (ALS), forty-three basic life support (BLS), and five hospitals in Spain were considered.

Participants

Adult patients suffering from acute life-threatening cardiovascular disease attended by the EMS.

Primary and secondary outcome measures

The primary outcome was in-hospital mortality from any cause within the first to the 365 days following EMS attendance. The main measures included prehospital demographics, biochemical variables, prehospital advanced life support techniques used, and syndromic suspected conditions.

Results

A total of 1744 patients fulfilled the inclusion criteria. The 365-day cumulative mortality in the elderly amounted to 26.1% (229 cases) vs 11.6% (11.6%) in patients under 75 years old. Elderly patients (≥ 75 years) presented a 2-fold risk of mortality compared to patients ≤ 74 years. Life-threatening interventions (mechanical ventilation, cardioversion and defibrillation) were also related to a 2-fold increased risk of mortality. Importantly, patients suffering from acute heart failure presented a more than 2-fold increased risk of mortality.

Conclusions

This study revealed the prehospital variables associated with the long-term mortality of patients suffering from acute cardiovascular disease. Our results provide important insights for the development of specific codes or scores for cardiovascular diseases to facilitate the risk of mortality characterization.

For peer review only - http://bmjopen.bmj.com/site/about/guidelines.xhtml

For peer review only

Keywords Prehospital; Emergency Medical Services; elderly; cardiovascular disease; mortality.

Strengths and limitations of this study

-Here, we present a prospective, multicenter, observational study.

-We explored all adult patients suffering from acute life-threatening cardiovascular disease attended by the EMS.

-We present a relevant sample size with a reduced loss-to-follow-up rate.

-As an observational study this could entail a selection bias.

-Some doubts could arise with prehospital symptoms due to the lack of complementary tests.

For peer review only

Introduction

Cardiovascular diseases represent the leading cause of prehospital care, involving a surprising number of unplanned hospitalizations and sudden unexplained mortality (1). Emergency medical services (EMS) must handle this overwhelming patient workload quickly and efficiently, following clinical guidelines and under recognized training, e.g., basic and advanced cardiac life support (BCLS and ACLS) (2) (3).

The setup and implementation of specific detection codes for life-threatening conditions, e.g., cardiorespiratory arrest, ST-elevation coronary syndrome, and stroke, is a well-established procedure in health systems, and EMS plays an active role in detection, emergency critical care, and assisted transfer to a suitable hospital (4). Predefined standard protocols should be applied in diseases with clear symptomatology or well-defined guiding symptoms, and in general, no major operational problems are encountered. The handicap for EMS providers consists of the identification on-scene or *en* route of patients with acute life-threatening cardiovascular disease that is apparently masked. Early warning scores and point-of-care testing can provide an effective support tool to help at critical junctures in the complicated decision-making process (5) (6). However, the identification of high-risk subjects is challenging, particularly when distracting factors such as comorbidities or age come into play (7).

EMS has increasingly switched types of patients, with older adults becoming a major focus area of care (8). Defining the concept of older adults is complex, as there are different categories and timelines, and there is no standard criterion to say that an older person is considered elderly (9). However, there is a general acceptance that persons over 65 years of age are considered elderly, and persons over 75 years of age are classified as late elderly. On the other hand, life expectancy and aging control have improved considerably, with age-related comorbidities usually appearing later, which is the main reason why the 75-year age limit has been selected to differentiate the cohorts of the present work (10). Among other reasons, falls, drug-taking mistakes, and exacerbations of chronic pathologies are more frequent in elderly individuals, i.e., EMS providers can attend to cases of older adults with comorbidities and multimedication. In particular, cardiovascular diseases constitute the first cause of emergency appointments and inpatient hospitalization in older adults, affecting both

For peer review only - http://bmjopen.bmj.com/site/about/guidelines.xhtml

males and females (11). Life expectancy has increased significantly, meaning that older adults with atrial fibrillation, acute coronary syndrome, congestive heart failure, valvular heart disease, and other cardiovascular processes are becoming increasingly prevalent (12). Complications related to aging can hamper anamnesis and clinical examination and sometimes disrupt responsiveness mechanisms, e.g., 20% of older adults exhibit atypical symptoms of acute coronary syndrome (13). Additionally, coexisting comorbidities may trigger interactions in the cardiovascular system, such as anemia, chronic kidney disease, or diabetes (14). Comorbidities naturally rise sharply with age and necessitate an appropriate analysis. Nonetheless, at 75 years old, cardiovascular pathology in particular showed considerable progression with significant increases in comorbidities, the emergence of additional diseases and the exacerbation of already prevalent pathologies. The group of elderly individuals over 75 years old constitutes a cluster of special follow-ups that could be especially worthwhile for an in-depth characterization (10) (15).

The purpose of the study was to explore the association of demographic and prehospital parameters with short- and long-term mortality in acute life-threatening cardiovascular disease by using a hazard model. In particular, we focused on the elderly by comparing two age cohorts: under 75 years versus over 75 years.

For peer review only

For peer review only - http://bmjopen.bmj.com/site/about/guidelines.xhtml

Methods

Study design

This prospective, multicenter, observational, EMS delivery analysis gathers inputs obtained from two back-to-back studies, “Prehospital identification of prognostic biomarkers in time-dependent diseases” (HITScore) and “Identification of biomarkers of clinical-risk deterioration in prehospital care” (preBIOs).

Ethical approval was granted by the Rio Hortega University Hospital Ethics Committee, references PI041-19 and PI217-20 (principal investigator's reference center. This authorization applies to the other locations covered by the study). This study was reported according to STrengthening the Reporting of OBservational studies in Epidemiology (Supplementary data p3) (16) and complies with the Declaration of Helsinki.

Study setting

The study was conducted in four Spanish provinces (Burgos, Salamanca, Segovia, and Valladolid), enrolling uninterrupted adults (>18 years) with syndromic cardiovascular suspects who were transferred by ambulance to the Emergency Department (ED) between October 1, 2019, and November 30, 2021. Global community medical care was provided by the Public Health System (SACYL) and included the Emergency Coordination Center (1-1-2 phone backup), six advanced life support (ALS), forty-three basic life support (BLS), and five hospitals (one minor general district hospital and four university tertiary hospitals). Normally, BLS is staffed by two emergency medical technicians (EMTs), performing on-scene or *en route* BCLS work-up protocols, and ALS is made up of two EMTs, an emergency registered nurse (ERN) and a physician, conducting ACLS operations.

All cases were examined by an ALS, and following the assessment and diagnostic tests, the physician determined, in line with current guidelines and according to the individual clinical situation, the need for transfer to the ED, as well as the type of ambulance: BLS or ALS. All hospitals presented the Acute Cardiac Care Unit (ACCU), and three hospitals presented the cardiac intervention room 24/7 and emergency cardiac surgery unit. Patients who needed emergency hemodynamic studies or advanced cardiologic care and for whom these facilities were not available at the reference hospital were evacuated as top priority (daytime by Helicopter Emergency Medical Service and nighttime by ALS, mandatory with turnaround times under 1 hour) to other hospitals included in the study.

Population

For peer review only - http://bmjopen.bmj.com/site/about/guidelines.xhtml

Recruitment was consecutive. Participants enrolled in the study were defined as adults (>18 years) with acute cardiovascular disease (prehospital syndromic suspected condition) managed by EMS and transferred to the ED. Noncardiovascular disorders, minors, pregnant women (known or apparent), terminally ill patients (condition confirmed by a medical report), or on-site discharge were excluded.

An informed consent form, managed by the ERN and applicable for the entire follow-up study, was reviewed and countersigned by all participants. In the absence of appropriate understanding by the patient, a research associate in the ED tried to collect the consent form signed by the patient or by a family member or legal guardian. If, despite all efforts, consent was not obtained, the patient was removed and excluded from the study.

Data collection

Mandatory on-site hands-on training was conducted for all staff before the study started and included the standardized procedure for taking vital signs, handling, calibration, and cleaning of the point-of-care testing device, as well as data input to a database specially created for this purpose. A specific database was designed, with access by individual passwords and double authentication. In this database, we entered both the data collected from the EMS medical records and later the data from hospital care and subsequent follow-up by reviewing the electronic medical records (in two steps, at 30 and 365 days). Once the data had been linked, the data manager anonymized the patient identifiers.

Age, sex, nursing home location, and on-scene vital signs (respiratory rate, oxygen saturation, systolic and diastolic blood pressure, heart rate, temperature, Glasgow Coma Scale, glucose, lactate, and electrocardiogram) were collected and recorded by the ERN. A LifePAK® 15 monitor-defibrillator (Physio-Control, Inc., Redmond, USA) was applied to obtain oxygen saturation, blood pressure, heart rate and electrocardiogram. A ThermoScan® PRO 6000 thermometer (Welch Allyn, Inc., Skaneateles Falls, USA) was used to collect temperature, and finally, the analyser epoc® (Siemens Healthcare GmbH, Erlangen Germany) was employed to perform prehospital analysis.

The physician subsequently checked the electrocardiogram and recorded the baseline heart rhythm, as well as the 17 comorbidity categories needed to calculate the Age-Charlson comorbidity index (ACCI), composed of myocardial infarction, congestive heart failure, peripheral vascular disease, stroke or transient ischemic attack, dementia, chronic obstructive pulmonary disease, connective tissue disease peptic ulcer disease, mild liver disease,

For peer review only - http://bmjopen.bmj.com/site/about/guidelines.xhtml

uncomplicated diabetes mellitus, hemiplegia, moderate to severe chronic kidney disease, diabetes mellitus with end-organ damage, localized solid tumor, leukemia, lymphoma, moderate to severe liver disease, metastatic solid tumor, and acquired immunodeficiency syndrome.

Outcomes

The primary outcome was cumulative mortality, from prehospital care to one year of follow-up, segregating time periods as follows: 1, 2, 7, 30, 90, 180, and 365 days. In addition, a comparison was performed matching two age-typed cohorts, a group of ≤ 74 years vs a group of ≥ 75 years, with age discrimination in line with similar reports (17) (18).

The secondary prehospital outcomes included advanced airway management (noninvasive or invasive mechanical ventilation), electrical therapy (transcutaneous pacemaker, cardioversion, or defibrillation) and/or vasoactive agents. Finally, the ALS physician appointed the prehospital syndromic suspected condition, involving ischemic heart disease, acute heart failure, arrhythmia, syncope, or hypertensive emergency.

The secondary hospital outcomes were collected from the electronic medical records obtained at the one-year follow-up and comprised cumulative mortality (all-cause), admission rate, echocardisocopy, percutaneous interventional vascular surgery, emergent surgery, advanced airway management, vasoactive agents, and ACCU admission.

Data analyses

Descriptive results and the associations between age and cardiovascular diagnosis with the variables were assessed by the Mann–Whitney U test or the chi-squared test, when appropriate, and the effect size in the form of standardized mean difference was provided. Absolute values and percentages were used for categorical variables, and median interquartile ranges (IQRs) were used for continuous variables because they did not follow a normal distribution. The procedure to determine those variables associated with mortality was as follows: First, a log-rank univariate analysis was performed. Then, a Cox regression (which included only those variables with $p<0.001$ in the log-rank univariate analysis) was performed to evaluate the association of demographic and prehospital parameters with mortality. The Cox regression results were expressed as the hazard ratio and 95% confidence interval (CI). Furthermore, survival according to age, cardiovascular diagnosis, and the combination of both variables was obtained using the Kaplan–Meier method (KM). The cumulative mortality by prehospital syndromic suspect and the difference between groups were assessed by the chi-

For peer review only - http://bmjopen.bmj.com/site/about/guidelines.xhtml

squared test. Finally, the descriptive statistics and association of mortality at 1, 2, 7, 30, 90, 180 and 365 days for each prehospital syndromic suspect were assessed by univariate comparison and expressed as odds ratios and 95% CIs.

Data for prehospital covariates were prospectively collected and registered in a database generated with IBM SPSS Statistics for Apple version 20.0 software. (IBM Corp, Armonk USA). The caseload entry system was tested to delete unclear or ambiguous items and to verify the adequacy of the data-gathering system. The data present missing values completely at random; therefore, the strategy used (listwise deletion) does not imply biased means, variances or regression weights. The statistical power (from 1 to 100) of the present study is 89.4 based on the following considerations: (i) the sample used is n = 517, (ii) significant level of p = 0.05, (iii) expected odds ratios (OR) of 0.405, and (iv) 14% of events.

Patient and Public Involvement

No patient involved

For peer review only

Results

Among 2409 cases, 1744 patients with a syndromic cardiovascular suspect managed by EMS and transferred to the ED were finally included in the final analysis. There were 867 patients from cohort #1 (≤ 74 years) and 877 from cohort #2 (≥ 75 years), 49.7% vs 50.3% (figure 1). Cohort #1 was characterized by a median age of 62 years (IQR: 52-69), and 33.1% (287 cases) were females. Half of the cases (56.2%) showed sinus rhythm, and the comorbidity burden resulted in an ACCI of 1 point (IQR: 0-3), especially myocardial infarction (27.3%), chronic pulmonary disease (21.8%) and nonmetastatic solid tumors (13.7%). In contrast, cohort #2 was described by a median age of 84 years (IQR: 79-88), 49.9% (438 cases) were female, and one-fifth lived in nursing homes. Despite sinus rhythm being the most common baseline cardiac rhythm (38.5%), the ratio of atrial fibrillation (33.4%) is noteworthy. The ACCI score was significantly higher, with a median of 3 points (IQR: 2-5) and a very pronounced prevalence of myocardial infarction (36.6%), congestive heart failure (31.8%), and nonmetastatic solid tumors (23.8%) (table 1).

The 365-day cumulative mortality in the elderly amounted to 26.1% (229 cases) vs 11.6% (11.6%) in patients under 75 years old. Cohort #1 presented a significantly increased percentage of mechanical ventilation, defibrillation, cardioversion, percutaneous interventional vascular surgery, and ACCU admission vs cohort #2, with a relatively increased incidence of noninvasive mechanical ventilation and transcutaneous pacemakers. Concerning prehospital syndromic suspected conditions, half of the patients under 75 years old presented with ischemic heart disease (49%), followed by syncope (25.2%). The elderly also showed ischemic heart disease (30.1%) and syncope (29.4%), stressing in particular the elevated incidence of acute heart failure (23.9% vs. 8.8%, respectively, intercohort) (table 2). Cumulative mortality by prehospital syndromic suspected condition is reported in Supplementary Table S1, as only those patients under 75 years presented statistically significant differences between the prehospital syndromic suspected conditions. The OR of each prehospital syndromic suspected condition for each mortality interval can be found in Supplementary Tables S2 to S6.

The Cox regression (Table 3) that included all those variables with p<0.001 in the long-rank analysis (supplementary table S7) showed that being elderly, being in a nursing home, high respiratory rate, low systolic blood pressure, high levels of lactate, use of noninvasive mechanical ventilation, mechanical ventilation, cardioversion defibrillation, suffering from

For peer review only - http://bmjopen.bmj.com/site/about/guidelines.xhtml

acute heart failure and an elevated Age-Charlson comorbidity index were variables statistically significantly associated with mortality. The aforementioned results for elderly individuals and those with acute heart failure are illustrated in Figure 2. Figure 2A shows the survival curve of patients ≤ 74 years vs ≥ 75 years, with statistically significant differences ($p<0.001$) that appeared at the beginning of the follow-up and remained stable over time. Figure 2B shows the mortality curves for each prehospital syndromic suspected condition, with acute heart failure presenting the highest mortality that started at the beginning of the follow-up and remained stable over time. When analysing the results according to age, those patients suffering from arrhythmia and syncope presented statistically significant differences in the mortality curves (Supplementary Figure S1) but not the other conditions. This was also the case when considering the association of each particular condition with mortality at 1, 2, 7, 30, 90, 180 and 365 days (Supplementary Tables S2 to S6); only arrhythmia and syncope presented statistically significant time points, arrhythmia only at 365-day mortality, and syncope at all time points except for 1-day mortality.

For peer review only - http://bmjopen.bmj.com/site/about/guidelines.xhtml

Discussion

This prospective, multicenter, observational, EMS delivery study assessed the association of prehospital variables, particularly elderly variables, with short- and long-term mortality in patients with acute life-threatening cardiovascular diseases. Elderly patients (≥ 75 years) presented a 2-fold risk of mortality compared to patients ≤ 74 years. Life-threatening interventions (mechanical ventilation, cardioversion, and defibrillation) were also related to a 2-fold increased risk of mortality. Importantly, patients suffering from acute heart failure presented a more than 2-fold increased risk of mortality.

Cardiovascular diseases are the leading cause of EMS attendance (1) and are associated with high morbidity and mortality (19). Therefore, the accurate and quick characterization of patients at the first contact could provide critical information for the development of specialized risk scores (20) or specific detection codes (4). In this sense, the present study aims to describe the association of prehospital variables with mortality, particularly in elderly individuals, which is one of the main risk factors for cardiovascular diseases (21). Our results align with this evidence, since the categorization of patients ≤ 74 years and ≥ 75 years revealed that older patients presented a higher rate of mortality. Moreover, other factors related to elderly individuals, such as being in nursing homes or the number of comorbidities, measured by the ACCI, were risk factors for mortality. As expected, the higher the number of comorbidities, the higher the risk of mortality. This is not surprising since several comorbidities worsen cardiovascular conditions (14). In fact, comorbidities make patient examination difficult, which is particularly true in the prehospital setting (22). Strikingly, the leap to worse long-term mortality outcomes occurs above 75 years old, whereas below this age, short-term mortality outcomes take prominence.

Despite all this evidence, the effect of age on mortality was not found for short-term mortality, and no statistically significant effect was found for one- or two-day mortality; instead, the statistical significance was relevant at 30 days onwards. This short-term mortality result is somewhat surprising, since it has been previously described at the prehospital level that older ages are associated with higher short-term mortality (23). Perhaps this difference could be explained by the fact that those patients were characterized as suffering mainly from respiratory diseases (23). The long-term mortality relationship with age has already been described in the prehospital setting (24).

For peer review only - http://bmjopen.bmj.com/site/about/guidelines.xhtml

Lactate was statistically related to long-term mortality. The lactate value as a predictor of mortality is well documented in prehospital critical care and represents a very powerful indicator of mitochondrial hypoperfusion, directly affecting the production of bioavailable energy for all physiological processes, including the cardiovascular system (25). This has also been reported, as for respiratory rate and age, for long-term mortality when dealing with all patients and not with disease-specific analysis (24). Enriquez de Salamanca Gambara et al. (24) also found that GCS and SpO2 were associated with mortality. Perhaps GCS was more related to neurological diseases than cardiovascular diseases, which is our case. A similar argument could be made for SpO2, which could be related to respiratory diseases.

Life-threatening interventions are closely related to patients suffering from cardiovascular disease (26), which was also our case, since noninvasive mechanical ventilation, mechanical ventilation, cardioversion, and defibrillation were procedures present in the nonsurvivor group. Patients who must undergo advanced life support interventions on scene or en route, even overcoming the situation that originated the life-threatening intervention, are also negatively impacted in the long term. In other words, in patients needed to receive more aggressive manoeuvres, clinical evolution should be considered, even more so in elderly and frail patients.

Particularly relevant is the fact that the mortality of each prehospital syndromic suspected condition was different when considering elderly individuals. As expected, acute heart failure was the main condition related to death; however, when analysing mortality according to age groups, only arrhythmia and syncope presented statistically significant differences. This should be interpreted as the higher the age, the higher the probability of death, but only for those two conditions. The other conditions should be treated independently of age. The results for syncope and arrhythmia could be explained by the increased incidence with age, particularly at 70 years for syncope (27) and arrythmias, especially for atrial fibrillation (28).

Our study has some strengths, including the sample size, novelty, and study design with a reduced loss-to-follow-up rate. It was conducted in both rural and urban areas, and our results could be generalizable to other health systems. This last point is based on the fact that all parameters associated with mortality can be easily accessed by the EMS staff. Only lactate determination will require a point-of-care device, which is now a reality in several EMSs (29). This generalizability could lead to scores or specific detection codes. However, some limitations must be considered. First, this is an observational study, so we cannot rule out the

For peer review only - http://bmjopen.bmj.com/site/about/guidelines.xhtml

possibility of selection bias, although participating centers had previous experience and enrolled consecutive patients with prehospital acute cardiovascular disease. To obtain a representative sampling, cases were collected 24/7/365 nonstop in urban, semiurban and rural areas and in different ambulance stations. Second, possible bias may exist in relation to case inclusion in the study. All prehospital acute cardiovascular conditions were included; however, certain disorders may raise uncertainty, i.e., stomachache could be labelled a digestive disease at prehospital care, although following complementary in-hospital tests (imaging studies, laboratory tests, etc.), the disease ends up being categorized as ischemic heart disease. Nevertheless, the limitation was dampened for two reasons. On-scene, ALS physicians could issue up to a maximum of three diagnostic suspicions, and therefore, any case with a diagnosis of acute cardiovascular disease was eventually included in the analysis. In addition, the diagnostic coincidence between prehospital syndromic cardiovascular suspect (ischemic heart disease, acute heart failure, arrhythmia, syncope, and hypertensive emergency) and the final in-hospital diagnosis was very consistently strong. Third, data extractors were not blinded. To avoid cross-contamination, EMS providers lacked access to hospital follow-up data, and hospital investigators remained blinded to prehospital care data. To accurately link the data between prehospital care and hospital follow-up, at least 5 of the following extractors had to be an exact match: health system ID card, incident reference (EMS-register), first and last name, age, sex at birth, date, and/or time of arrival at the ED. Total access to the master database was given exclusively by the principal investigator and data manager. Fourth, modifiable lifestyles (smoking, alcohol consumption, weight, diet quality and physical activity) or medication history are indeed associated with short- and long-term mortality in life-threatening acute cardiovascular disease. Nevertheless, these data could not be collected in prehospital care or during the in-hospital follow-up phase. For subsequent investigations, a postindex event interview procedure will be implemented to collect these data and thus be able to analyse the influence of these covariates on the final outcome. Finally, an adequate sample size was used in the present preliminary study, but multicenter studies in different health systems are needed to confirm the generalizability of the results.

In summary, this study revealed the prehospital variables associated with the long-term mortality of patients suffering from acute cardiovascular disease. Elderly age, life-threatening interventions, acute heart failure, comorbidities, and lactate should be considered when EMS

For peer review only - http://bmjopen.bmj.com/site/about/guidelines.xhtml

patients have acute life-threatening cardiovascular diseases. In particular, the effect on elderly patients presenting arrhythmia or syncope should be considered. Finally, our results could pave the way for the development of specific codes or scores for cardiovascular diseases to facilitate the risk of mortality characterization.

For peer review only

For peer review only - http://bmjopen.bmj.com/site/about/guidelines.xhtml

Authors' Contributions

CRediT authorship contribution statement:

Francisco Martín-Rodríguez conceptualized the project, managed and coordinated the project, assisted with the design of methodology, analysed data, and prepared the initial and final drafts of the manuscript. Ancor Sanz-García took responsibility for the data and their analysis. Carlos del Pozo Vegas, Daniel Zalama-Sánchez, Silvia Sáez-Belloso, and Raúl López Izquierdo assisted with the management and coordination of the project, assisted with the design of the methodology, and helped review the manuscript. All authors performed a critical review and approved the final manuscript for interpretation of the data and important intellectual input.

Summarized conflict of interest statements

All signing authors meet the requirements of authorship and have declared the nonexistence of potential conflicts of interest. Daniel Zalama-Sánchez, Ancor Sanz-García, Carlos del Pozo Vegas, Raúl López-Izquierdo, Silvia Sáez-Belloso, and Francisco Martín-Rodríguez report no conflicts of interest.

The authors have no disclosures to make.

On behalf of the other authors, the corresponding author guarantees the accuracy, transparency, and honesty of the data and information contained in the study, that no relevant information has been omitted and that all discrepancies between authors have been adequately resolved and described.

Funding information

This work was supported by the Gerencia Regional de Salud, Public Health System of Castilla y León (Spain) [grant numbers GRS 1903/A/19 and GRS 2131/A/20].

Sponsor role: none

Ethics approval

Ethical approval was granted by the Rio Hortega University Hospital Ethics Committee, references PI041-19 and PI217-20 (principal investigator's reference center. This authorization applies to the other locations covered by the study).

Notation of prior abstract publication/presentation

For peer review only

This article is an original work, has not been published before and is not being considered for publication elsewhere in its final form, in either printed or electronic media. It is not based on any previous communication to a society or meeting.

Data Availability

The data that support the findings of this study are available on request from the corresponding author ASG. The data are not publicly available due to restrictions, and their information could compromise the privacy of research participants.

For peer review only

For peer review only - http://bmjopen.bmj.com/site/about/guidelines.xhtml

References

1. Townsend N, Kazakiewicz D, Lucy Wright F, Timmis A, Huculeci R, Torbica A, et al. Epidemiology of cardiovascular disease in Europe. Nat Rev Cardiol. 2022; 19(2): p. 133-143.
2. Perkins GD, Graesner JT, Semeraro F, Olasveengen T, Soar J, Lott C, et al. European Resuscitation Council Guidelines 2021: Executive summary. Resuscitation. 2021; 161: p. 1-60.
3. Wyckoff MH, Singletary EM, Soar J, Olasveengen TM, Greif R, Liley HG, et al. 2021 International Consensus on Cardiopulmonary Resuscitation and Emergency Cardiovascular Care Science With Treatment Recommendations: Summary From the Basic Life Support; Advanced Life Support; Neonatal Life Support; Education, Implementation, and Teams. Circulation. 2022; 145(9): p. e645-e721.
4. Andersson HB, Seth M, Gurm HS, Bates ER. Pre-hospital Transport Times and Outcomes After Different Reperfusion Strategies for ST-Elevation Myocardial Infarction. Am J Cardiol. 2019; 123(3): p. 375-381.
5. Pirneskoski J, Lääperi M, Kuisma M, Olkkola KT, Nurmi J. Ability of prehospital NEWS to predict 1-day and 7-day mortality is reduced in older adult patients. Emerg Med J. 2021; 38(12): p. 913-918.
6. Martín-Rodríguez F, López-Izquierdo R, Castro Villamor MA, Del Pozo Vegas C, Delgado Benito MDP, Martínez Caballero CM, et al. The Prognostic Value of Prehospital Blood Lactate Levels to Predict Early Mortality in Acute Cardiovascular Disease. Shock. 2020; 53(2): p. 164-170.
7. Forman DE, Maurer MS, Boyd C, Brindis R, Salive ME, Horne FM, et al. Multimorbidity in Older Adults With Cardiovascular Disease. J Am Coll Cardiol. 2018; 71(19): p. 2149-2161.
8. Probst MA, Gibson T, Weiss RE, Yagapen AN, Malveau SE, Adler DH, et al. Risk Stratification of Older Adults Who Present to the Emergency Department With Syncope: The FAINT Score. Ann Emerg Med. 2020; 75(2): p. 147-158.
9. Deardorff WJ, Barnes DE, Jeon SY, Boscardin WJ, Langa KM, Covinsky KE, et al. Development and External Validation of a Mortality Prediction Model for Community-Dwelling Older Adults With Dementia. JAMA Intern Med. 2022; 182(11):1161-1170.
10. Tian Y, Li T, Zhang M, Wang R, Ma C, Guo Y, Wang R. Impact of the Time-Weighted Average Glucose Concentration and Diabetes on In-Hospital Mortality in Critically Ill Patients Older Than 75 Years: A Retrospective Cohort Study. Endocr Pract. 2022; 28(11):1152-1158.
11. Ijaz N, Buta B, Xue QL, Mohess DT, Bushan A, Tran H, et al. Interventions for Frailty Among Older Adults With Cardiovascular Disease: JACC State-of-the-Art Review. J Am Coll Cardiol. 2022; 79(5): p. 482-503.
12. Triposkiadis F, Xanthopoulos A, Butler J. Cardiovascular Aging and Heart Failure: JACC Review Topic of the Week. J Am Coll Cardiol. 2019; 74(6): p. 804-813.
13. Magidson PD. The Aged Heart. Emerg Med Clin North Am. 2022; 40(4): p. 637-649.
14. Triposkiadis F, Xanthopoulos A, Parissis J, Butler J, Farmakis D. Pathogenesis of chronic heart failure: cardiovascular aging, risk factors, comorbidities, and disease modifiers. Heart Fail Rev. 2022; 27(1): p. 337-344.

For peer review only - http://bmjopen.bmj.com/site/about/guidelines.xhtml

15. Peikert A, Martinez FA, Vaduganathan M, Claggett BL, Kulac IJ, Desai AS, et al. Efficacy and Safety of Dapagliflozin in Heart Failure With Mildly Reduced or Preserved Ejection Fraction According to Age: The DELIVER Trial. Circ Heart Fail. 2022; 15(10): e010080.
16. von Elm E, Altman DG, Egger M, Pocock SJ, Gøtzsche PC, Vandenbroucke JP. The Strengthening the Reporting of Observational Studies in Epidemiology (STROBE) statement: guidelines for reporting observational studies. Lancet. 2007; 370(9596): p. 1453-1457.
17. Duvekot MHC, Venema E, Rozeman AD, Moudrous W, Vermeij FH, Biekart M, et al. Comparison of eight prehospital stroke scales to detect intracranial large-vessel occlusion in suspected stroke (PRESTO): a prospective observational study. Lancet Neurol. 2021; 20(3): p. 213-221.
18. Martín-Rodríguez F, Sanz-García A, Ortega Moreno L, Del Pozo Vegas C, Castro-Villamor MA, Martín-Conty JL, et al. Risk for early death in acutely ill older adults attended by prehospital emergency medical services. Emergencias. 2020; 32(3): p. 117-184
19. Roth G, Mensah G, Johnson C, et al. Global Burden of Cardiovascular Diseases and Risk Factors, 1990–2019. J Am Coll Cardiol. 2020; 76:2982–3021.
20. Than, M.P., Flaws, D.F., Cullen, L. et al. Cardiac Risk Stratification Scoring Systems for Suspected Acute Coronary Syndromes in the Emergency Department. Curr Emerg Hosp Med Rep. 2013; 1:53–63.
21. Wibring K, Herlitz J, Christensson L, Lingman M, Bång A. Prehospital factors associated with an acute life-threatening condition in non-traumatic chest pain patients - A systematic review. Int J Cardiol. 2016; 219:373-9.
22. Lai PH, Lancet EA, Weiden MD, Webber MP, Zeig-Owens R, Hall CB, Prezant DJ. Characteristics Associated With Out-of-Hospital Cardiac Arrests and Resuscitations During the Novel Coronavirus Disease 2019 Pandemic in New York City. JAMA Cardiol. 2020; 5:1154-1163.
23. Martín-Rodríguez F, López-Izquierdo R, Sanz-García A, Del Pozo Vegas C, Ángel Castro Villamor M, Mayo-Iscar A, et al. Novel Prehospital Phenotypes and Outcomes in Adult-Patients with Acute Disease. J Med Syst. 2022; 46:45.
24. Enriquez de Salamanca Gambara R, Sanz-García A, Martín-Conty JL, Polonio-López B, Del Pozo Vegas C, Martín-Rodríguez F, López-Izquierdo R. Long-Term Mortality in Patients Transferred by Emergency Medical Services: Prospective Cohort Study. Prehosp Disaster Med. 2023; 38:352-359.
25 Martín-Rodríguez F, López-Izquierdo R, Castro Villamor MA, Del Pozo Vegas C, Delgado Benito MDP, Martínez Caballero CM, Priego Martínez V, et al. The Prognostic Value of Prehospital Blood Lactate Levels to Predict Early Mortality in Acute Cardiovascular Disease. Shock. 2020; 53(2):164-170
26. Beygui F, Castren M, Brunetti ND, et al. Pre-hospital management of patients with chest pain and/or dyspnoea of cardiac origin. A position paper of the Acute Cardiovascular Care Association (ACCA) of the ESC. European Heart Journal: Acute Cardiovascular Care. 2020; 9:59-81.
27. Torabi P, Rivasi G, Hamrefors V, Ungar A, Sutton R, Brignole M, Fedorowski A. Early and late-onset syncope: insight into mechanisms. Eur Heart J. 2022; 43:2116-2123.

For peer review only - http://bmjopen.bmj.com/site/about/guidelines.xhtml

28. Gómez-Doblas JJ, Muñiz J, Martin JJ, Rodríguez-Roca G, Lobos JM, Awamleh P, et al. Prevalence of atrial fibrillation in Spain. OFRECE study results. Rev Esp Cardiol. 2014; 67:259-69

29. Morris E, McCartney D, Lasserson D, Van den Bruel A, Fisher R, Hayward G. Point-of-care lactate testing for sepsis at presentation to health care: a systematic review of patient outcomes. Br J Gen Pract. 2017 Dec;67(665):e859-e870.

For peer review only

For peer review only - http://bmjopen.bmj.com/site/about/guidelines.xhtml

Tables and figures

Table 1. Demographic and clinical baseline variables

Variable	≤ 74 years	≥ 75 years	Standardized difference[b]	Odds ratio[c] (95%CI)	*p* value[d]
No. (%) with data[a]	867 (49.7)	877 (50.3)	N.A.	N.A.	N.A.
**Epidemiological variables**					
Sex, female	287 (33.1)	438 (49.9)	0.168	0.50 [0.41;0.60]	<0.001
Age, year	62 (52-69)	84 (79-88)	2.486	N.A	<0.001
Nursing homes	21 (2.4)	169 (19.3)	0.169	0.10 [0.06;0.16]	<0.001
**On-scene vital signs**					
RR, breaths/min	16 (14-19)	17 (14-23)	0.166	0.98 [0.97;0.99]	0.001
SpO2, %	97 (95-98)	96 (93-98)	-0.225	1.03 [1.02;1.04]	<0.001
SBP, mmHg	135 (112-153)	134 (112-157)	0.043	1.00 [1.00;1.00]	0.366
DBP, mmHg	80 (67-94)	73 (60-86)	-0.301	1.02 [1.01;1.02]	<0.001
HR, beats/min	80 (65-100)	78 (62-99)	-0.119	1.00 [1.00;1.01]	0.013
Temperature, °C	36 (35.9-36.5)	36 (35.8-36.5)	0.013	0.98 [0.87;1.11]	0.778
Glasgow coma scale, points	15 (15-15)	15 (15-15)	-0.019	1.01 [0.97;1.05]	0.691
Glucose, mg/dL	124 (105-151)	141 (116-183)	0.266	1.00 [0.99;1.00]	<0.001
Lactate, mmol/L	1.77 (1.1-2.82)	1.97 (1.19-3.1)	0.042	0.98 [0.94;1.02]	0.376
**Baseline cardiac rhythm**					
Sinus	487 (56.2)	338 (38.5)	-0.176	2.04 [1.69;2.47]	<0.001
Atrial fibrillation	127 (14.6)	293 (33.4)	0.187	0.34 [0.27;0.43]	<0.001
Atrial flutter	7 (0.8)	8 (0.9)	0.001	0.89 [0.30;2.52]	0.820
Atrial tachycardia	98 (11.3)	63 (7.2)	-0.041	1.64 [1.18;2.30]	0.003
Supraventricular tachycardia	23 (2.7)	12 (1.4)	-0.012	1.95 [0.98;4.10]	0.058
Ventricular tachycardia	22 (2.5)	4 (0.5)	-0.020	5.50 [2.08;19.3]	<0.001
Sinus bradycardia	62 (7.2)	57 (6.5)	-0.006	1.11 [0.76;1.61]	0.591
1° degree block	3 (0.3)	24 (2.7)	0.023	0.13 [0.03;0.37]	<0.001
2° block type I	2 (0.2)	2 (0.2)	-0.000	1.01 [0.11;9.73]	0.991
2° block type II	4 (0.5)	7 (0.8)	0.003	0.59 [0.15;2.00]	0.397
Complete block	16 (1.8)	24 (2.7)	0.008	0.67 [0.35;1.27]	0.219
Pacemaker	6 (0.7)	40 (4.6)	0.008	0.15 [0.06;0.33]	<0.001
Junctional	1 (0.1)	3 (0.3)	0.038	0.37 [0.01;3.16]	0.380
Idioventricular	3 (0.3)	0	-0.003	N.A	
Asystole	3 (0.3)	0	-0.003	N.A	
Ventricular fibrillation	3 (0.3)	2 (0.2)	-0.001	1.48 [0.23;12.8]	0.678
**Comorbidities**					
ACCI, points	1 (0-3)	3 (2-5)	0.557	0.79 [0.76;0.83]	<0.001
Congestive heart failure	109 (12.6)	279 (31.8)	0.192	0.31 [0.24;0.39]	<0.001
Myocardial infarction	237 (27.3)	321 (36.6)	0.092	0.65 [0.53;0.80]	<0.001
Peripheral vascular disease	97 (11.2)	137 (15.6)	0.044	0.68 [0.51;0.90]	0.007
Cerebrovascular disease	37 (4.3)	120 (13.7)	0.094	0.28 [0.19;0.41]	<0.001
Hemiplegia	23 (2.7)	48 (5.5)	0.028	0.47 [0.28;0.78]	0.003
Chronic pulmonary disease	189 (21.8)	199 (22.7)	0.008	0.95 [0.76;1.19]	0.655
DM uncomplicated	113 (13)	151 (17.2)	0.041	0.72 [0.55;0.94]	0.015
DM end organ damage	73 (8.4)	129 (14.7)	0.062	0.53 [0.39;0.72]	<0.001
Moderate-severe CKD	41 (4.7)	190 (21.7)	0.169	0.18 [0.13;0.25]	<0.001
Mild hepatic disease	30 (3.5)	27 (3.1)	-0.003	1.13 [0.66;1.93]	0.654
Severe hepatic disease	21 (2.4)	16 (1.8)	-0.006	1.33 [0.69;2.62]	0.387
Peptic ulcer disease	75 (8.7)	76 (8.7)	0.002	1.00 [0.71;1.40]	0.991
AIDS	4 (0.5)	1 (0.1)	-0.003	3.68 [0.51;101]	0.175
Lymphoma	7 (0.8)	16 (1.8)	0.010	0.44 [0.17;1.05]	0.063
Leukemia	13 (1.5)	13 (1.5)	-0.002	1.01 [0.46;2.23]	0.977
Metastatic solid tumor	20 (2.3)	33 (3.8)	0.014	0.61 [0.34;1.06]	0.077
Nonmetastatic solid tumor	119 (13.7)	209 (23.8)	0.101	0.51 [0.40;0.65]	<0.001

For peer review only - http://bmjopen.bmj.com/site/about/guidelines.xhtml

For peer review only

Connective tissue disease	48 (5.5)	49 (5.6)	0.001	0.99 [0.66;1.49]	0.963
Dementia	27 (3.1)	136 (15.5)	0.123	0.18 [0.11;0.27]	<0.001

Abbreviations: NA: Not applicable; Ref: reference; RR: respiratory rate; SPO2: oxygen saturation; SBP: systolic blood pressure; DBP: diastolic blood pressure; HR: heart rate; ACCI: Age-Charlson comorbidity index; DM: diabetes mellitus; CKP: chronic kidney disease; AIDS: acquired immunodeficiency syndrome.

[a]Values expressed as total number (percentage) and medians (25th-75th percentile), as appropriate.

[b]The Mann–Whitney U test or chi-squared test was used as appropriate.

cCohen's d-test was used to estimate the effect size.

[d]Fisher's exact probability statistic was used.

For peer review only

Table 2. Primary and key secondary outcomes

Variable	≤ 74 years	≥ 75 years	Standardized difference[b]	Odds ratio[c] (95%CI)	*p* value[d]
No. (%) with data[a]	867 (49.7)	877 (50.3)	N.A.	N.A.	N.A.
**Cumulative mortality**					
1-day	25 (2.9)	35 (4)	0.011	0.72 [0.42;1.20]	0.205
2-day	30 (3.5)	51 (5.8)	0.023	0.58 [0.36;0.92]	0.019
7-day	42 (4.8)	69 (7.9)	0.030	0.60 [0.40;0.88]	0.010
30-day	53 (6.1)	106 (12.1)	0.059	0.47 [0.33;0.67]	<0.001
90-day	74 (8.5)	156 (17.8)	0.092	0.43 [0.32;0.58]	<0.001
180-day	88 (10.1)	184 (21)	0.108	0.43 [0.32;0.56]	<0.001
365-day	101 (11.6)	229 (26.1)	0.144	0.37 [0.29;0.48]	<0.001
**Secondary outcome (support on-scene)**					
NIMV	22 (2.5)	65 (7.4)	0.048	0.33 [0.20;0.53]	<0.001
Mechanical ventilation	39 (4.5)	23 (2.6)	-0.018	1.74 [1.04;2.99]	0.034
Transcutaneous pacemaker	27 (3.1)	33 (3.8)	0.006	0.82 [0.49;1.38]	0.458
Cardioversion	22 (2.5)	11 (1.3)	-0.012	2.03 [1.00;4.41]	0.049
Defibrillation	24 (2.8)	8 (0.9)	-0.018	3.05 [1.41;7.35]	0.004
Vasoactive agents	31 (3.6)	29 (3.3)	-0.002		0.758
**Prehospital syndromic suspected condition**					
Ischemic heart disease	425 (49)	264 (30.1)	-0.189	0.45 [0.37;0.55]	<0.001
Acute heart failure	76 (8.8)	210 (23.9)	0.152	3.32 [2.51;4.43]	<0.001
Arrhythmia	112 (12.9)	116 (13.2)	0.001	1.02 [0.77;1.34]	0.961
Syncope	221 (25.2)	258 (29.4)	0.039	1.22 [0.99;1.50]	0.074
Hypertensive emergency	33 (3.8)	29 (3.3)	-0.005	0.86 [0.52;1.44]	0.664
**Hospital outcome**					
Inpatient	424 (48.9)	487 (55.5)	0.066	1.30 [1.08;1.58]	0.006
Hospitalization time, days	1 (0-6)	2 (0-7)	0.024	1.00 [0.99;1.01]	0.613
Echocardisocopy	335 (38.6)	303 (34.5)	-0.040	0.84 [0.69;1.02]	0.076
Fibrinolysis	26 (3)	10 (1.1)	-0.018	0.38 [0.17;0.77]	0.006
PIVS	249 (28.7)	157 (17.9)	-0.108	0.54 [0.43;0.68]	<0.001
Emergent surgery	21 (2.4)	18 (2.1)	-0.003	0.85 [0.44;1.60]	0.602
NIMV	26 (3)	70 (8)	0.049	2.79 [1.78;4.51]	<0.001
Mechanical ventilation	65 (7.5)	36 (4.1)	-0.033	0.53 [0.34;0.80]	<0.001
Vasoactive agents	63 (7.3)	55 (6.3)	-0.010	0.85 [0.59;1.24]	0.002
ACCU-admission	268 (30.9)	149 (19)	-0.139	0.46 [0.36;0.57]	<0.001

Abbreviations: NA: not applicable; Ref: reference; NIMV: noninvasive mechanical ventilation; PIVS: percutaneous interventional vascular surgery; ACCU: acute cardiac care unit.

[a]Values expressed as total number (percentage) and medians (25th-75th percentile), as appropriate.

[b]The Mann–Whitney U test or chi-squared test was used as appropriate.

cCohen's d-test was used to estimate the effect size.

[d]Fisher's exact probability statistic was used.

For peer review only

Table 3. Hazard ratio derived from Cox regression.

Variable	Hazard ratio	5% Confidence interval	95% Confidence interval	*p* value
Cohort ≥ 75 years	1.98	1.51	2.60	<0.001
Being in nursing home	1.42	1.07	1.89	0.014
Respiratory rate	1.02	1.01	1.04	<0.001
Systolic blood pressure	0.99	0.98	0.99	0.017
Lactate	1.25	1.20	1.30	<0.001
Noninvasive mechanical ventilation	1.83	1.26	2.65	0.001
Mechanical ventilation	2.53	1.30	4.92	0.006
Cardioversion	2.21	1.22	4.03	0.009
Defibrillation	2.39	1.23	4.64	0.010
Acute heart failure	2.32	1.63	3.31	<0.001
Age-Charlson comorbidity index	1.13	1.09	1.18	<0.001

For peer review only

For peer review only - http://bmjopen.bmj.com/site/about/guidelines.xhtml

Figure 1. Study population flowchart

Figure 2. Kaplan–Meier survival curves for age ≤ 74 years vs ≥ 75 years (A) and cardiovascular diagnosis for both groups together (B). Figure 1A, Age ≤ 74 years (red line) and age ≥ 75 years (blue line). Figure 1B, ischemic heart disease (red line), acute heart failure (dark green), arrhythmia (light green), syncope (blue line), hypertensive emergency (purple line).

For peer review only

For peer review only - http://bmjopen.bmj.com/site/about/guidelines.xhtml

Figure 1. Study population flowchart

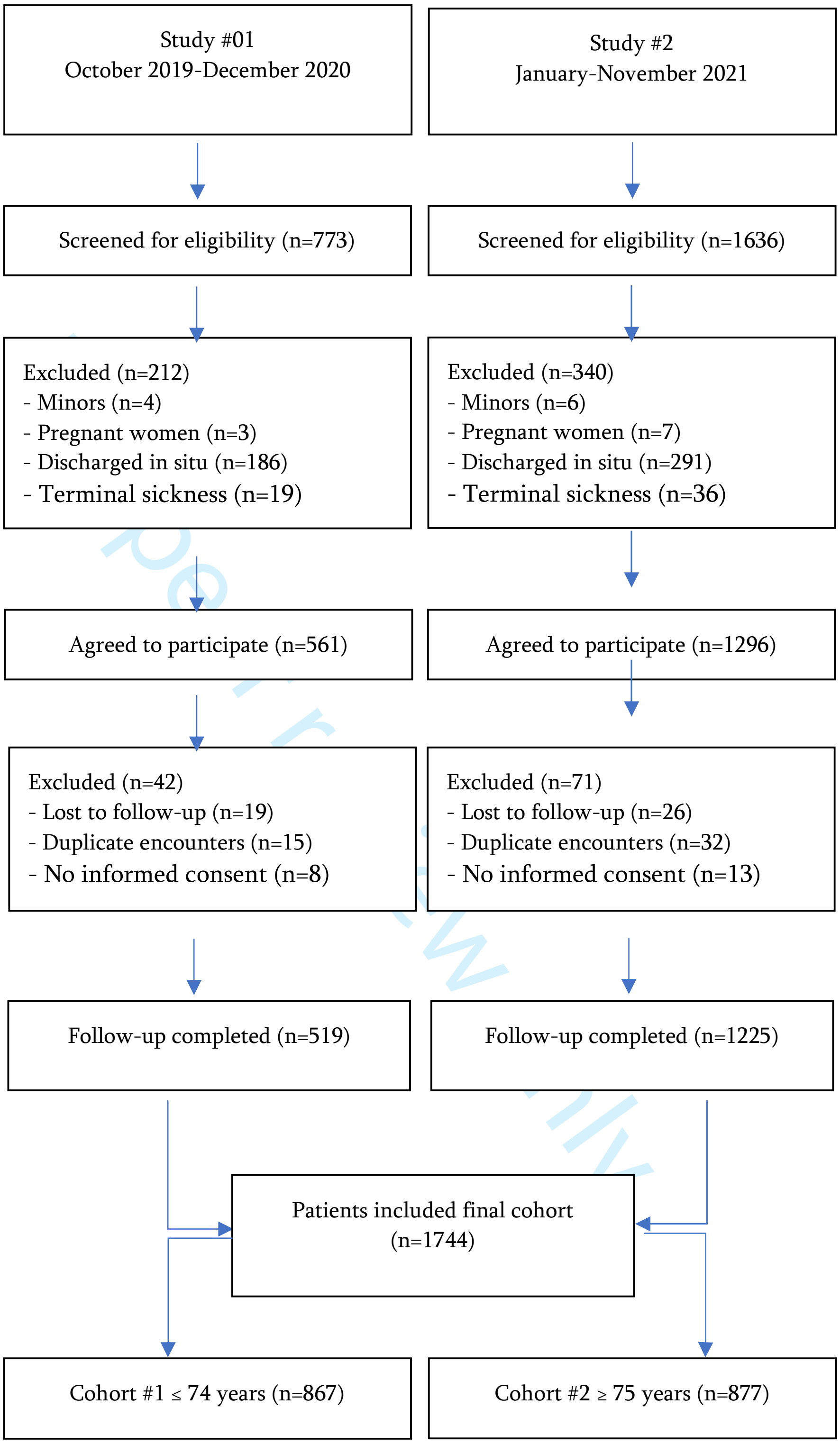

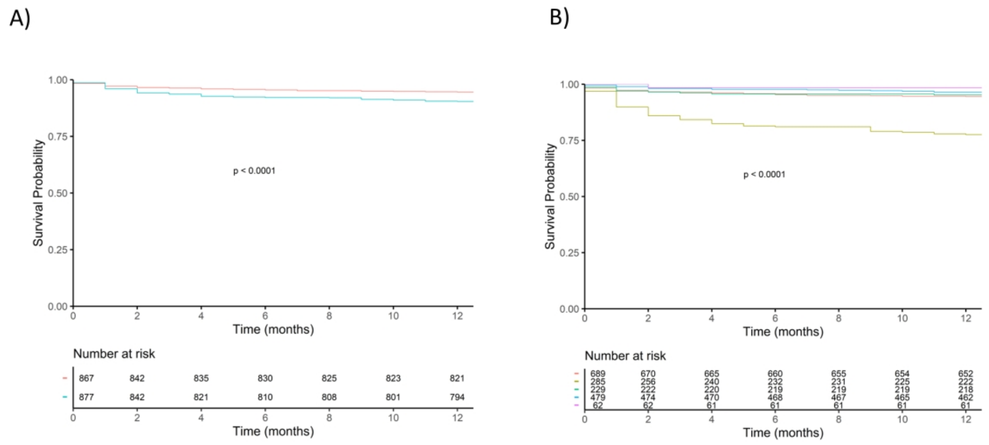

Figure 2. Kaplan–Meier survival curves for age ≤ 74 years vs ≥ 75 years (A) and cardiovascular diagnosis for both groups together (B). Figure 1A, Age ≤ 74 years (red line) and age ≥ 75 years (blue line). Figure 1B, ischemic heart disease (red line), acute heart failure (dark green), arrhythmia (light green), syncope (blue line), hypertensive emergency (purple line).

254x190mm (300 x 300 DPI)

Supplementary data

For peer review only

For peer review only - http://bmjopen.bmj.com/site/about/guidelines.xhtml

Table of contents

Supplementary methods…..……………………………………………………………..3
Strengthening the Reporting of Observational Studies in Epidemiology (STROBE) Statement…...………...……………………………………….….…3
Supplementary Results.……………………………………………………….……5
- Supplementary Figure 1. Kaplan Meier survival curves according to cardiovascular diagnosis and age……………………………………………...5
- Supplementary Table S1. Cumulative mortality by prehospital syndromic suspect…………………………………………………………………………6
- Supplementary Table S2: Summary descriptives table by groups of acute heart failure...…..……………………………………………………………...........7
- Supplementary Table S3: Summary descriptives table by groups of Arrythmmia..……………………………………………………………………8
- Supplementary Table S4: Summary descriptives table by groups of Hypertensive emergency……………………………..................................9
- Supplementary Table S5 Summary descriptives table by groups of ischemic heart disease……….……..…………………………………………………..10
- Supplementary Table S6: Summary descriptives table by groups of syncope..11
- Supplementary Table S7: Log-regression table by mortality groups…………12
References..14

For peer review only

Supplementary Methods

STROBE[1] Statement—checklist of items that should be included in reports of observational studies

	Item No	Recommendation	Page No
**Title and abstract**	1	(*a*) Indicate the study's design with a commonly used term in the title or the abstract	1
		(*b*) Provide in the abstract an informative and balanced summary of what was done and what was found	3
**Introduction**			
Background/rationale	2	Explain the scientific background and rationale for the investigation being reported	5
Objectives	3	State specific objectives, including any prespecified hypotheses	5
**Methods**			
Study design	4	Present key elements of study design early in the paper	6
Setting	5	Describe the setting, locations, and relevant dates, including periods of recruitment, exposure, follow-up, and data collection	6
Participants	6	(*a*) *Cohort study*—Give the eligibility criteria, and the sources and methods of selection of participants. Describe methods of follow-up<br>*Case-control study*—Give the eligibility criteria, and the sources and methods of case ascertainment and control selection. Give the rationale for the choice of cases and controls<br>*Cross-sectional study*—Give the eligibility criteria, and the sources and methods of selection of participants	6
		(*b*) *Cohort study*—For matched studies, give matching criteria and number of exposed and unexposed<br>*Case-control study*—For matched studies, give matching criteria and the number of controls per case	6
Variables	7	Clearly define all outcomes, exposures, predictors, potential confounders, and effect modifiers. Give diagnostic criteria, if applicable	7
Data sources/ measurement	8*	For each variable of interest, give sources of data and details of methods of assessment (measurement). Describe comparability of assessment methods if there is more than one group	7
Bias	9	Describe any efforts to address potential sources of bias	7
Study size	10	Explain how the study size was arrived at	7
Quantitative variables	11	Explain how quantitative variables were handled in the analyses. If applicable, describe which groupings were chosen and why	7
Statistical methods	12	(*a*) Describe all statistical methods, including those used to control for confounding	7
		(*b*) Describe any methods used to examine subgroups and interactions	7
		(*c*) Explain how missing data were addressed	7
		(*d*) *Cohort study*—If applicable, explain how loss to follow-up was addressed<br>*Case-control study*—If applicable, explain how matching of cases and controls was addressed<br>*Cross-sectional study*—If applicable, describe analytical methods taking account of sampling strategy	7
		(*e*) Describe any sensitivity analyses	7

Continued on next page

For peer review only

**Results**			
Participants	13*	(a) Report numbers of individuals at each stage of study—eg numbers potentially eligible, examined for eligibility, confirmed eligible, included in the study, completing follow-up, and analysed	8
		(b) Give reasons for non-participation at each stage	8
		(c) Consider use of a flow diagram	8
Descriptive data	14*	(a) Give characteristics of study participants (eg demographic, clinical, social) and information on exposures and potential confounders	8
		(b) Indicate number of participants with missing data for each variable of interest	8
		(c) *Cohort study*—Summarise follow-up time (eg, average and total amount)	8
Outcome data	15*	*Cohort study*—Report numbers of outcome events or summary measures over time	*8*
		Case-control study—Report numbers in each exposure category, or summary measures of exposure	*8*
		Cross-sectional study—Report numbers of outcome events or summary measures	*8*
Main results	16	(*a*) Give unadjusted estimates and, if applicable, confounder-adjusted estimates and their precision (eg, 95% confidence interval). Make clear which confounders were adjusted for and why they were included	9
		(*b*) Report category boundaries when continuous variables were categorized	9
		(*c*) If relevant, consider translating estimates of relative risk into absolute risk for a meaningful time period	9
Other analyses	17	Report other analyses done—eg analyses of subgroups and interactions, and sensitivity analyses	9
**Discussion**			
Key results	18	Summarise key results with reference to study objectives	10-11
Limitations	19	Discuss limitations of the study, taking into account sources of potential bias or imprecision. Discuss both direction and magnitude of any potential bias	11-12
Interpretation	20	Give a cautious overall interpretation of results considering objectives, limitations, multiplicity of analyses, results from similar studies, and other relevant evidence	10,11,12
Generalisability	21	Discuss the generalisability (external validity) of the study results	11,12,13
**Other information**			
Funding	22	Give the source of funding and the role of the funders for the present study and, if applicable, for the original study on which the present article is based	2

*Give information separately for cases and controls in case-control studies and, if applicable, for exposed and unexposed groups in cohort and cross-sectional studies.

Note: An Explanation and Elaboration article discusses each checklist item and gives methodological background and published examples of transparent reporting. The STROBE checklist is best used in conjunction with this article (freely available on the Web sites of PLoS Medicine at http://www.plosmedicine.org/, Annals of Internal Medicine at http://www.annals.org/, and Epidemiology at http://www.epidem.com/). Information on the STROBE Initiative is available at www.strobe-statement.org.

For peer review only

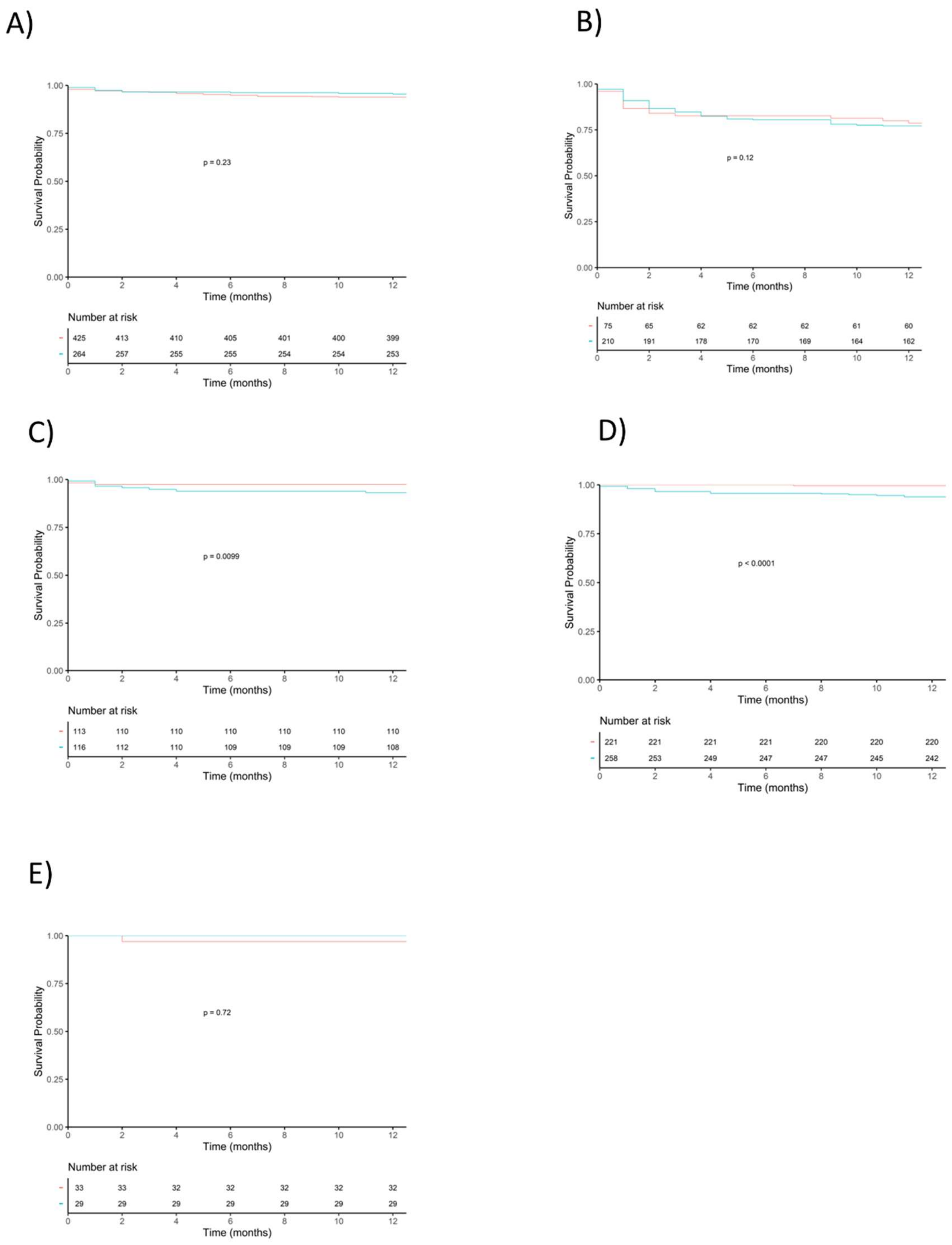

Supplementary Figure S1. Kaplan Meier survival curves for Age <75 years vs ≥ 75 years according to cardiovascular diagnosis. Ischemic heart disease (A), acute heart failure (B), arrhythmia (C), syncope (D), hypertensive emergency (E). Age ≤ 74 years (red line), and age ≥ 75 years (blue line).

Supplementary Table S1. Cumulative mortality by prehospital syndromic suspect

Variable[a]	Ischemic heart disease	Acute heart failure	Arrhythmia	Syncope	Hypertensive emergency	*p* value[b]
**≤ 74 years**	425 (49)	76 (8.8)	112 (12.9)	221 (25.2)	33 (3.8)	
1-day	12 (2.8)	10 (13.2)	3 (2.7)	0	0	0.022
2-day	14 (3.3)	12 (15.8)	3 (2.7)	0	1 (3)	0.033
7-day	24 (5.6)	13 (17.1)	3 (2.7)	1 (0.5)	1 (3)	0.002
30-day	26 (6.1)	21 (27.6)	3 (2.7)	2 (0.9)	1 (3)	0.002
90-day	33 (7.8)	26 (23.2)	7 (6.3)	7 (3.2)	1 (3)	0.012
180-day	38 (8.9)	28 (36.8)	9 (8)	10 (4.5)	3 (9.1)	0.060
365-day	43 (10.1)	33 (43.4)	10 (8.9)	12 (5.4)	3 (9.1)	0.042
**≥ 75 years**	264 (30.1)	210 (23.9)	116 (13.2)	258 (29.4)	29 (3.3)	
1-day	7 (2.7)	19 (9)	4 (3.4)	5 (1.9)	0	0.133
2-day	9 (3.4)	28 (13.3)	5 (4.3)	9 (3.5)	0	0.161
7-day	10 (3.8)	41 (19.5)	7 (6)	11 (4.3)	0	0.100
30-day	17 (6.4)	60 (28.6)	11 (9.5)	18 (7)	0	0.043
90-day	22 (8.3)	86 (41)	16 (13.8)	32 (12.4)	0	0.114
180-day	25 (9.5)	99 (47.1)	20 (17.2)	40 (15.5)	0	0.185
365-day	35 (13.3)	115 (54.8)	26 (22.4)	51 (19.8)	2 (6.9)	0.235

[a]Values expressed as total number (percentage).

[b]Fisher's Exact Probability statistic, was used.

For peer review only

Supplementary Table S2: Summary descriptives table by groups of acute heart failure

	Not Suffering acute heart failure *N=75*	Suffering acute heart failure *N=210*	OR	p.ratio	p.overall
1-day:					0.406
0	65 (86.7%)	191 (91.0%)	Ref.	Ref.	
1	10 (13.3%)	19 (9.05%)	0.64 [0.29;1.52]	0.304	
2-day:					0.706
0	63 (84.0%)	182 (86.7%)	Ref.	Ref.	
1	12 (16.0%)	28 (13.3%)	0.80 [0.39;1.74]	0.566	
7-day:					0.807
0	62 (82.7%)	169 (80.5%)	Ref.	Ref.	
1	13 (17.3%)	41 (19.5%)	1.15 [0.59;2.37]	0.693	
30-day:					1.000
0	54 (72.0%)	150 (71.4%)	Ref.	Ref.	
1	21 (28.0%)	60 (28.6%)	1.03 [0.57;1.88]	0.934	
90-day:					0.306
0	50 (66.7%)	124 (59.0%)	Ref.	Ref.	
1	25 (33.3%)	86 (41.0%)	1.38 [0.80;2.44]	0.249	
180-day:					0.125
0	48 (64.0%)	111 (52.9%)	Ref.	Ref.	
1	27 (36.0%)	99 (47.1%)	1.58 [0.92;2.75]	0.097	
365-day:					0.096
0	43 (57.3%)	95 (45.2%)	Ref.	Ref.	
1	32 (42.7%)	115 (54.8%)	1.62 [0.95;2.78]	0.074	

0: survival; 1: non-survival

Supplementary Table S3: Summary descriptives table by groups of Arrythmia

	Not Suffering Arrythmia N=113	Suffering Arrythmia N=116	OR	p.ratio	p.overall
1-day:					1.000
0	110 (97.3%)	112 (96.6%)	Ref.	Ref.	
1	3 (2.65%)	4 (3.45%)	1.29 [0.26;7.15]	0.748	
2-day:					0.722
0	110 (97.3%)	111 (95.7%)	Ref.	Ref.	
1	3 (2.65%)	5 (4.31%)	1.62 [0.37;8.55]	0.524	
7-day:					0.333
0	110 (97.3%)	109 (94.0%)	Ref.	Ref.	
1	3 (2.65%)	7 (6.03%)	2.28 [0.60;11.4]	0.233	
30-day:					0.060
0	110 (97.3%)	105 (90.5%)	Ref.	Ref.	
1	3 (2.65%)	11 (9.48%)	3.69 [1.10;17.5]	0.034	
90-day:					0.149
0	105 (92.9%)	100 (86.2%)	Ref.	Ref.	
1	8 (7.08%)	16 (13.8%)	2.07 [0.86;5.38]	0.103	
180-day:					0.092
0	103 (91.2%)	96 (82.8%)	Ref.	Ref.	
1	10 (8.85%)	20 (17.2%)	2.12 [0.96;4.99]	0.063	
365-day:					0.015
0	102 (90.3%)	90 (77.6%)	Ref.	Ref.	
1	11 (9.73%)	26 (22.4%)	2.65 [1.26;5.91]	0.010	

0: survival; 1: non-survival

Supplementary Table S4: Summary descriptives table by groups of Hypertensive Emergency

	Not Suffering Hypertensive Emergency N=33	Suffering Hypertensive Emergency N=29	OR	p.ratio	p.overall
1-day: 0	33 (100%)	29 (100%)	Ref.	Ref.	.
2-day:					1.000
0	32 (97.0%)	29 (100%)	Ref.	Ref.	
1	1 (3.03%)	0 (0.00%)	. [.;.]	.	
7-day:					1.000
0	32 (97.0%)	29 (100%)	Ref.	Ref.	
1	1 (3.03%)	0 (0.00%)	. [.;.]	.	
30-day:					1.000
0	32 (97.0%)	29 (100%)	Ref.	Ref.	
1	1 (3.03%)	0 (0.00%)	. [.;.]	.	
90-day:					1.000
0	32 (97.0%)	29 (100%)	Ref.	Ref.	
1	1 (3.03%)	0 (0.00%)	. [.;.]	.	
180-day:					0.241
0	30 (90.9%)	29 (100%)	Ref.	Ref.	
1	3 (9.09%)	0 (0.00%)	. [.;.]	.	
365-day:					1.000
0	30 (90.9%)	27 (93.1%)	Ref.	Ref.	
1	3 (9.09%)	2 (6.90%)	0.76 [0.08;5.36]	0.782	

0: survival; 1: non-survival

For peer review only - http://bmjopen.bmj.com/site/about/guidelines.xhtml

Supplementary Table S5: Summary descriptives table by groups of ischemic heart disease

	Not suffering ischemic heart disease N=425	Suffering ischemic heart disease N=264	OR	p.ratio	p.overall
1-day:					1.000
0	413 (97.2%)	257 (97.3%)	Ref.	Ref.	
1	12 (2.82%)	7 (2.65%)	0.95 [0.34;2.41]	0.910	
2-day:					1.000
0	411 (96.7%)	255 (96.6%)	Ref.	Ref.	
1	14 (3.29%)	9 (3.41%)	1.04 [0.42;2.43]	0.925	
7-day:					0.360
0	401 (94.4%)	254 (96.2%)	Ref.	Ref.	
1	24 (5.65%)	10 (3.79%)	0.66 [0.30;1.38]	0.280	
30-day:					0.994
0	399 (93.9%)	247 (93.6%)	Ref.	Ref.	
1	26 (6.12%)	17 (6.44%)	1.06 [0.55;1.98]	0.859	
90-day:					0.902
0	392 (92.2%)	242 (91.7%)	Ref.	Ref.	
1	33 (7.76%)	22 (8.33%)	1.08 [0.61;1.89]	0.785	
180-day:					0.922
0	387 (91.1%)	239 (90.5%)	Ref.	Ref.	
1	38 (8.94%)	25 (9.47%)	1.07 [0.62;1.81]	0.811	
365-day:					0.254
0	382 (89.9%)	229 (86.7%)	Ref.	Ref.	
1	43 (10.1%)	35 (13.3%)	1.36 [0.84;2.18]	0.211	

0: survival; 1: non-survival

Supplementary Table S6: Summary descriptives table by groups of syncope

	**Not suffering syncope** *N=221*	**Suffering syncope** *N=258*	**OR**	**p.ratio**	**p.overall**
1-day:					0.065
0	221 (100%)	253 (98.1%)	Ref.	Ref.	
1	0 (0.00%)	5 (1.94%)	. [.;.]	.	
2-day:					0.004
0	221 (100%)	249 (96.5%)	Ref.	Ref.	
1	0 (0.00%)	9 (3.49%)	. [.;.]	.	
7-day:					0.018
0	220 (99.5%)	247 (95.7%)	Ref.	Ref.	
1	1 (0.45%)	11 (4.26%)	8.65 [1.65;214]	0.007	
30-day:					0.002
0	219 (99.1%)	240 (93.0%)	Ref.	Ref.	
1	2 (0.90%)	18 (6.98%)	7.67 [2.16;52.7]	0.001	
90-day:					<0.001
0	214 (96.8%)	226 (87.6%)	Ref.	Ref.	
1	7 (3.17%)	32 (12.4%)	4.24 [1.93;10.8]	<0.001	
180-day:					<0.001
0	211 (95.5%)	218 (84.5%)	Ref.	Ref.	
1	10 (4.52%)	40 (15.5%)	3.82 [1.93;8.31]	<0.001	
365-day:					<0.001
0	209 (94.6%)	207 (80.2%)	Ref.	Ref.	
1	12 (5.43%)	51 (19.8%)	4.24 [2.26;8.58]	<0.001	

0: survival; 1: non-survival

Supplementary Table S7: Log-regression table by mortality groups

	**Survival**	**Non-Survival**	**Hazard Ratio [95% Confidence interval]**	**p.value**
	N=1414	***N=330***		
Cohort:				
≤ 74 years	766 (54.2%)	101 (30.6%)	Ref.	Ref.
≥ 75 years	648 (45.8%)	229 (69.4%)	2.41 [1.91;3.04]	<0.001
Sex:				
Male	805 (56.9%)	214 (64.8%)	Ref.	Ref.
Female	609 (43.1%)	116 (35.2%)	0.74 [0.59;0.93]	0.010
Nursing home				
No	1297 (91.7%)	257 (77.9%)	Ref.	Ref.
Yes	117 (8.27%)	73 (22.1%)	2.69 [2.07;3.49]	<0.001
Respiratory Rate	17.9 (6.35)	22.1 (10.5)	1.06 [1.05;1.07]	<0.001
SpO2	95.7 (5.23)	87.2 (13.0)	0.94 [0.93;0.94]	<0.001
SBP	138 (32.7)	123 (40.8)	0.99 [0.98;0.99]	<0.001
DBP	79.0 (19.1)	69.6 (24.5)	0.98 [0.97;0.98]	<0.001
Heart rate	83.5 (31.8)	95.1 (42.9)	1.01 [1.01;1.01]	<0.001
Temperature	36.1 (0.68)	36.1 (0.98)	0.94 [0.81;1.10]	0.424
GCS	14.8 (1.25)	12.8 (4.00)	0.79 [0.77;0.81]	<0.001
Glucose	141 (57.3)	191 (90.8)	1.01 [1.00;1.01]	<0.001
Lactate	1.99 (1.35)	4.63 (3.79)	1.37 [1.33;1.40]	<0.001
NIMV:				
No	1387 (98.1%)	270 (81.8%)	Ref.	Ref.
Yes	27 (1.91%)	60 (18.2%)	6.85 [5.17;9.08]	<0.001
Mechanical ventilation:				
No	1404 (99.3%)	278 (84.2%)	Ref.	Ref.
Yes	10 (0.71%)	52 (15.8%)	14.5 [10.7;19.6]	<0.001
Transcutaneous pacemaker:				

	Survival	Non-Survival	Hazard Ratio [95% Confidence interval]	p.value
	N=1414	*N=330*		
No	1364 (96.5%)	320 (97.0%)	Ref.	Ref.
Yes	50 (3.54%)	10 (3.03%)	0.91 [0.48;1.70]	0.759
Cardioversion:				
No	1396 (98.7%)	315 (95.5%)	Ref.	Ref.
Yes	18 (1.27%)	15 (4.55%)	3.24 [1.93;5.43]	<0.001
Defibrillation.:				
No	1406 (99.4%)	306 (92.7%)	Ref.	Ref.
Yes	8 (0.57%)	24 (7.27%)	9.04 [5.95;13.7]	<0.001
Vasoactive agents:				
No	1402 (99.2%)	282 (85.5%)	Ref.	Ref.
Yes	12 (0.85%)	48 (14.5%)	13.4 [9.81;18.2]	<0.001
Prehospital syndromic suspect:				
Ischemic heart disease	611 (43.2%)	78 (23.6%)	Ref.	Ref.
Acute heart failure	138 (9.76%)	147 (44.5%)	6.01 [4.57;7.92]	<0.001
Arrhythmia	192 (13.6%)	37 (11.2%)	1.46 [0.98;2.15]	0.060
Syncope	416 (29.4%)	63 (19.1%)	1.16 [0.83;1.61]	0.394
Hypertensive emergency	57 (4.03%)	5 (1.52%)	0.68 [0.28;1.69]	0.412
ACCI	2.31 (2.33)	4.29 (2.96)	1.23 [1.19;1.27]	<0.001

For peer review only - http://bmjopen.bmj.com/site/about/guidelines.xhtml

References

1. Vandenbroucke JP, von Elm E, Altman DG, Gøtzsche PC, Mulrow CD, Pocock SJ, Poole C, Schlesselman JJ, Egger M; STROBE Initiative. Strengthening the Reporting of Observational Studies in Epidemiology (STROBE): explanation and elaboration. PLoS Med. 2007 Oct 16;4(10):e297.

For peer review only

For peer review only - http://bmjopen.bmj.com/site/about/guidelines.xhtml

STROBE[1] Statement—checklist of items that should be included in reports of observational studies

	Item No	Recommendation	Page No
**Title and abstract**	1	(*a*) Indicate the study's design with a commonly used term in the title or the abstract	1
		(*b*) Provide in the abstract an informative and balanced summary of what was done and what was found	3
**Introduction**			
Background/rationale	2	Explain the scientific background and rationale for the investigation being reported	5
Objectives	3	State specific objectives, including any prespecified hypotheses	5
**Methods**			
Study design	4	Present key elements of study design early in the paper	6
Setting	5	Describe the setting, locations, and relevant dates, including periods of recruitment, exposure, follow-up, and data collection	6
Participants	6	(*a*) *Cohort study*—Give the eligibility criteria, and the sources and methods of selection of participants. Describe methods of follow-up<br>*Case-control study*—Give the eligibility criteria, and the sources and methods of case ascertainment and control selection. Give the rationale for the choice of cases and controls<br>*Cross-sectional study*—Give the eligibility criteria, and the sources and methods of selection of participants	6
		(*b*) *Cohort study*—For matched studies, give matching criteria and number of exposed and unexposed<br>*Case-control study*—For matched studies, give matching criteria and the number of controls per case	6
Variables	7	Clearly define all outcomes, exposures, predictors, potential confounders, and effect modifiers. Give diagnostic criteria, if applicable	7
Data sources/ measurement	8*	For each variable of interest, give sources of data and details of methods of assessment (measurement). Describe comparability of assessment methods if there is more than one group	7
Bias	9	Describe any efforts to address potential sources of bias	7
Study size	10	Explain how the study size was arrived at	7
Quantitative variables	11	Explain how quantitative variables were handled in the analyses. If applicable, describe which groupings were chosen and why	7
Statistical methods	12	(*a*) Describe all statistical methods, including those used to control for confounding	7
		(*b*) Describe any methods used to examine subgroups and interactions	7
		(*c*) Explain how missing data were addressed	7
		(*d*) *Cohort study*—If applicable, explain how loss to follow-up was addressed<br>*Case-control study*—If applicable, explain how matching of cases and controls was addressed<br>*Cross-sectional study*—If applicable, describe analytical methods taking account of sampling strategy	7
		(*e*) Describe any sensitivity analyses	7

Continued on next page

For peer review only - http://bmjopen.bmj.com/site/about/guidelines.xhtml

**Results**			
Participants	13*	(a) Report numbers of individuals at each stage of study—eg numbers potentially eligible, examined for eligibility, confirmed eligible, included in the study, completing follow-up, and analysed	8
		(b) Give reasons for non-participation at each stage	8
		(c) Consider use of a flow diagram	8
Descriptive data	14*	(a) Give characteristics of study participants (eg demographic, clinical, social) and information on exposures and potential confounders	8
		(b) Indicate number of participants with missing data for each variable of interest	8
		(c) *Cohort study*—Summarise follow-up time (eg, average and total amount)	8
Outcome data	15*	*Cohort study*—Report numbers of outcome events or summary measures over time	*8*
		Case-control study—Report numbers in each exposure category, or summary measures of exposure	*8*
		Cross-sectional study—Report numbers of outcome events or summary measures	*8*
Main results	16	(*a*) Give unadjusted estimates and, if applicable, confounder-adjusted estimates and their precision (eg, 95% confidence interval). Make clear which confounders were adjusted for and why they were included	9
		(*b*) Report category boundaries when continuous variables were categorized	9
		(*c*) If relevant, consider translating estimates of relative risk into absolute risk for a meaningful time period	9
Other analyses	17	Report other analyses done—eg analyses of subgroups and interactions, and sensitivity analyses	9
**Discussion**			
Key results	18	Summarise key results with reference to study objectives	10-11
Limitations	19	Discuss limitations of the study, taking into account sources of potential bias or imprecision. Discuss both direction and magnitude of any potential bias	11-12
Interpretation	20	Give a cautious overall interpretation of results considering objectives, limitations, multiplicity of analyses, results from similar studies, and other relevant evidence	10,11,12
Generalisability	21	Discuss the generalisability (external validity) of the study results	11,12,13
**Other information**			
Funding	22	Give the source of funding and the role of the funders for the present study and, if applicable, for the original study on which the present article is based	2

*Give information separately for cases and controls in case-control studies and, if applicable, for exposed and unexposed groups in cohort and cross-sectional studies.

Note: An Explanation and Elaboration article discusses each checklist item and gives methodological background and published examples of transparent reporting. The STROBE checklist is best used in conjunction with this article (freely available on the Web sites of PLoS Medicine at http://www.plosmedicine.org/, Annals of Internal Medicine at http://www.annals.org/, and Epidemiology at http://www.epidem.com/). Information on the STROBE Initiative is available at www.strobe-statement.org.

For peer review only
